# The transcriptome dynamics of single cells during the cell cycle

**DOI:** 10.15252/msb.20209946

**Published:** 2020-11-18

**Authors:** Daniel Schwabe, Sara Formichetti, Jan Philipp Junker, Martin Falcke, Nikolaus Rajewsky

**Affiliations:** ^1^ Mathematical Cell Physiology Max Delbrück Center for Molecular Medicine in the Helmholtz Association Berlin Germany; ^2^ Systems Biology of Gene Regulatory Elements, Berlin Institute for Medical Systems Biology Max Delbrück Center for Molecular Medicine in the Helmholtz Association Berlin Germany; ^3^ Epigenetics and Neurobiology Unit, European Molecular Biology Laboratory Monterotondo Italy; ^4^ Collaboration for Joint PhD Degree between European Molecular Biology Laboratory and Heidelberg University, Faculty of Biosciences Heidelberg Germany; ^5^ Quantitative Developmental Biology, Berlin Institute for Medical Systems Biology Max Delbrück Center for Molecular Medicine in the Helmholtz Association Berlin Germany; ^6^ Department of Physics Humboldt University Berlin Berlin Germany

**Keywords:** cell biology, cell cycle, dynamical systems, single‐cell RNA sequencing, systems biology, Cell Cycle, Chromatin, Epigenetics, Genomics & Functional Genomics, Computational Biology

## Abstract

The cell cycle is among the most basic phenomena in biology. Despite advances in single‐cell analysis, dynamics and topology of the cell cycle in high‐dimensional gene expression space remain largely unknown. We developed a linear analysis of transcriptome data which reveals that cells move along a planar circular trajectory in transcriptome space during the cycle. Non‐cycling gene expression adds a third dimension causing helical motion on a cylinder. We find in immortalized cell lines that cell cycle transcriptome dynamics occur largely independently from other cellular processes. We offer a simple method (“Revelio”) to order unsynchronized cells in time. Precise removal of cell cycle effects from the data becomes a straightforward operation. The shape of the trajectory implies that each gene is upregulated only once during the cycle, and only two dynamic components represented by groups of genes drive transcriptome dynamics. It indicates that the cell cycle has evolved to minimize changes of transcriptional activity and the related regulatory effort. This design principle of the cell cycle may be of relevance to many other cellular differentiation processes.

## Graphical abstract

Deep single‐cell RNA sequencing of asynchronous immortalized cycling cells is leveraged to analyze the cell cycle in high‐dimensional gene expression space. Principles of dynamical systems theory are utilized to define a cell cycle trajectory and to seek for underlying design principles.

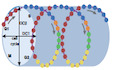

## Introduction

The cell cycle is a shared and general principle of life. Core aspects of the cell cycle are conserved across eukaryotes (Morgan, 2006; Alberts, 2015). However, as cell division rates vary massively across species and cell types, the cell cycle also needs to be plastic and coupled to cellular physiology. The active components of cell cycle dynamics in gene expression space are groups of genes. Despite a multitude of mechanistic studies and insights into the oscillatory nature of gene expression caused by the cell cycle (Alter *et al*, 2000; Buettner *et al*, 2015; Liu *et al*, 2017b), the topology (or shape) of the cell cycle within gene expression space, as well as its degree of coupling to transcriptome dynamics of other cellular processes, remains largely unclear (Tanay & Regev, 2017; Liu *et al*, 2017a). Neither is it known, whether a two‐component oscillator is sufficient to describe the complete cell cycle, whether additional components are needed, or whether optimality principles govern gene expression changes along the cell cycle.

The progression of a cell through the cell cycle can be represented as a trajectory in transcriptome space. In recent years, pseudo‐temporal ordering of single‐cell transcriptomes has emerged as a powerful method for reconstruction of low‐dimensional cell differentiation trajectories from high‐dimensional single‐cell RNA‐seq data (Kester & van Oudenaarden, 2018). We examine transcriptomic snapshots of populations of asynchronous cycling cells in order to reconstruct, quantify, and interpret the cell cycle as a dynamical system, to define its trajectory and to seek for underlying design principles. The analysis is limited to information on transcriptional regulation along the cell cycle. The data do not hold information on other well‐known cell cycle mechanisms such as regulation by phosphatases and kinases.

**Figure 1:**
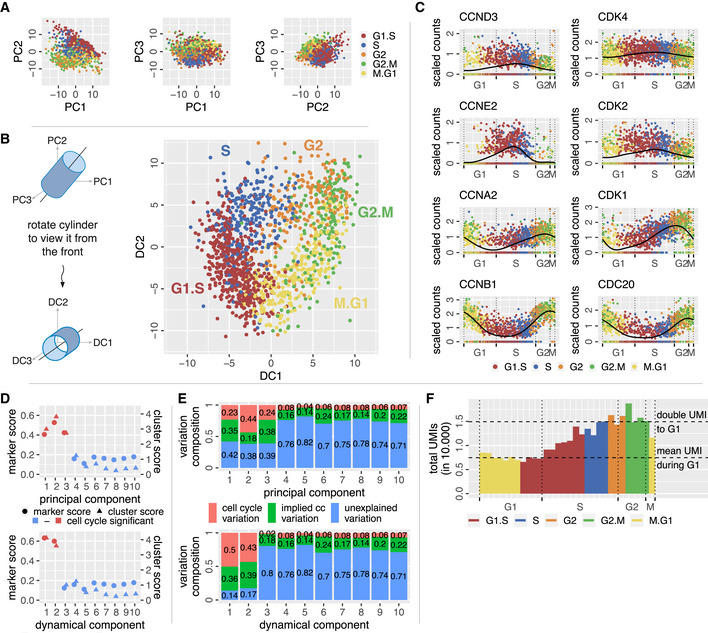
The cell cycle forms an annulus in two dimensions and a cylinder in three dimensions of transcriptome space Two‐dimensional representations of the first three PC scores of all individual cells (HeLaS3 cell line, 1,348 cells) exhibit clustering with regard to their computationally inferred cell cycle phases. A clear cyclic structure is absent.In three dimensions, the data form a slanted cylinder. A clear cell cycle structure becomes visible if we rotate the cylinder and view it from the top or base. The rotated PCs are simple linear combinations of the original PCs and are the dynamical components (DCs). DC1 and DC2 span the cell cycle.The angular coordinate of cells around the cell cycle provides the order in time. We show time courses for eight genes that are known to form the backbone of the cell cycle.We measure the amount of cell cycle within each principal (top) and dynamical (bottom) component by a marker score and a cluster score (see Materials and Methods). Both scores indicate three components to be involved in cell cycle in PC space, while only two components are marked as significant in DC space.The variation of the data within each principal (top) and dynamical (bottom) component is decomposed into different sources (see Materials and Methods). The portion of unexplained variation in DC1 and DC2 is much lower than for PC1, PC2, and PC3. Variation in DC3 is almost not at all explained by cell cycle genes.Summing up all UMIs according to the pseudotime of cells yields a clear drop by factor 1/2 at the end of the cycle (transition from the G2.M to M.G1 cluster). This is where cell division is assumed to happen.

We expect the trajectory in transcriptome space to describe a periodic motion, completed once each time a cell divides. We anticipate the trajectory to be constrained to a subspace with much lower dimension than the transcriptome space (~20,000 dimensions) because only a subset of genes is involved in the cell cycle and genes are known to interact in a highly coordinated manner (Morgan, 2006; Alberts *et al*., 2015), i.e., groups of genes controlled by transcription factors and chromatin state are up‐ or downregulated together during the cycle (Voss & Hager, 2013). A priori, the transcriptomic trajectory describing the cell cycle might be a simple circle embedded in a plane, it might be wound up on a donut‐like structure (torus), twisted, and looped like a roller coaster in three dimensions or be even more complex in higher dimensions (Box Fig 1). The number of dimensions required to embed the cell cycle trajectory is an upper bound for the number of independent components driving its dynamics (Arnold, 1992). The regulatory effort required to complete the cell cycle is closely related to the shape of the trajectory in transcriptome space (as explained in Box 1). In general, the simpler the geometric shape the less regulatory effort is required.

Due to cell‐to‐cell variability, cell cycle trajectories of individual cells of the same cell type will not be identical and aligned. The collection of trajectories from a population of cells can be imagined as a tube in transcriptome space encompassing all trajectories. This tube is called a manifold, and the volume of this manifold contains information on cell variability. We first set out to formally define the cell cycle manifold and then to identify trajectories within it with an RNA velocity analysis.

Box 1 **The transcriptome as a dynamical system**

In the context of our considerations, the state of the transcriptome is completely described by the molecule copy number of all species of transcripts in the cell. We can represent the state of the transcriptome of a single cell in a coordinate system with as many axes as there are species of transcripts. The state of the transcriptome is a point in this high‐dimensional space. The cell changes its transcriptomic state again and again over time. Hence, among its many other aspects, the transcriptome is also a dynamic system. Change of state is motion along a trajectory in transcriptome space.

We recollect two general results of dynamic systems theory here. Firstly, the trajectory of a deterministic dynamical system cannot intersect with itself (Arnold, 1992). Secondly, the minimum number of dimensions required to embed a trajectory (in conforming with the first point) is a lower bound for the number of ordinary differential equations required to describe the dynamics (Arnold, 1992; Kuznetsov, 1998), or in other words a lower bound for the number of independent players shaping the trajectory.

Trajectories of a periodic process are closed trajectories. In Box Fig 1, we show examples of such trajectories in a two‐dimensional transcriptome space and a three‐dimensional space—two respectively three genes participate in these toy dynamics. We consider first the example Box Fig 1A. Completing it implies regulating gene X up and down once, and the same for gene Y. Box Fig 1B is a cartoon of an extreme case (a star). It requires partial up‐ and down‐regulation of both genes 6 times for completing the cycle—many more up‐ and down regulations than the number of participating genes.

The first three‐dimensional example (Box Fig 1C) is more complicated than a circle. It is a type of trajectory found in many dynamical systems and requires at least three dimensions to embed it. The trajectory consists of two loops distinguished mainly by the value of Z. Completing this trajectory once requires more regulation of gene expression than with a circle. It implies regulating gene Z up and down once. Genes X and Y are regulated up and down twice—one time in the "lower" loop and one time in the "upper" loop. Thus, completing this trajectory requires more transcriptional activation and termination than a simple circle.

Box Fig 1D is called a torus, and again a common type of trajectory requiring three dimensions to embed it. It also implies more transcription initiations and terminations per cycle than the number of participating genes. The last example (Box Fig 1E) is an extreme cartoon again, but given the high dimensionality of the transcriptome space, it is a reasonable possibility.

These considerations clearly show there is a relation between the shape of the trajectory in transcriptome space and the regulatory effort—the number of up‐ and down regulations of a given gene—required to complete the cycle.

If the trajectory in a high‐dimensional space runs on a circle in the side of a cylinder, the trajectory entails only as many up‐ and down regulations as there are participating genes. Such a trajectory can also be embedded in a two‐dimensional state space. However, the axes do not have the meaning of the number of transcripts of a single gene anymore but describe the amplitude of a group of genes like a principal component or a dynamical component resulting from our analysis (see main text). The genes within one group are regulated in a highly coordinated way but not necessarily synchronously.

We find the cell cycle trajectory in a plane in transcriptome space, i.e., it takes two dimensions to embed it (see main text). This is the minimal number of dimensions required for periodic motion. Hence, essentially two groups of genes interact to drive the cell cycle. The composition of our dynamical components DC1 and DC2 represents a suggestion for these groups (Fig EV3). Together, they comprise 266 genes with significant weights for the HeLa data set 1.1 (Appendix Table S1), 39 of them are found across all three cell types investigated (HeLa, HEK, 3T3). Positive weights in DC1 correspond to M phase genes, whereas negative weights in DC1 are strongly associated with S phase genes. Simultaneously, genes with positive weights in DC2 are highly correlated to G2 phase, while negative weights are mostly absent in evidence of little cyclic activity at the middle of G1 phase. Consequently, only DC1 contains transcripts for cyclin B (a well‐known M phase protein) with positive weights and cyclin E (activated during G1‐S transition) in antiphase with negative weight. DC1 also contains transcripts of cyclin A, which is highly expressed during M phase as well as G2 phase. The latter causes it to also have a significant contribution to DC2. The feedbacks between the cyclins mediated by cyclin‐dependent kinases and other factors represent one of the interactions between DC1 and DC2. Cyclin B1 or B1 and B2 have been shown to be essential for the cell cycle (Brandeis *et al*, 1998; Soni *et al*, 2008; Strauss *et al*, 2018), suggesting that the cyclin network is the only mechanism able to drive cells completely through the cycle. That is in line with the simplicity of the cell cycle trajectory observed in this study.

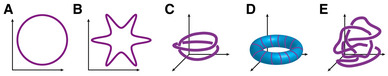

**Box Figure 1**. **Toy examples of possible shapes of the cell cycle trajectories in transcriptome space**.

- A circle in two dimensions.
- A star.
- A cyclic trajectory requiring three dimensions with an upper and a lower loop.
- A torus.
- A three‐dimensional motion comparable to a roller coaster.

## Results

A HeLaS3 cell line was grown asynchronously and single‐cell RNA sequenced deeply using an in‐house optimized version of the Drop‐seq protocol (Macosko *et al*, 2015; Alles *et al*, 2017; Materials and Methods). The data set contains single‐cell data of 1477 cells with a mean depth of roughly 11,000 unique molecular identifiers (UMIs) per cell (Appendix Table S1). We computationally inferred a cell cycle phase for each single cell by correlating its transcriptome data to known marker genes for different parts of the cell cycle (in particular G1.S, S, G2, G2.M, and M.G1; Whitfield *et al*, 2002; Macosko *et al.*, 2015; Materials and Methods). We restricted the data to 1031 detected highly variable genes. Furthermore, we transformed the data by calculating concentrations, multiplying by a scaling factor, log‐transforming it, and normalizing it across all genes and all cells (Butler *et al*, 2018; Materials and Methods). While there is a large number of tools for pseudo‐temporal ordering of single‐cell transcriptomes [e.g. reCAT (Liu *et al.*, 2017b), Oscope (Leng *et al*, 2015), Monocle (Trapnell *et al*, 2014), Wanderlust (Bendall *et al*, 2014), Wishbone (Setty *et al*, 2016), and PAGA (Wolf *et al*, 2019)], these mostly specialize in non‐linear manifold learning approaches. We show that a linear approach is sufficient to isolate the cell cycle, which substantially facilitates downstream analysis and interpretation by preserving the geometric structure of the trajectory.

After applying principal component analysis (PCA) to our data, we observed that the first three principal components (PCs) exhibit cell clustering according to the computationally inferred cell cycle phases (Fig 1A). Additional PCs do not reflect the cell cycle (Fig 1D). None of the lines of view on the data parallel to the PC axes shown in Fig 1A exhibit a clear periodic trajectory, suggesting that PC axes are not the line of view revealing the cycle and that at least three PC dimensions are necessary to define the cell cycle manifold.

Rotating our line of view revealed the data cloud to form a cylinder which is slanted with respect to PC axes. Viewing it from the top or base yielded an annular shape in two dimensions that provides a surprisingly good representation of the cell cycle (Fig 1B). Most notably, we obtained clear clustering and progression of cell cycle phases, which displays the expected order G1‐S‐G2‐M. Additionally, we observed an area around the origin that is much less populated with data points, in agreement with the principle that cells cannot skip phases. When viewed from the appropriate angle, the cell cycle is in fact contained in a two‐dimensional plane.

**Figure EV1:**
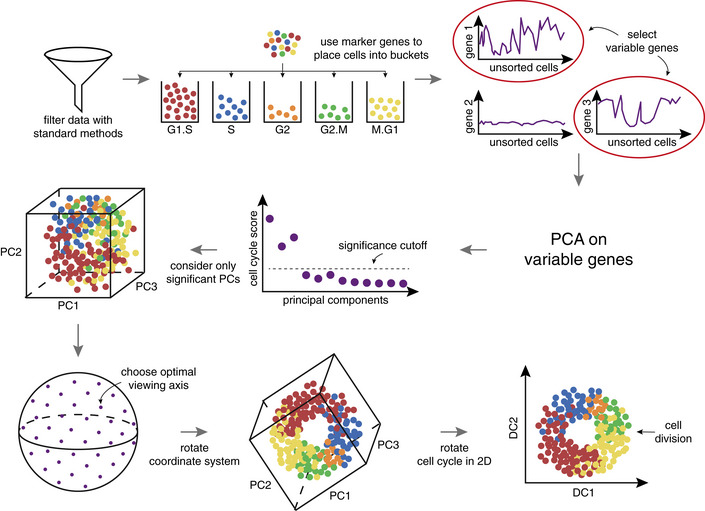
The principle steps of our algorithm Revelio for extracting the cell cycle from the data After the data are filtered by standard methods, we divide the cells into buckets with the help of marker genes (Whitfield *et al.*, 2002; Macosko *et al.*, 2015). Next, we select variable genes (Butler *et al.*, 2018) and apply PCA on the reduced data set. Afterward, we utilize a cell cycle marker and cluster score (Materials and Methods) to judge which PCs are influenced by the cell cycle. The significant PCs are used to construct three‐dimensional subspaces. We then choose an optimal viewing axis by minimizing the cell cycle score along the viewing axis (Materials and Methods). The coordinate system is rotated linearly and the cell cycle is obtained only within the plane spanned by the first two new axes (DC1, DC2). Within the DC1‐DC2 plane, we estimate the time point of cell division and rotate the cell cycle plane accordingly (Materials and Methods).

The change of angle of view is achieved by a basic linear rotation of the PC space (imagine rotating a cube and viewing it from different angles, see Materials and Methods). The newly found axes after rotation—which we refer to as dynamical components (DCs)—are linear combinations of the PCs involved in the rotation (Materials and Methods). The general steps of our algorithm Revelio (REVEaling the cell cycle with a LInear Operator) from raw data to the two‐dimensional cell cycle are outlined in Fig EV1.

**Figure EV2:**
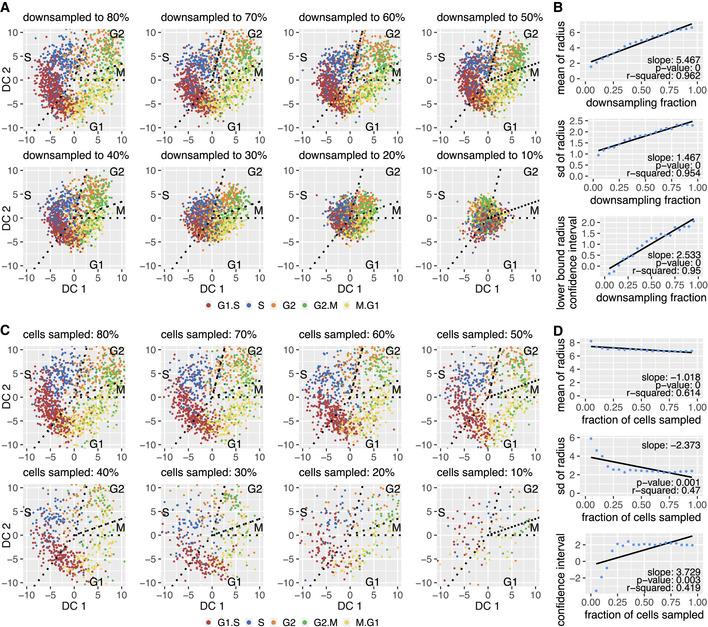
Downsampling the UMIs leads to a continuously collapsing cell cycle while sampling cells maintains the cycle until a threshold is reached We downsample (Griffiths *et al.*, 2018; Lun *et al.*, 2019) the data set to different percentages of the total UMI for each cell and run Revelio on the new data. The cell cycle is slowly collapsing toward the origin with decreasing information.Statistics on the radius of the data points seen in panel A confirm the previous conclusion. (top) Mean of the radius. (middle) Standard deviation of the radius. (bottom) The lower bound of the confidence interval of the radius. For all of these characteristics, a clear linear relationship to the downsampling percentage is apparent.We now sample a certain percentage of the cells while keeping the information within each cell untouched. The cell cycle now does not appear to collapse anymore but becomes less clear with fewer cells available.The same characteristics as in panel B now suggest that the cell cycle stays intact and in principle unchanged as long as at least 25% of the initial amount of cells are maintained. Afterward, the circular cell cycle signal breaks down rapidly with the decreasing amount of cells.

We were able to reproduce the results with other cell lines, including an additional HeLaS3, a HEK293, and a 3T3 data set where the form of the cell cycle varies from an annulus to a disc (Appendix Fig S1–S3). We also managed to isolate the cell cycle into an annulus in just two dimensions even when utilizing all genes detected during sequencing (~10,000 genes) instead of only the highly variable genes (Appendix Fig S4). Hence, inclusion of additional genes into this specific analysis, which typically increases noise levels, does not alter the characteristics of the outcome. The limiting factor appears to be rather the sequencing depth because the less deeply sequenced a data set is, the more noise is incorporated due to, e.g., increased amounts of dropouts. As a rule of thumb, we estimate that either at least 600 cells with an average depth of 4,500 UMIs or at least 1,000 cells with an average depth of 3,000 UMIs are required for the cell cycle pattern to reveal itself (Fig EV2, Materials and Methods).

Conversely, we ruled out that cells near the origin in our deeply sequenced data sets are simply dead cells by considering mitochondrial amount and apoptotic markers (Appendix Fig S5). We attribute their placement to various sources of technical noise. In any case, the amount of cells near the origin is reasonably small in an experimental data setting as can been seen in Appendix Fig S6.

In summary, these results suggest that the cycling of each individual cell describes a circular motion in transcriptome space, and due to cell‐to‐cell variability, the collection of all trajectories describes an annulus‐shaped manifold in two dimensions, or a hollow cylinder when considering three dimensions. That suggests two design principles: (a) low dimensionality—only two dimensions of the high‐dimensional gene expression space are used for the cell cycle (in fact the lowest number possible) and (b) circularity—the trajectory is close to the simplest and smoothest possible shape.

### Verifying that the circle represents the cell cycle

To verify that the two‐dimensional annulus does in fact represent the cell cycle from a functional point of view, we investigated a number of characteristics starting with Gene Ontology (GO) terms relating genes to function (Eden *et al*, 2007; Eden *et al*, 2009). We found that a GO term analysis shows clear dominance of the cell cycle in the first three PCs (Appendix Table S2). However, only the two dynamical components DC1 and DC2 that create the annulus are heavily involved in the cell cycle (Appendix Table S2), while the third dimension (DC3, parallel to the cylinder axis) does not contain any cell cycle‐related GO terms (Appendix Table S2). This supports the conclusion that we isolated the cell cycle into two dimensions by a simple rotation. In agreement with this result, we found that DC3 is almost devoid of clustering with respect to the cell cycle phases (cluster score in Fig 1D), as is also the case for all additional dimensions of PC and DC space.

Due to the simplicity of the shape of the cell cycle trajectories, ordering the cells by their angle in a clockwise motion around the origin of the DC1‐DC2 plane corresponds to the temporal order of the cell’s progression through the cell cycle. This pseudotime order is obtained at no extra cost and represents a simplification of previous methods aiming to extract pseudotime of the cell cycle from asynchronous scRNA‐seq data (Leng *et al.*, 2015; Liu *et al.*, 2017b).

Since mathematical models of the cell cycle have contributed significantly to our mechanistic understanding (Csikász‐Nagy *et al*, 2006; Gérard & Goldbeter, 2009), we investigated whether known phase relationships are reflected in our resulting time courses of individual genes. In Fig 1C, we show genes considered to be part of the “backbone” of the cell cycle (Gérard & Goldbeter, 2011). The cell cycle oscillation is driven by the four main cyclin/CDK complexes: cyclin D/CDK4‐6, cyclin E/CDK2, cyclin A/CDK2, and cyclin B/CDK1. In the skeleton model of Gérard and Goldbeter (2011), constant levels of the cyclin D/CDK4‐6 complex are observed whereas we observe an expression maximum of CCND3 and CDK4 at the beginning/middle of S phase. However, the amplitude of the oscillation of these two genes is noticeably smaller than the one of other genes shown. Adding to this is the observation that the complex does peak during the start of S phase in the extended model by Gérard and Goldbeter (2009). The second complex activated is associated with the two genes CCNE2 and CDK2, responsible for progression through the G1‐S checkpoint (Morgan, 2006). As expected, we observe that both genes have their peak expression right after the point where we suspect the G1‐S transition to happen. Similar to Gérard and Goldbeter, the next cyclin‐associated gene to peak in our data is CCNA2 during the S‐G2 transition, confirming our expectations from literature as its role is to guide the cell through this checkpoint (Morgan, 2006). Lastly, CCNB1, which is part of the mitosis‐promoting factor and responsible for pushing the cell into and through mitosis (Morgan, 2006), has its highest expression at the G2‐M transition just as modeled by Gérard and Goldbeter. Interestingly, we observe that the associated cyclin‐dependent kinase CDK1 is already expressed at the S‐G2 transition.

Overall, the characteristic phase relationships defined in mathematical models are confirmed by our experimental data and analysis. Time courses for other highly variable genes in our dataset also strongly overlap with *Cyclebase* (Santos *et al*, 2015), further confirming that the annulus and the implied temporal order of cells correspond to the cell cycle.

We gauged to what degree principal components and dynamical components are associated with the cell cycle in Fig 1D and E. The marker score (Materials and Methods) in Fig 1D measures the amount which cell cycle‐related genes contribute to particular components. A large cluster score (Materials and Methods) indicates that a particular component strongly separates cells according to their cell cycle phase, which suggests that this component plays a role in the cell cycle. Both scores show that in PC space the cell cycle is heavily influencing the first three components, while in DC space the third component is indistinguishable from higher dimensions. Only the first two DCs are dominated by cell cycle effects.

We took the marker score further and decomposed the variation of the data within each component into different sources (Materials and Methods). Choosing a specific set of genes (such as cell cycle markers), we can distinguish between variations caused by this specific gene set, variations caused by the set of remaining genes and effects caused by the interaction of these two sets. The cell cycle marker genes cause changes in the remaining genes by this interaction, which we quantify by the implied variance. In Fig 1E, we observe that variation of the cell cycle marker gene set (red) and the implied variance (green) accounts for the majority of data variation in the first two DCs. The portion of unexplained variation (not caused by the cell cycle marker gene set) is roughly 80% for DC3. This again clearly indicates that DC3 is not cell cycle dependent, while variation in PC3 is dominated by cell cycle marker effects. Additionally, the portion of unexplained variation in all first three PCs is around 40% suggesting that there are noticeable other effects contained in these components apart from the cell cycle. In summary, the rotation in PC space substantially improves cell cycle identification and separation compared to just taking the first two PCs.

The UMI count per cell should drop to one half upon cell division. Observing such a drop during M phase would be an additional confirmation for our data and the temporal order established by the algorithm when averaged over cells. We divided the cycle into bins with equal cell numbers in order to investigate the development of total UMI counts per cell. A sharp drop of average total UMI counts per cell by approximately a factor 1/2 occurs between the last and first bin of the cycle, at the overlap of G2.M and M.G1 cells (Fig 1F), where cell division happens. That confirms our algorithm and data. Measurements with other cell populations (Appendix Fig S1–S4D) and other choices of bin sizes exhibit a similar drop of total UMI counts.

**Figure EV3:**
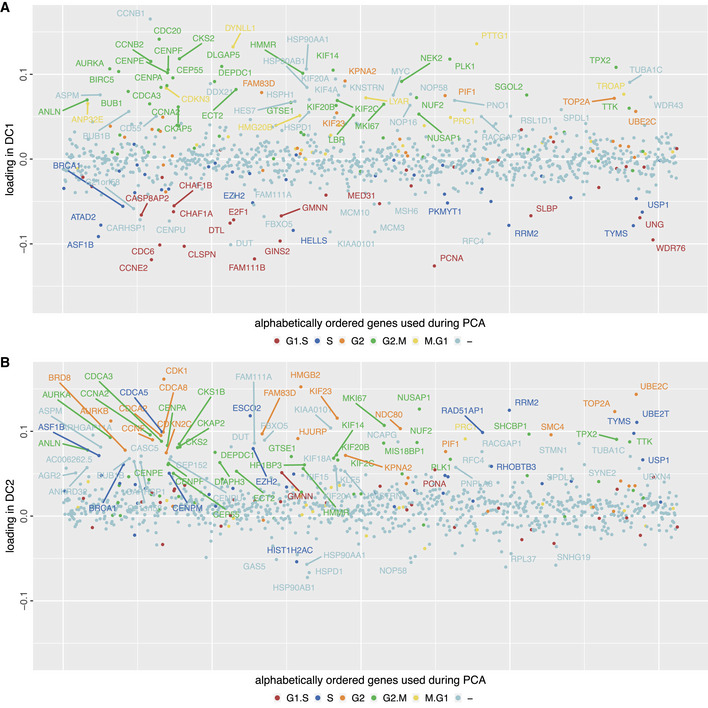
Genes associated with dynamical components mostly correspond to known oscillating genes Weights of genes that span DC1. Colors indicate if a gene is known to be oscillating from Whitfield *et al* (2002). Negative values (corresponding to the left part of the x‐axis of Fig 1B) are mostly associated with G1‐S and S while positive values (right part of x‐axis in Fig 1B) correspond to M phase.Weights of genes that span DC2. Positive values are associated with the transition S‐G2 and M phase. Very few genes have significant negative weights for DC2. Within our cell cycle from Fig 1B, the lower part of the y‐axis corresponds to G1 phase. Thus, this plot confirms that almost no variable genes are active during G1 phase making it difficult to classify cycling cells into G1 because of the lack of marker genes.

The fact that we find the cell cycle in a two‐dimensional annulus in transcriptome space suggests that there are essentially two independent groups of genes, the interaction of which drives the cell cycle (Arnold, 1992). Due to the linearity of our algorithm, the dynamical components DC1 and DC2 are one of several possible representations of the sets of genes for these two groups (Fig EV3). Together, they comprise 266 genes with significant weights. 39 of these genes are found across all three cell types investigated (HeLa, HEK, 3T3), most of which are well known to be cell cycle‐related. The number of joint cell cycle genes is in good agreement with other studies comparing multiple cell types (Dominguez *et al*, 2016; Giotti *et al*, 2017). The well‐known cyclin network provides one of the interactions between DC1 and DC2. The representation of the cyclins in DC1 and DC2 is in agreement with their biological function (described in more detail in Box 1). Hence, our methods provide a basis for extended mechanistic studies.

DC1 and DC2 are only one of many possible partitions of the variable genes into the two groups driving the cycle dynamics. Rotation of the DC‐coordinate system around DC3 yields other possible partitions in terms of dimensions of the DC space. In general, DC1 and DC2 are not disjoin sets of genes, but rather genes may be an element of both of them. Mechanistic studies might suggest other distributions of genes on the two groups, which are compatible with the trajectories in gene expression space, and provide insight into the function and role of specific genes.

The cell’s response to perturbations is described by the stability of the annular manifold—the more stable the manifold is, the faster the cell returns to the unperturbed state. A manifold that is dynamically stable is called an attractor. Mojtahedi *et al* (2016) have shown that the ratio of average gene‐to‐gene correlation to average cell‐to‐cell correlation increases with decreasing stability of attractors in transcriptome space. Based on this measure, we found that the stability of the attractor throughout the cell cycle does not change significantly (Appendix Fig S7), i.e., the cell types we investigated (HeLa, HEK, 3T3) do not display time points where they are more vulnerable to perturbations.

### Inferring trajectories with RNA velocity

Our analysis so far has mapped out the sub‐volume of the transcriptome space within which cell cycle dynamics happen as a cloud of data points each from a different cell. This analysis does not reveal the shape of the individual trajectories from which these data points are sampled. Within the data cloud, cells might run on a simple circle or follow a more complicated trajectory (i.e. spiraling around a torus; Box Fig 1). Identifying trajectories requires not only the position of individual cells but also information on the direction of their motion. Since sequencing data contain information about nascent and mature mRNA, transcriptome changes of single cells can be approximately calculated. This has been termed RNA velocity (La Manno *et al*, 2018).

**Figure 2:**
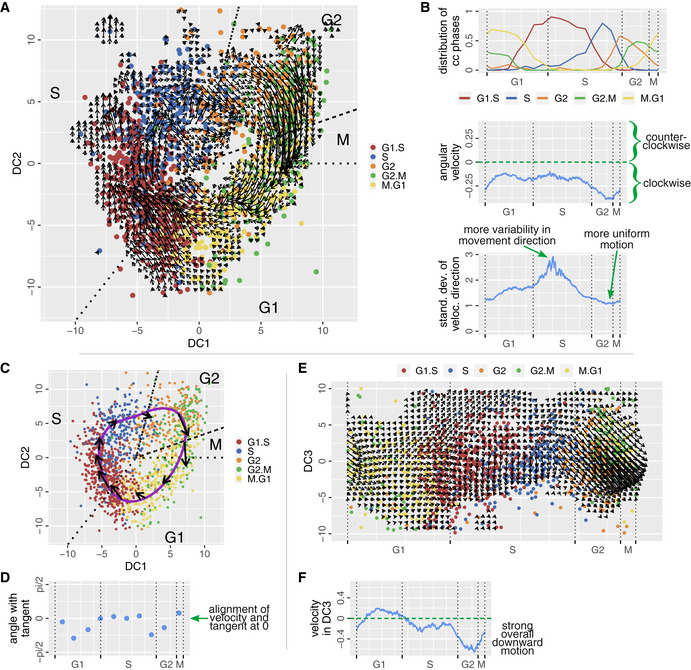
RNA velocity confirms a cyclic motion of the cells and indicates a drift in the third dimension RNA velocity for each cell is calculated (La Manno *et al.*, 2018). We overlay the DC plot with a 50 × 50 grid and assign a weighted average of velocities of surrounding cells with the help of a Gaussian kernel to each grid point (La Manno *et al.*, 2018). The velocity arrow is attached to the position for which the velocity has been calculated. The arrowhead points to the future state. Arrows shorter than 0.1 are not shown.(top) The clusters of cell cycle phases follow the known order G1‐S‐G2‐M‐G1 according to their distribution along pseudotime. (middle) The angular velocity is negative throughout the cycle, indicating continuous clockwise motion. (bottom) We calculate the standard deviation of neighboring velocity arrows. Larger standard deviation indicates more variability in movement direction while lower values suggest more uniform motion.A spline (purple) is placed along the cell cycle approximating an average cell trajectory through the data. We divide the trajectory into 10 angle intervals, each containing the same amount of cells. For each interval, the average RNA velocity is calculated and plotted onto the average trajectory as a black arrow. The velocities are approximately tangential to the average trajectory.We calculate the angular difference between the average RNA velocity and the actual tangent to the trajectory in panel (C). A difference of 0° means perfect alignment of tangent and RNA velocity.RNA velocities in the side of the cylinder. The cylinder has been cut open at the angular coordinate of the M‐G1 transition. The third rotated principal component (DC3) is parallel to the cylinder axis. We do not observe an oscillating motion in the cylinder side. Arrows shorter than 0.4 are not shown.The undulating motion seen in this surface corresponds to a net downward drift with each completion of the cell cycle but not a periodic motion. This indicates that the third dimension does not play a part in the description of the cell cycle.

RNA velocity plotted onto the cell cycle reflects the expected order of cell cycle phase clusters and suggests that the attractor is formed by many circles (Fig 2A). More complicated motion is not supported by the data. The motion of cells is most coherent during G2 and M phase and least directed during S phase (Fig 2B). This points toward a tighter regulation of gene expression during M phase forcing cells through a gene expression tunnel. Cells appear to be more variable in their gene expression when progressing through S phase (Fig 2B).

The arrows in Fig 2A represent the direction of motion of individual cells but do not outline a complete trajectory. We can obtain a complete trajectory not for individual cells but as an average position in each angle bin. If our cell state data and RNA velocity data are consistent, the average RNA velocity should be tangential to the average trajectory. In Fig 2C and D, we observe that the average RNA velocities are indeed mostly tangential when plotted onto the average trajectory (see also Appendix Fig S1–S4F). This strongly suggests that within the two‐dimensional projection of the attractor, single cells do in fact move on a simple circle, and that the direction of motion is determined by transcription.

We also characterized the motion of cells in the direction of the cylinder axis. During G2 and M phase, there is a clear downward motion in the direction of the cylinder axis (Fig 2E). There is motion in the opposite direction during G1 phase, but with smaller speed than the G2 and M phase motion (Fig 2F). Hence, each time cells pass through the cycle, they on average move a little downward but do not return. This motion is a drift parallel to the cylinder axis, which is not periodic and much slower than the motion on the cycle.

An inspection of the GO terms associated with the cylinder axis suggests that response to environmental changes (e.g. change of nutrients) and changes of the epigenetic state dominate the processes that cause the motion parallel to the cylinder axis (Appendix Table S2). The quality of the separation of cell state dynamics into cell cycle (DC1, DC2) and slower processes (DC3) depends on the depths of the sequencing data. Data sets with lower depths (i.e. data set 2, data set 3—see Appendix Table S1) do not always show the slow net drift parallel to the cylinder axis but rather some periodic undulations in this direction are seen as well. In those cases, the data sets also exhibit some remaining cell cycle GO terms in the cylinder axis direction, suggesting that the complete functional isolation of the cell cycle requires sufficient sequencing depth.

**Figure 3:**
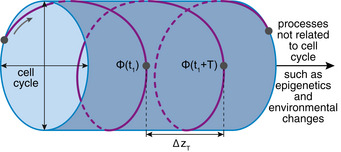
Helical motion of a single cell in transcriptome space Gene expression changes due to cell cycle can be simplified to a two‐dimensional circle by viewing the cylinder from the front or back. Additional cellular processes, suggested by GO terms to correspond to epigenetics and environmental changes, cause a helical motion around a hollow cylinder in transcriptome space. During one cell cycle period of time T, the cell moves parallel to the cylinder axis by $\Delta z_{T}$.

In summary, the RNA velocity confirms our assumption that the cell cycle of individual cells is well approximated by a circle and demonstrates that our analysis can separate fast cyclic motion from slow drift, if a sufficient level of detail is achieved in the data. A simultaneous downward motion in the direction of the cylinder axis transforms the trajectories from circular to helical motion on a hollow cylinder in transcriptome space (Fig 3).

Based on the distribution of cells along the cylinder axis, we divided the cylinder into three parts and analyzed the cycle separately in the bottom, middle, and top range of the cylinder height. The average trajectories in all three ranges are very similar (Appendix Fig S8). We also did not observe clustering of cells with respect to cell cycle phases in the direction of the cylinder axis.

**Figure EV4:**
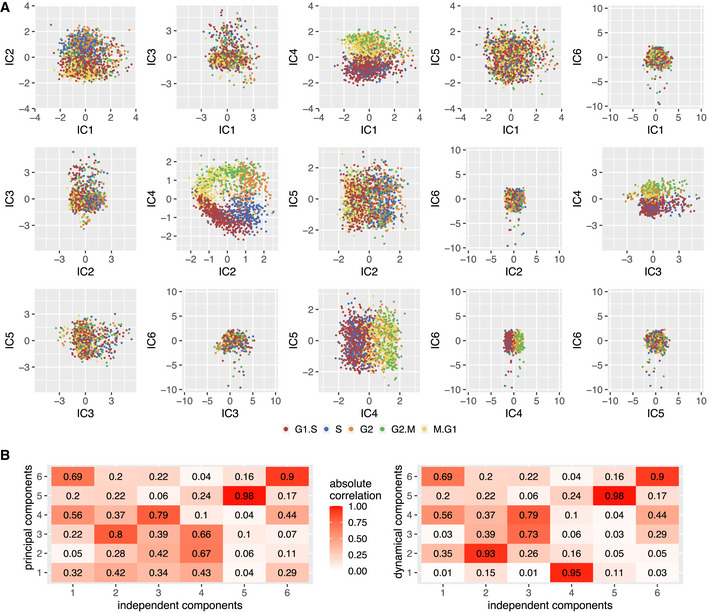
Independent component analysis suggests two independent sources of the cell cycle which are highly correlated to DC1 and DC2 Pairwise projections of independent components IC1 to IC6. In this run of ICA, the two components representing the cell cycle appear to be IC2 and IC4.Correlation of independent components with principal components (left) and dynamical components (right). We observe strong correlation of IC2 and IC4 to DC2 and DC1, respectively. DC1 and DC2 show no strong correlation to other than these two components. Before rotation, PC1 to PC3 exhibit multiple strong correlations to ICs. The correlations of PC4‐PC6 to the ICs are the same as DC4‐DC6 to the ICs since only PC1‐PC3 are involved in the rotation performed by Revelio and thus PC4 and all higher order PCs are equal to their DC counterpart.

Independent component analysis (ICA) aims to separate mixed signals into statistically independent or maximally independent sources. We performed an ICA on our data and found that two of the independent components (ICs) span a plane portraying the cell cycle (Fig EV4, Materials and Methods). It turns out that DC1 and DC2 are each highly correlated with one of these ICs but not significantly to any of the others and vice versa (Fig EV4). Hence, the ICA suggests that DC1 and DC2 are very close to being statistically independent from all other sources (components). Thus, cells appear to be capable of progressing through the cycle independent of influences from gene expression of cellular processes not represented in DC1 and DC2, such as environmental conditions and epigenetic state, which are represented by the direction of the cylinder axis.

### Removal of cell cycle effects from the data

Since the cell cycle is known to convolute other biological signals of interest, multiple methods (Buettner *et al.*, 2015; Barron & Li, 2016; Butler *et al.*, 2018) for the removal of cell cycle effects from scRNA‐seq data have been developed. The removal of the cell cycle from data sets is a straightforward operation with our approach, since we have distilled the cycle into DC1 and DC2 already.

**Figure 4:**
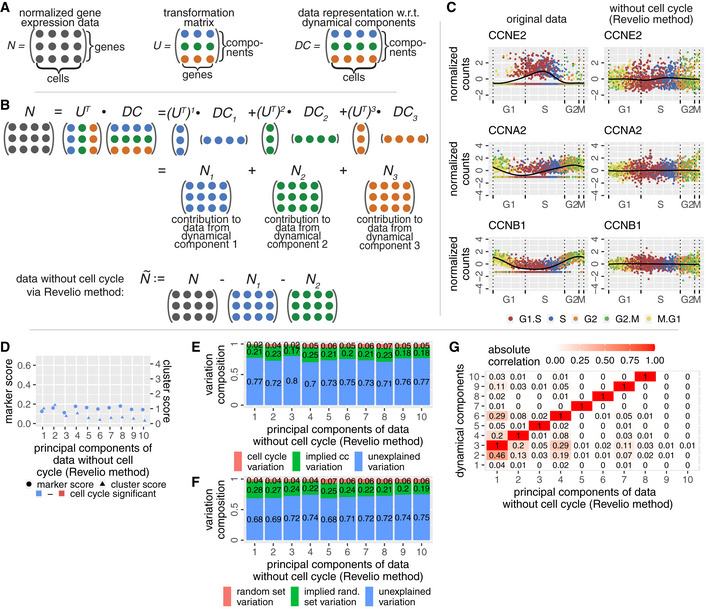
Removing the cell cycle from the data via the Revelio method eliminates known cell cycle signals and keeps additional data intact AThe three main matrices involved in the removal of cell cycle from the data: The normalized gene expression data N (left), the transformation matrix U (middle) and the data representation with respect to dynamical components DC (right). These matrices are related via the equation $DC=U\cdot N\Leftrightarrow N=U^{T}\cdot DC$ (since U is an orthogonal matrix, see Materials and Methods). ${\left(U^{T}\right)}^{i}$ denotes the ith column of $U^{T}$.BThe normalized data are a sum of the contributions of the dynamical components (illustrated for a toy example with three components). By a simple subtraction of the contributions from DC1 and DC2, we remove the cell cycle from the data N and obtain $\tilde{N}$.CComparison of the time courses of three genes associated with cyclin E, A, and B for original data (left) and data processed with Revelio (right).D–GCharacteristics of the data processed with Revelio. (D) Marker and cluster scores exhibit small values (scale is the same as in Fig 1D). (E) The variation decomposition with respect to cell cycle genes is not distinguishable from the variation decomposition with respect to a random gene set (shown in panel F). (F) The variation decomposition with respect to a random gene set. (G) The pairwise correlations between the DCs from original data and the PCs obtained from the data processed with Revelio show a clear one‐to‐one relation. The diagonal with correlation 1 is shifted by 2 dimensions which corresponds to the removal of DC1 and DC2. The high correlation shows that Revelio does not modify components not related to the cell cycle.

The transformation from normalized gene expression space to DC space is done by a linear operator—the rotation matrix *R*. Rotation matrices can be easily inverted by transposing them (Materials and Methods). This enables us to isolate the contribution of an individual dynamical component on the normalized gene expression data. Fig 4B explains that simple products of *R^T^* and the DCs quantify it. Since DC1 and DC2 represent the cell cycle, we simply need to subtract the contributions of these two components from the normalized gene expression data to obtain data without cell cycle effects.

In Fig 4C, we observe that the oscillatory behavior of the genes associated with cyclins E, A, and B is removed after processing with Revelio. Using some of the measures introduced in Fig 1, we see that none of the principal components of the data processed with Revelio suggest any involvement in the cell cycle (Fig 4D). Similarly, the variation decomposition of the new components with respect to cell cycle marker genes (Fig 4E) is almost indistinguishable from a decomposition with respect to a random gene set of the same size (Fig 4F). A noticeable advantage of our approach is the fact that additional biological information in the rest of the data is accurately preserved as seen in Fig 4G. Each newly determined principal component of the data without cell cycle can be clearly correlated with exactly one dynamical component of the original data. This is in contrast to previous methods aiming at removing cell cycle effects from the data (Appendix Fig S9).

Hence, the processing of the data with Revelio leads to the precise removal of specifically those biological functions which were isolated into the first two dynamical components. Information about gene expression of additional processes is not lost. However, the quality and precision of the removal are highly correlated with the quality of cell cycle isolation.

## Discussion

Our results offer a characterization of the transcriptome dynamics of the cell cycle, including a simple method to order unsynchronized cells in time and the ability to accurately remove cell cycle effects from the data. Our analysis benefits from the relatively high depth of our Drop‐seq data. Interestingly, recent data based on sequential single‐molecule FISH (which has much higher RNA detection efficiency than single‐cell RNA‐seq) produced data that are in accordance with our findings (Xia *et al*, 2019).

**Figure EV5:**
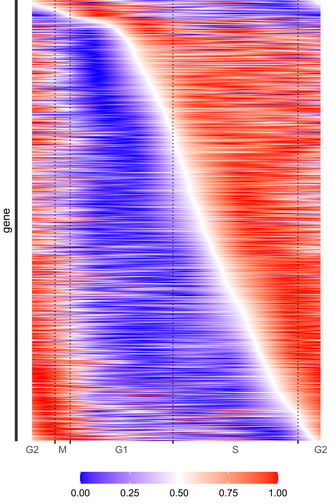
The distribution of transcription initiation of variable genes is constant from mid G1 to mid G2 phase We normalize the time courses of the variable genes to the interval 0.1 $\left(\frac{\text{signal}‐\text{minimum}}{\text{maximum}‐\text{minimum}}\right)$ and order by the time when 0.5 is crossed from below (white line). The slope of the white line reports the rate of transcription onsets per unit time. The steeper the slope, the higher is the rate. We see that this rate is almost constant from the middle of G1 to the middle of G2. It decreases by about a factor 5 between the middle of G2 to the middle of G1 including M phase and cell division. Hence, about 93% of the variable genes have their transcription onset almost equally distribute over 70% of the cycle and 7% start with a lower rate within 30% of the cycle. The rate of onsets is almost constant within both sections. This supports our optimality criterion of avoidance of sudden changes, since we see a decrease from mid G2 to mid G1 phase.

Our analysis of cell cycle topology is based on analytical methods that are linear and therefore preserve the geometry of the trajectory in gene expression space. These geometric properties of the trajectory are directly linked to transcriptional regulation. The circular shape of the cell cycle trajectory effectively minimizes curvature. High curvature of the trajectory in transcriptome space would indicate large acceleration of gene expression, achieved by starting or terminating transcription of genes. Such changes generally entail a large regulatory effort for the cell: Signaling pathways have to be activated, chromatin rearranged, transcription factors, cofactors and activators recruited, enhancers and promoters must interact properly, and RNA polymerases bound (Voss & Hager, 2013). Due to this large regulatory effort, switches in transcriptional programs are error‐prone which can be disadvantageous for the cell. The shape of the trajectory shows that the cell cycle has evolved to avoid these efforts for many genes at the same time and thus minimizes the likelihood of errors due to gradual rather than rapid changes. The distribution of transcription initiation time points of the variable genes further supports this conclusion (Fig EV5). The existence of checkpoints and different cell cycle phases supports the expectation of the concurrent onset of expression of large groups of genes at the beginning of phases. However, the time points of transcription initiation are uniformly distributed both between middle of G2 and early G1 and in the other part of the cycle (Fig EV5). Additionally, the simple cycle is the shape of the trajectory guaranteeing that each gene is up‐ and downregulated only once during the cell cycle. Since we obtain this shape in different cell types, it suggests a universal design principle of the cell cycle.

The linearity of our algorithm is in contrast to non‐linear analysis and visualization methods (k‐nearest neighbors UMAP, t‐SNE), which can be used to flatten more complex manifolds onto a two‐dimensional space. It is generally accepted that single‐cell transcriptomic profiles characterize an expression manifold embedded in the expression space of all genes. Our work shows that, in our setting, the cell cycle is an independent, two‐dimensional manifold within the expression manifold. This begs the question whether the remaining expression manifold can be reduced into further independent submanifolds. Finally, we note that if cells have evolved optimality principles to traverse the cell cycle (as we have shown here) it is tempting to speculate that similar optimality principles of gene expression trajectories may have evolved for a large variety of biological systems—in essence for any developmental or cellular differentiation process. Our method and conceptual framework may be useful to discover these principles.

## Materials and Methods

### Filtering and cell cycle phase assignment

We use computational methods to identify the phase of the cell cycle a specific cell was in at the moment of measurement. The analysis is based on the principles described in Macosko *et al*. (2015) where marker genes for different time points throughout the cell cycle are utilized to assign cells to their cell cycle time point.

We first filter the *m*‐by‐*n* (genes‐by‐cells) digital gene expression matrix *S* to ensure every gene is expressed in at least 5 cells and every cell included in the analysis expresses at least 500 genes. We then normalize each column of *S* by the total amount of UMIs $\theta_{j}$ within the *j*‐th cell and scale by a factor *κ* where $\kappa ={\text{median}}_{j}\left(\theta_{j}\right)$ (the median of all total UMI counts) to obtain scaled counts (Butler *et al.*, 2018). This, we call scaled fraction matrix *SF*:

(1) SF:=κ·S·T

where

(2) $$T=\left(\begin{array}{cccc} \frac{1}{\theta_{1}} & 0 & \cdots & 0\\ 0 & \frac{1}{\theta_{2}} & & \vdots \\ \vdots & & \ddots & 0\\ 0 & \cdots & 0 & \frac{1}{\theta_{n}} \end{array}\right)\;\;\text{and}\;\;\theta_{j}=\sum_{i=1}^{m}s_{\mathrm{ij}}.$$

The entries in SF are referred to as scaled counts. They are displayed in Fig 1C for specific genes. Next, we take the logarithm of SF (Butler *et al.*, 2018). This, we refer to as the logarithmic fraction matrix LF:

(3) $$LF:=\log_{e}\left(SF+1\right)$$

According to Whitfield *et al* (2002), five different cell cycle time points (G1.S, S, G2, G2.M, and M.G1) are each characterized by specific lists of genes, which are typically highly expressed at the corresponding cell cycle phase. These are our five marker gene lists, $\left\{g_{1}^{\eta },\ldots ,g_{m_{\eta }}^{\eta }\right\}=:b^{\eta }$ for $\eta \in \left\{1,2,3,4,5\right\}\hat{=}\left\{G1.S,S,G2,G2.M,M.G1\right\}$, specifying five buckets $b^{\eta }$. Genes not appearing in our data set are discarded from this gene list.

The average expression pattern $\xi_{\eta }$ of each cell *j* w.r.t. each bucket is defined as the vector whose *j*‐th entry is given by

(4) $${\left(\xi_{\eta }\right)}_{j}:=\frac{1}{m_{\eta }}\sum_{g_{i}^{\eta }\in b^{\eta }}{\left(\mathrm{LF}\right)}_{g_{i}^{\eta },j}$$

For each row of *LF* that corresponds to a gene $g_{i}^{\eta }$ contained in bucket *b* ^η^, we now calculate

(5) $$c_{i}^{\eta }:=\text{cor}\left({\left(\mathrm{LF}\right)}_{g_{i}^{\eta },},\xi_{\eta }\right),$$

providing information on how well the expression of a single marker gene corresponds to the average expression of its bucket (Macosko *et al.*, 2015). We discard all genes $g_{i}^{\eta }$ from our buckets for which $c_{i}^{\eta }<0.2$ as they are deemed to behave differently than other genes within the bucket and thus do not contribute to inferring cell cycle phases (Macosko *et al.*, 2015). This yields the buckets $b^{\sim }$ for $\eta \in \left\{1,2,3,4,5\right\}$.

The phase assignment score for cell *j* for phase *η* is given by

(6) $$a_{\eta ,j}:=\frac{1}{{\tilde{m}}_{\eta }}\sum_{g_{i}^{\eta }\in \overset{\sim \eta }{b}}{\left(\mathrm{LF}\right)}_{g_{i}^{\eta },j},$$

yielding the 5‐by‐*n* matrix $A=\left(a_{\eta ,j}\right)$ (Macosko *et al.*, 2015). *A* is normalized w.r.t. rows and columns which transforms *A* into a matrix of z‐scores (Macosko *et al.*, 2015). For each cell *j*, we calculate $q_{j}:=\max_{\eta }\left\{a_{\eta ,j}\right\}$ and we declare phase *η*, for which $q_{j}=a_{\eta ,j}$ holds, to be the cell cycle phase $\eta^{j}$ in which cell *j* is currently located.

We apply two filtering steps. Firstly, we define ${\tilde{q}}_{j}:=\max_{\eta \;\text{s.t.}\;a_{\eta ,j}<q_{j}}\left\{a_{\eta ,j}\right\}$ which gives us the second highest phase assignment score for each cell and the associated phase ${\tilde{\eta }}^{j}$. We discard cells for which $4>\left|\eta^{j}‐{\tilde{\eta }}^{j}\right|>1$ and ${\tilde{q}}_{j}>0.3$. The first condition indicates that the associated phases $\eta^{j}$ and ${\tilde{\eta }}^{j}$ are not neighboring phases while the second condition states that the second highest phase assignment score is significant. These types of cells are suspected doublets as their gene expression peaks in two distant cell cycle phases.

Secondly, we discard cells for which $q_{j}<0.75$ as these cells appear to not exhibit sufficient information for a cell cycle phase assignment.

The data are then again cleaned making sure every gene is expressed in at least 5 cells and every cell expresses at least 500 genes.

In case of data from multiple experiments, the cell cycle phase assignment is done for each experiment individually to avoid dominance of batch effects on the z‐scores of the cell cycle phase assignment.

### Variable genes

In order to investigate variability within our data set without incorporating information on oscillating genes during the cell cycle from the literature, we obtain variable genes according to the principles from the R package Seurat (Butler *et al.*, 2018):

We calculate (Butler *et al*, 2018) the mean expression $\zeta_{i}$ of each gene *i* via

(7) $$\zeta_{i}:=\log_{e}\left(\frac{1}{n}\sum_{j=1}^{n}\exp\left({\left(\mathrm{LF}\right)}_{\mathrm{ij}}\right)\right).$$

The dispersion *d*_i_ of a gene *i* is calculated (Butler *et al.*, 2018) by taking

(8) $$d_{i}:=\log_{e}\left[\frac{\frac{1}{n}\sum_{j=1}^{n}{\left[\exp\left({\left(\mathrm{LF}\right)}_{\mathrm{ij}}\right)‐1‐\frac{1}{n}\sum_{l=1}^{n}\left(\exp\left({\left(\mathrm{LF}\right)}_{\mathrm{il}}\right)‐1\right)\right]}^{2}}{\frac{1}{n}\sum_{j=1}^{n}\left[\exp\left({\left(\mathrm{LF}\right)}_{\mathrm{ij}}\right)‐1\right]}\right].$$

We then compute $c_{\min,\zeta }:=\min_{i}\zeta_{i}$, $c_{\max,\zeta }:=\max_{i}\zeta_{i}$ and the step size $\Delta x:=\frac{c_{\max,\zeta }‐c_{\min,\zeta }}{20}$. Next, we define 20 buckets ${\hat{b}}^{\delta }$ for $\delta \in \left\{1,...,20\right\}$ such that the *i*‐th gene $g_{i}\in {\hat{b}}^{\delta }$ iff

$c_{\min,\zeta }+\left(\delta ‐1\right)\cdot \Delta x\leq \zeta_{i}<c_{\min,\zeta }+\delta \cdot \Delta x$ (Butler *et al.*, 2018).

For a specific $g_{i}\in {\hat{b}}^{\delta }$, we normalize the dispersion (Butler *et al.*, 2018) $d_{i}$ according to all genes within the same bucket ${\hat{b}}^{\delta }$:

(9) $${\tilde{d}}_{i}:=\frac{d_{i}‐\frac{1}{\#{\hat{b}}^{\delta }}\sum_{\;j\text{s.t.}\;g_{j}\in {\hat{b}}^{\delta }}d_{j}}{\sqrt{\frac{1}{\#{\hat{b}}^{\delta }}\sum_{\;j\text{s.t.}\;g_{j}\in {\hat{b}}^{\delta }}{\left[d_{j}‐\frac{1}{\#{\hat{b}}^{\delta }}\sum_{\;l\text{s.t.}\;g_{l}\in {\hat{b}}^{\delta }}d_{l}\right]}^{2}}}.$$

Lastly, we define a gene *i* to be variable iff $0.2<\zeta_{i}<4$ and $0.5<{\tilde{d}}_{i}<10$. The collection of these genes is denoted by *VG*.

Similar to the cell cycle phase assignment algorithm, we analyze variable genes for each experiment $ds_{l}$ individually in case the data contains L>1 data sets. There are multiple ways of combining the resulting variable genes of each batch $VG^{ds_{l}}$ into one set of variable genes $VG^{\mathrm{all}}$. We have chosen:

(10) $$VG^{\mathrm{all}}:=\left\{g_{i}|\#\left\{VG^{ds_{l}}∋g_{i}\right\}\geq Q,l\in \left\{1,\ldots ,L\right\},i\in \left\{1,\ldots ,m\right\}\right\},$$

Where

(11) $$Q:=L‐\left\lceil \frac{L‐1}{3}\right\rceil .$$

As an example: For L=2, this yields Q=1 and thus

(12) $$VG^{\mathrm{all}}=VG^{ds_{1}}\cup VG^{ds_{2}}.$$

### Cell cycle marker gene set within variable genes

It will later on be of interest to investigate the effect of cell cycle marker genes on the data. We therefore define one additional gene set $VG^{\mathrm{cc}}\subset \subset G^{\mathrm{all}}$ to be the set of known cell cycle genes in our variable gene set. For $VG^{\mathrm{cc}}$, we take the union of known cyclic genes from Cyclebase (Santos *et al.*, 2015) and Whitfield *et al* (2002) which yields 880 genes. 691 of these are contained in the HeLa data set 1 and 182 of those are part of the variable gene set. So $VG^{\mathrm{cc}}$ consists of these 182 genes.

### Principal component analysis

In order to apply principal component analysis (PCA), we normalize *LF* (equation 3) row‐wise so that genes are normalized across all cells. Additionally, we reduce the data set to the variable genes $VG^{\mathrm{all}}$ (equation 10) giving us the normalized data matrix *N*.

We can write the covariance matrix $\text{Cov}\left(N^{T}\right)$ of the transposed normalized data matrix $N^{T}$ as.

(13) $$\text{Cov}\left(N^{T}\right):=\frac{1}{n‐1}N\cdot N^{T}.$$

Since $\text{Cov}\left(N^{T}\right)$ is a real, symmetric, square matrix, we know there exists a matrix W such that

(14) $$\text{Cov}\left(N^{T}\right)=W^{T}\cdot D\cdot W$$

holds, where *D* is a diagonal matrix with the eigenvalues $\lambda_{1},\ldots ,\lambda_{m}$ of $\text{Cov}\left(N^{T}\right)$ as its diagonal elements and where the rows of $W^{T}$ are the eigenvectors of $\text{Cov}\left(N^{T}\right)$. $W^{T}$ is orthogonal and even orthonormal. An entry $w_{\mathrm{ij}}^{T}$ of $W^{T}$ is called weight (or loading) *i* for gene *j*.

According to PCA, we obtain a representation *P* of our data *N* with respect to principal components (PCs) by defining

(15) $$P:=W^{T}\cdot N,$$

where the rows of P are now uncorrelated to one another. A row vector $p_{k,}$ of P contains the PC scores (or amplitudes) of all cells with respect to PC k for $k\in \left\{1,...,\#VG\right\}=:K$. The pairwise combinations of the first three PC scores for each cell are depicted in (Fig 1A). The representation P of the data N according to PCs will be referred to as PC space.

### Explained variation and significance of principal components

The total variation of the data P in PC space is given by the sum of the eigenvalues $\lambda_{1},\ldots ,\lambda_{m}$ and it is known that $\sum_{i=1}^{m}\lambda_{i}=m$. The variation explained by each PC i is then given by $\frac{\lambda_{i}}{m}$. We note that this is a property of PCA and that normally the variance explained by an individual dimension/component i of a centered data matrix A is given by the sample variance:

(16) $$\text{Var}\left(A_{i}\right)=\frac{1}{n‐1}\cdot \sum_{j=1}^{n}a_{\mathrm{ij}}^{2}$$

where $A_{i}$ is the i‐th row or i‐th dimension of matrix *A*. For PCA, it can be shown that $\text{Var}\left(P_{i}\right)=\lambda_{i}$.

By considering the size of the eigenvalues and corresponding GO term analyses, we suspect that components past PC6 do not correspond to coordinated biological processes but various degrees of noise (both technical and biological such as cell–cell variability). Statistically, we infer this by finding outliers among the eigenvalues with the interquartile range approach: We have quantile Q1≈0.224, quantile Q3≈1.381, and the interquartile range IQR=Q3‐Q1=1.157. $\lambda_{i}$ is called a major outlier if $\lambda_{i}>Q3+3.5\cdot IQR$. With this approach, we find the first 6 eigenvalues to be outliers and call them significant. The variance explained per principal component $i\in \left\{1,\ldots ,6\right\}$ is then given by ${\text{VarExpl(PC}}_{i})=\frac{\lambda_{i}}{\sum_{j=1}^{6}\lambda_{j}}$.

### Cell cycle cluster score

We define a cluster score to judge to what extent a specific PC k for $k\in \left\{1,...,\#VG\right\}$ is influenced by the cell cycle. Let $p_{\mathrm{kj}}$ be the k‐th PC score for cell j. We divide all cells j for $j\in \left\{1,...,n\right\}$ into five clusters ${\bar{b}}^{\eta }$, $\eta \in \left\{1,2,3,4,5\right\}$ according to their computationally inferred cell cycle phase $\eta^{j}$. For a given PC k, we then calculate the mean $\mu_{\eta }^{k}$ of the PC score for each individual cluster of cells ${\bar{b}}^{\eta }$:

(17) $$\mu_{\eta }^{k}:=\frac{1}{\#{\bar{b}}^{\eta }}\sum_{j\in {\bar{b}}^{\eta }}p_{\mathrm{kj}}.$$

We thus obtain for each PC k five different mean values $\mu_{\eta }^{k}$.

Our idea is now that in case PC k is not influenced by the cell cycle, we should find that these five mean PC scores $\mu_{\eta }^{k}$ attain similar values since the clustering ${\bar{b}}^{\eta }$ (which is done w.r.t. cell cycle phases) should have a negligible impact on the mean PC scores of the clusters. If on the other hand a PC k is influenced by the cell cycle, we expect this to be reflected by differing mean values $\mu_{\eta }^{k}$. This behavior can be measured by investigating the standard deviation $\sigma_{k}$ of the five mean values $\mu_{\eta }^{k}$

(18) $$\sigma_{k}:=\sqrt{\frac{1}{5}\sum_{\eta =1}^{5}{\left[\mu_{\eta }^{k}‐\frac{1}{5}\sum_{\tilde{\eta }=1}^{5}\left(\mu_{\tilde{\eta }}^{k}\right)\right]}^{2}}.$$

We define $\sigma_{k}$ to be our cell cycle score. We note that according to our previous assumptions $\sigma_{k}$ is small for a PC k that is not influenced by the cell cycle. The least cell cycle influence we would expect for any PC k with $\sigma_{k}=0$. If we assume that the cell cycle does in fact manifest itself within M≪#VG PCs, then any PC k that is influenced by the cell cycle, should exhibit a significantly higher $\sigma_{k}$ than the majority of PCs.

This is a relative score meaning that we are not assigning meaning to the absolute values $\sigma_{k}$ of the score. Only if we see significantly higher values in some components than the majority can we hypothesize that these components are influenced by the cell cycle.

### Cell cycle marker score

We have investigated a second measure of the cell cycle variance contained in principal components. This time, we consider the weight matrix $W^{T}$ responsible for transforming the normalized data N into the PC space. We note that $W^{T}$ is an orthogonal matrix, meaning $W\cdot W^{T}=I$, where I is the identity matrix. It also holds that $\sum_{j=1}^{m}{\left(W^{T}\right)}_{\mathrm{ij}}^{2}=1$, for all i. Each row i in $W^{T}$ defines the representation of the data N in ${\text{PC}}_{i}$ through the formula $P=W^{T}\cdot N$. An analysis of these weights (or loadings) therefore holds valuable information about which genes (as each weight corresponds to a gene) play the biggest role in the data distribution within each ${\text{PC}}_{i}$.

If there existed a ${\text{PC}}_{i}$ for which all genes would be of equal importance, then $\left|{\left(W^{T}\right)}_{\mathrm{ij}}\right|=\left|{\left(W^{T}\right)}_{\mathrm{ik}}\right|$ for all j,k. Thus, ${\left(W^{T}\right)}_{\mathrm{ij}}^{2}=\frac{1}{m}$ for all j which we call the expected average weight. We will therefore consider all genes j corresponding to weights for which ${\left(W^{T}\right)}_{\mathrm{ij}}^{2}>\frac{1}{m}$ (larger than expected average weight) to be of importance for ${\text{PC}}_{i}$.

The known cyclic genes are contained in $VG^{\text{cc}}$. The degree to which known cyclic genes are responsible for the representation of the data in ${\text{PC}}_{i}$ can be estimated by the score.

(19) $$S_{i}:=\sum_{j\in G_{\mathrm{cc}}}{\left(W^{T}\right)}_{\mathrm{ij}}^{2},$$

which sums up all squared weights corresponding to known cyclic genes. For a component not involved in the cell cycle, we would expect a score $S_{i}\leq \frac{\#VG^{\text{cc}}}{\#VG^{\text{all}}}$ (where the right‐hand side is the summed expected average weight).

### Rotation of three‐dimensional space

We want to rotate the PC space spanned by the first three principal components in order to find a two‐dimensional plane that contains the cell cycle. A rotation of three‐dimensional space may be executed by a series of two‐dimensional rotations with matrices taking the form

(20) $$\begin{array}{c} R_{x}\left(\alpha \right)=\left(\begin{array}{ccc} 1 & 0 & 0\\ 0 & \cos\left(\alpha \right) & ‐\sin\left(\alpha \right)\\ 0 & \sin\left(\alpha \right) & \cos\left(\alpha \right) \end{array}\right),\;R_{y}\left(\beta \right)=\left(\begin{array}{ccc} \cos\left(\beta \right) & 0 & \sin\left(\beta \right)\\ 0 & 1 & 0\\ ‐\sin\left(\beta \right) & 0 & \cos\left(\beta \right) \end{array}\right),\\ R_{z}\left(\gamma \right)=\left(\begin{array}{ccc} \cos\left(\gamma \right) & ‐\sin\left(\gamma \right) & 0\\ \sin\left(\gamma \right) & \cos\left(\gamma \right) & 0\\ 0 & 0 & 1 \end{array}\right). \end{array}$$

for some angles α, β, γ. The resulting rotation is then given by $R\left(\alpha ,\beta ,\gamma \right)=R_{z}\left(\gamma \right)\cdot R_{y}\left(\beta \right)\cdot R_{x}\left(\alpha \right)$. Without loss of generality, we can dismiss one of these rotation matrices as all necessary rotations of the space can be achieved by a combination of two angles. We choose γ=0, yielding $R_{z}=I_{3}$.

More generally, we can rotate a three‐dimensional subspace of a larger vector space (with dimension > 3) by filling out all other dimensions not involved in the rotation by the identity matrix. An example of a ration matrix R of the three‐dimensional subspace spanned by dimension 1, 4, 6 in a 6‐dimensional space:

(21) $$\left(\begin{array}{cccccc} r_{11} & 0 & 0 & r_{12} & 0 & r_{13}\\ 0 & 1 & 0 & 0 & 0 & 0\\ 0 & 0 & 1 & 0 & 0 & 0\\ r_{21} & 0 & 0 & r_{22} & 0 & r_{23}\\ 0 & 0 & 0 & 0 & 1 & 0\\ r_{31} & 0 & 0 & r_{32} & 0 & r_{33} \end{array}\right).$$

Our goal is to find appropriate angles α, β such that the direction vector ω of the axis of the cylinder forming the manifold is in the direction of the third rotated component.

(22) $$R\left(\alpha ,\beta \right)\cdot \omega =\left(\begin{array}{c} 0\\ 0\\ 1 \end{array}\right).$$

Given ω, the entries of the rotation matrices system can be determined like.

(23) $$\sin\left(\alpha \right)=\frac{\omega_{2}}{\omega_{2}^{2}+\omega_{3}^{2}}\cdot \sqrt{1‐\omega_{1}^{2}},$$

$$\cos\left(\alpha \right)=\frac{\omega_{3}}{\omega_{2}^{2}+\omega_{3}^{2}}\cdot \sqrt{1‐\omega_{1}^{2}},$$

$$\sin\left(\beta \right)=‐\omega_{1},$$

$$\cos\left(\beta \right)=\sqrt{1‐\omega_{1}^{2}}$$

### Finding the optimal rotation

We want to rotate the PC space in an unsupervised manner. Our optimization is that after rotation of a three‐dimensional subspace spanned by components $k_{1}$, $k_{2}$, $k_{3}$ for $k_{i}\in \left\{1,...,\#VG^{\mathrm{all}}\right\}$ the cell cycle score $\sigma_{{\tilde{k}}_{3}}$ (equation 18) is minimal in the new third component ${\tilde{k}}_{3}$. This condition derives from considering a triplet of mean PC scores ($\mu_{\eta }^{k_{1}},\mu_{\eta }^{k_{2}},\mu_{\eta }^{k_{3}})$ (equation 17) as a point $\gamma_{\eta }$ within our three‐dimensional subspace for $\eta \in \left\{1,2,3,4,5\right\}$. Each of the five cell cycle time points (G1.S, S, G2, G2.M, and M.G1) yields one such point $\gamma_{\eta }$. We now attempt to place all of these five points $\gamma_{\eta }$ into a single plane. Minimizing the distance of the five points $\gamma_{\eta }$ to that plane is equivalent to minimizing the cell cycle score for the vector orthogonal to the plane. The fact that such a plane exists is non‐trivial. We will refer to the orthogonal vector corresponding to the plane as the viewing axis.

The pair (α,β) defines a solid angle. We do a two‐step optimization. First, we divide the total solid angle of 4π into 10,000 bins of equal size. Utilizing the golden spiral algorithm (also referred to as spherical Fibonacci grid) (Vogel, 1979; Swinbank & Purser, 2006), we generate 10,000 approximately equidistantly spaced points on a unit sphere. Each of these points is a potential viewing axis. For each of them, we calculate the corresponding cell cycle score. The axis $\omega_{i}$, $i\in \left\{1,\ldots ,10,000\right\}$ associated with the lowest cell cycle score is chosen to be optimal.

As a second step, we refine the grid of potential viewing axes in a small neighborhood of $\omega_{i}$ by roughly the factor $\frac{1}{1000}$. Again, we find the viewing axis ${\tilde{\omega }}_{\tilde{t}}$ associated with the lowest cell cycle score and choose this ${\tilde{\omega }}_{\tilde{t}}$ to be the viewing axis that becomes the vector $\left(\begin{array}{c} 0\\ 0\\ 1 \end{array}\right)$ after rotation of the three‐dimensional subspace.

### Generalization to sequence of rotations and selection of cell cycle significant components

So far, we have always assumed that PCA manages to place the cell cycle drivers within the first three dimensions. This is unfortunately only true for sufficiently deep sequenced data sets. We have investigated multiple Drop‐seq data sets from HeLa, HEK, and 3T3 cells where we find significant cell cycle scores for more than three PCs. Therefore, it is necessary that we advance from a single rotation of a three‐dimensional subspace to a sequence of three‐dimensional rotations. We note that combining two rotation matrices $R_{1},R_{2}$ again yields a rotation matrix $R=R_{2}\cdot R_{1}$.

We consider the cell cycle score for the first 100 principal components. We need to judge which of these components are significantly influenced by the cell cycle. This comes down to an outlier detection problem. We would in general expect to obtain most cell cycle scores close to zero with only a handful significantly higher scores, implying cell cycle influence for those few components. We detect outliers via the sample mean and sample standard deviation, restricted to the first 100 components. For the vector ${\vec{\sigma }}_{\bar{K}}:=\left(\sigma_{1},...,\sigma_{100}\right)$ (see equation 18), $\bar{K}:=\left\{1,...,100\right\}\subset \left\{1,\ldots ,\#VG\right\}=K$, we consider the sample standard deviation (SD).

(24) $$SD\left({\vec{\sigma }}_{\bar{K}}\right)=\sqrt{\frac{1}{\#\bar{K}‐1}\cdot \sum_{j\in \bar{K}}\left(\sigma_{j}‐\text{mean}\;\left({\vec{\sigma }}_{\bar{K}}\right)\right)}$$

and define $\sigma_{\bar{k}}$ for $\bar{k}\in \bar{K}$ to be an outlier iff

(25) $$\left|\sigma_{\bar{k}}‐\text{mean}\left({\vec{\sigma }}_{\bar{K}}\right)\right|>2\cdot SD\left({\vec{\sigma }}_{\bar{K}}\right).$$

Let $\overset{\vee }{K}$ be the collection of $\bar{k}$ for which $\sigma_{\bar{k}}$ is an outlier. Then, any PC $\overset{\vee }{k}\in \overset{\vee }{K}\subset \bar{k}$ is considered to be a PC on which the cell cycle has significant influence.

Our goal is to place the cell cycle influence into the first two components. Therefore, the first two components always span the first two dimensions of the three‐dimensional subspace we rotate. The third dimension is spanned by a PC $\overset{\vee }{k}\in \overset{\vee }{k}\backslash \left\{1,2\right\}$.

As an example, we assume our outlier detection found that PC1, PC2, PC3, PC5, and PC8 have significant cell cycle scores. This yields $\overset{\vee }{K}\backslash \left\{1,2\right\}=\left\{3,5,8\right\}$ and implies that we require three subsequent three‐dimensional rotations. The first step is the same as described previously: We select the three‐dimensional space spanned by PC1, PC2, and PC3, we find the optimal viewing axis for this subspace and rotate the data set accordingly by a matrix $R_{1}$. This yields rotated‐PC1, rotated‐PC2, and rotated‐PC3 where the cell cycle score of rotated‐PC3 was minimized and the cell cycle effects exhibited by PC3 previously were ideally included into rotated‐PC1 and rotated‐PC2. In the next step, we select the three‐dimensional subspace spanned by rotated‐PC1, rotated‐PC2, and rotated‐PC5 and find the optimal viewing axis such that the cell cycle score is minimal in rotated‐PC5. We obtain $R_{2}$. Finally, this is repeated with the newly rotated‐PC1, newly rotated‐PC2 and rotated‐PC8 yielding $R_{3}$. In total, we have a sequence of three three‐dimensional rotations $R_{1}$, $R_{2}$, $R_{3}$ which when combined are in fact realized by a single rotation matrix $R=R_{3}\cdot R_{2}\cdot R_{1}$.

We find that with this method, we are able to isolate cell cycle effects into just two dimensions for all data sets investigated (visualized in Fig 1, Appendix Fig S1–S4). The algorithm is not influenced by batch effects and will ignore such effects as long as the relevant cell cycle information is present and contained within the first 100 PCs. We have set the boundary of 100 PCs as we have not yet found any data set that had significant cell cycle scores past the 100th PC. The algorithm can be extended to include as many PCs as desired. Only the detection of outliers has to then be adjusted to account for additional data points influencing the outlier detection algorithm.

In the end, we find a new representation DC of the data N by multiplying a rotation matrix R from the left onto the representation P (equation 15) via.

(26) $$DC:=R\cdot P=R\cdot W^{T}\cdot N=U\cdot N$$

where R is a sparse orthogonal matrix which causes cell cycle effects to be maximized in the first two dimensions and $U:=R\cdot W^{T}$ is again an orthogonal matrix. The representation DC of the data N according to rotated PCs will be referred to as dynamical component space (DC space), and the new components (rotated PCs in the above example) will be referred to as dynamical components (DCs).

### Dynamic components

The dynamics of the cell cycle is the dynamics of the mRNA and protein concentrations of the cell. We restrict our analysis to the mRNA concentrations. Neglecting noise, it can be described by a large system of ordinary differential equations

(27) $$\frac{dX}{dt}=F\left(X,p\right).$$

Here, X denotes the vector of the mRNA concentrations, p a vector of parameter values and t denotes the time variable. The dependence on p captures also cell variability. In general, the time course of X on the manifold can be described by a system of differential equations for abstract variables $A=\left(a_{1},...,a_{M}\right)$ with fewer components $a_{i}$ than the large number m of mRNAs:

(28) $$\frac{dA}{dt}=H\left(A,p\right)$$

The original data are related to the abstract variables by algebraic functions *G.*

(29) $$X\left(t\right)=G\left(a_{1}\left(t,p\right),\ldots ,a_{M}\left(t,p\right)\right).$$

Such a description is useful, if very few $a_{i}$ provide a good approximation of the time course, i.e., M≪m. M is an upper limit for the dimension of the manifold. There is a variety of methods of finding the abstract variables (Haken, 1983; Kuznetsov, 1998). Our results show that the cell cycle dynamics (motion on the manifold) can be represented in good approximation with M=2, described by differential equations for $a_{1}$ and $a_{2}$:

(30) $$\frac{da_{1}}{dt}=H_{1}\left(a_{1},a_{2},p\right),\frac{da_{2}}{dt}=H_{2}\left(a_{1},a_{2},p\right),$$

and a particularly simple function *G*:

(31) $$X\left(t\right)=\sum_{i=1}^{2}a_{i}\left(t,p\right)\cdot E_{i}\left(p\right).$$

$E_{1}$ and $E_{2}$ are sums of principal components and are called dynamical components (DCs). The rotated PC1 and rotated PC2 are one of several possible choices of $E_{1}$ and $E_{2}$. We therefore denote rotated PC1 and rotated PC2 by DC1 and DC2, respectively. Additional dimensions (rotated PC m) are denoted analogously by DC m. Other possible choices for DC1 and DC2 follow from rotated PC1 and rotated PC2 by rotation around the cylinder axis (DC3 direction).

### Cell cycle cluster and marker score for dynamical components

The definitions of the cell cycle cluster and marker scores (see sections “Cell cycle cluster score” and “Cell cycle marker score”) for the dynamical components are completely analogue to the calculations for principal components. The only difference is that instead of the PC scores in P, we consider the scores in DC and instead of the weight matrix W, we consider the weight matrix $U:=R\cdot W^{T}$ responsible for transforming the normalized data N into DC in DC space. The matrix U is again an orthogonal matrix.

### Variation decomposition

We want to assess the cell cycle variance contained in each of the principal and dynamical components. In Buettner *et al* (2015), this was estimated by calculating the variance caused by a gold‐standard set of cell cycle genes and comparing it to the overall variance. We have $VG^{\text{all}}$ the set of variable genes and $VG^{\text{cc}}\subset \subset G^{\text{all}}$ the set of cell cycle genes (see sections “variable genes” and “Cell cycle marker gene set within variable genes”). We take a similar approach as in Buettner *et al.* (2015) and split the data representation P and DC along the cell cycle genes into two representations:

$$P=P^{VG^{\mathrm{cc}}}+P^{VG^{\mathrm{all}}\backslash VG^{\mathrm{cc}}}and\;\;DC=DC^{VG^{\mathrm{cc}}}+DC^{VG^{\mathrm{all}}\backslash VG^{\mathrm{cc}}}$$

which states that the representations P and DC can be divided into a sum where the first source is determined by cyclic genes and the second by all other genes. This is made possible by considering the following “picture”:

$$N=\left(\begin{array}{ccc} & N^{VG^{\text{cc}}} & \\ \ldots \ldots & \ldots \ldots & \cdots \cdots \\ & N^{VG^{\text{all}}\backslash VG^{\text{cc}}} & \end{array}\right)\;\;\text{and}\;\;W=\left(\begin{array}{ccc} & \vdots & \\ W^{VG^{\text{cc}}} & \vdots & W^{VG^{\text{all}}\backslash VG^{\text{cc}}}\\ & \vdots & \end{array}\right).$$

In this representation, we split N and W along the known cyclic genes in the set $VG^{\text{cc}}$. $N^{VG^{\text{cc}}}$ contains all cells but only the cell cycle gene set, while $W^{VG^{\text{cc}}}$ is the part of the rotation matrix affecting all genes in the set $VG^{\text{cc}}$. Then, we define:

$$P^{VG^{\text{cc}}}=W^{VG^{\text{cc}}}\cdot N^{VG^{\text{cc}}},$$

$$P^{VG^{\mathrm{all}}\backslash VG^{\mathrm{cc}}}=W^{VG^{\mathrm{all}}\backslash VG^{\mathrm{cc}}}\cdot N^{VG^{\mathrm{all}}\backslash VG^{\mathrm{cc}}},$$

$$DC^{VG^{\mathrm{cc}}}=R\cdot W^{VG^{\mathrm{cc}}}\cdot N^{VG^{\mathrm{cc}}},$$

$$DC^{VG^{\mathrm{all}}\backslash VG^{\mathrm{cc}}}=R\cdot W^{VG^{\mathrm{all}}\backslash VG^{\mathrm{cc}}}\cdot N^{VG^{\mathrm{all}}\backslash VG^{\mathrm{cc}}}.$$

By basic considerations about calculation rules of matrix multiplication, we can conclude that the equations $P=P^{VG^{\mathrm{cc}}}+P^{VG^{\mathrm{all}}\backslash VG^{\mathrm{cc}}}\text{and}\;\;DC=DC^{VG^{\mathrm{cc}}}+\;\;DC^{VG^{\mathrm{all}}\backslash VG^{\mathrm{cc}}}$ hold.

Without loss of generality, we only consider the matrix P and its decomposition from now on. All considerations also apply to DC. With the help of basic calculation rules of variances, we can decompose the variance into

$$\begin{array}{c} \text{Var}\left(P_{i}\right)=Var\left(P_{i}^{VG^{\mathrm{cc}}}\right)+Var\left(P_{i}^{VG^{\mathrm{all}}\backslash VG^{\mathrm{cc}}}\right)+2\cdot \text{Cov}\left(P_{i}^{VG^{\mathrm{cc}}},P_{i}^{VG^{\mathrm{all}}\backslash VG^{\mathrm{cc}}}\right)\\ =Var\left(P_{i}^{VG^{\mathrm{cc}}}\right)+Var\left(P_{i}^{VG^{\mathrm{all}}\backslash VG^{\mathrm{cc}}}\right)+2\cdot P_{i}^{VG^{\mathrm{cc}}}\cdot {\left(P_{i}^{VG^{\mathrm{all}}\backslash VG^{\mathrm{cc}}}\right)}^{T}, \end{array}$$

for any component i. We now consider $\text{Var}\left(P_{i}^{VG^{\text{cc}}}\right)$ to be the variance directly explained by the cell cycle as we defined $VG^{\text{cc}}$ to contain only known cell cycle genes, $\text{Var}\left(P_{i}^{VG^{\text{all}}\backslash VG^{\text{cc}}}\right)$ is the unexplained variance and the last term describes the confounding effects between the two gene sets $VG^{\text{cc}}$ and $VG^{\text{all}}\backslash VG^{\text{cc}}$. We would expect the last term to be zero if both gene sets were completely independent. This is of course not the case as $VG^{\text{all}}\backslash VG^{\text{cc}}$ will undoubtedly contain genes that are not marked as clear cyclic genes but nevertheless play an indirect role during the cell cycle. We call this “implied cell cycle variance”. If the implied cell cycle variance is positive, we can define portions of variances:

$$\text{cell cycle variance}=\frac{\text{Var}\left(P_{i}^{VG^{\mathrm{cc}}}\right)}{\text{Var}\left(P_{i}\right)},$$

$$\text{implied cell cycle variance}=\frac{2\cdot P_{i}^{VG^{\mathrm{cc}}}\cdot {\left(P_{i}^{VG^{\mathrm{all}}\backslash VG^{\mathrm{cc}}}\right)}^{T}}{\text{Var}\left(P_{i}\right)},$$

$$\text{unexplained variance}=\frac{\text{Var}\left(P_{i}^{VG^{\mathrm{all}}\backslash VG^{\mathrm{cc}}}\right)}{\text{Var}\left(P_{i}\right)}.$$

These considerations hold true analogously for the representation DC. We have investigated the data for the first 10 components in the PC and DC space (Fig 1E). We observe that the PCs with the biggest proportion of cell cycle variation are the ones that Revelio selects for rotation (first three). In the DC space, we observe that we only have 14% and 17%, respectively, of unexplained variation present in DC1 and DC2, whereas in the other components the unexplained variation ranges from 70% to 82%.

As a control, we have randomly sampled 182 genes from the 1031 variable genes and redone the analysis in Appendix Fig S10. We observe in all components a similar distribution as seen for DC3‐DC10 in Fig 1E. Additionally, we note that $1‐\frac{182}{1031}\approx 0.823$ ($VG^{\text{cc}}$ contains 182 genes, $VG^{\text{all}}$ contains 1,031 genes). Considering all components i, we observe that $\left(\text{unexplained variance}+\frac{\text{implied random set variance}}{2}\right)∼N\left(0.823,\;0.00031\right)$, meaning the decomposition of variation of a random gene set likely only depends on the number of genes in each set for a random selection of genes. This suggests that a component exhibiting a similar distribution in its variation decomposition is suggested to be only randomly influenced by the chosen gene set. Hence, DC3‐DC10 are implicated to contain no coordinated effect from known cyclic genes.

### Synchronizing cell cycle to cell division

In order to compare different data sets, we want to find a way to synchronize the obtained cell cycle to a known time point that exists in all data sets. Cell division is present in all cell types we are investigating. Furthermore, we can approximate the moment of cell division by investigating the amount of mRNA transcripts contained within cells along the cell cycle which makes cell division an ideal candidate to align our data sets to.

More specifically, we consider the cell cycle displayed by DC1 and DC2 and divide the data into $\tilde{n}$ bins containing 30 cells each. For each bin, the average total UMI count is calculated providing us with a time course $\left(U_{i}\right),i\in \left\{1,\ldots ,\tilde{n}\right\}$ of average total UMI counts along the cell cycle. We take the minimum and maximum of $\left(U_{i}\right)$ and construct a linear function $h\left(x\right)$ such that $h\left(1\right)=\min_{i}U_{i}$ and $h\left(\tilde{n}\right)=\max_{i}U_{i}$. We now consider all permutations $\pi_{l},l\in \left\{1,...,\tilde{n}\right\}$ of the set $\left\{1,\ldots ,\tilde{n}\right\}$ which periodically shift every element by l‐1. Then, we search for the permutation $\pi_{l}$ which attains the minimum in squared residuals between the time course and the linear function:

(32) $$\min_{\pi_{l}}\left[\sum_{i=1}^{\tilde{n}}{\left(U_{\pi_{l}\left(i\right)}‐h\left(i\right)\right)}^{2}\right].$$

This is a simple but efficient approximation for the bin l at the start of which the cell division is most likely to take place due to the fact that we expect a sudden drop in average total UMI counts between cells about to divide and the ones that just divided. The increase in average UMI counts per cell is in reality not linear but we have seen during analysis of multiple data sets that this approximation is sufficient.

Finally, let $\alpha_{x}$ be the minimal angle a cell attains in polar coordinates within bin l and let $\alpha_{x‐1}$ be the maximum angle of all cells from bin l‐1. We rotate the two‐dimensional cell cycle about an angle $\alpha =‐\frac{\alpha_{x}+\alpha_{x‐1}}{2}$, thereby placing the time point of cell division onto the positive axis of DC1.

### Phase space density and speed along trajectories

Cells were grown asynchronously in vitro. As no synchronization of cell cycle phases was performed, it is reasonable to approximate cells to be uniformly distributed along the time axis of one cell cycle during the experiment. Under this assumption, the time variable in high‐dimensional gene expression space is represented by the phase space density (the density of cells along a trajectory). In areas of significantly higher cell density, we can assume that time passes slower than in areas along the trajectory of low cell density. In case of uniform density, one can conclude that time is progressing linearly when moving along a trajectory with constant speed.

While we do not obtain perfect uniform distribution of our cells (Appendix Fig S11A), the cells in our HeLa data 1 utilizing 1,031 genes during PCA are reasonably well distributed in phase space (sample cumulative distribution in blue, ideal uniform cumulative distribution function for U[0,1] in black). More interestingly, if we consider the same HeLa data but 12,773 genes during PCA, the distribution becomes even closer to uniform (Appendix Fig S11B).

While both of these distribution are statistically not uniform, they are reasonably close to conclude that an additional non‐linear transformation which evens out phase space density will not alter the basic characteristics of the data clouds.

All time course analyses are done by uniformly distributing cells along the cell cycle axis so that progression along time courses shown in Figs 1, 2 and 4 is approximately linearly proportional to progression through actual cell cycle time.

### Incorporating cell cycle phase durations from literature into phase space plots

There are multiple publications on measuring the lengths l of cell cycle phases. For HeLa and 3T3 cell lines, we obtain values for cell cycle phase lengths from Hahn *et al* (2009) and for HEK293 data sets from Cheng & Solomon (2008) These cell cycle phase lengths and their location in our plots are to be understood as rough estimates. We note that we specifically do not observe discrete switches from one phase to another but rather continuous transition between them. The notations of cell cycle phases were created by scientists in order to group processes and facilitate description of such.

Since we have previously defined the time point of cell division within our data, we equate this time point to the transition from M to G1 phase. In the previous section, we have argued that we can relate the density of data points along the cell cycle in transcriptome space to information about actual cell cycle time. We order the cells according to their angle in polar coordinates. From the first cell after cell division, we define the transition G1‐S to take place after x cells where x can be computed from

(33) $$\frac{x}{\#\text{of total cells}}=\frac{l_{G1}}{l_{\text{total}}}.$$

The mean angle of the cells x and x+1 gives us an estimate of where in our phase space the transition G1‐S takes place. We repeat this step for all remaining transitions analogously.

### RNA velocity analysis

La Manno *et al* (2018) introduced RNA velocity as a concept of distinguishing between unspliced and spliced RNA in order to extrapolate cell states to a future time point. We implement the approximation model I from the supplement of La Manno *et al* (2018) into our analysis. Let $s_{\mathrm{ij}}\left(t\right)$ be the number of spliced transcripts of a specific gene i present in a cell j dependent on time t. The assumption under model I is that the time derivative of $s_{\mathrm{ij}}$ is constant

(34) $$\frac{ds_{\mathrm{ij}}}{dt}=v_{\mathrm{ij}}\Rightarrow s_{\mathrm{ij}}\left(t\right)={\left(s_{\mathrm{ij}}\right)}_{0}+v_{\mathrm{ij}}\cdot t.$$

The velocity matrix V is estimated as is shown in the supplement of La Manno *et al.* (2018). We set ${\left({\left(s_{\mathrm{ij}}\right)}_{0}\right)}_{i\in \left\{1,\ldots ,m\right\},j\in \left\{1,\ldots ,n\right\}}=S$ where S is our raw data matrix (see Materials and Methods section "Filtering and cell cycle phase assignment"). We choose t=1 and we obtain the extrapolated state matrix $s_{\text{ex}}$ via

(35) $$s_{\mathrm{ex}}=S\cdot T+V$$

(see equations 2, 34). Next, we transform the data to $LF_{\text{ex}}$ (equation 3) similarly as before.

(36) $$LF_{\mathrm{ex}}=\log_{e}\left(10^{4}\cdot s_{\mathrm{ex}}+1\right).$$

We then normalize $LF_{\text{ex}}$ for each gene across all cells according to the mean and standard deviation of each gene in $LF_{\text{ex}}$ and limit ourselves to the variable genes found during our previous analysis which yields the normalized extrapolated data matrix $N_{\text{ex}}$. Lastly, we transform the data points into the rotated PC space where the first two dimensions represent the cell cycle:

(37) $$DC_{\mathrm{ex}}=R\cdot W^{T}\cdot N_{\mathrm{ex}}$$

(equations 15, 26).

Due to the high noise level in the unspliced data, we have to incorporate a smoothing grid on top of the DC1‐DC2 plot in order to obtain relevant information about the direction of motion of the cells. The calculation of the grid is again done as described in the supplement of La Manno *et al.*(2018), incorporating a Gaussian kernel function

(38) $$K_{\sigma }\left(x_{1},x_{2}\right):=\exp\left(\frac{‐∥x_{1}‐x_{2}^{2}∥}{2\varrho^{2}}\right)$$

and defining the displacement of a grid point $x_{{\text{grid}}_{k}}$ via

(39) $$\Delta x_{{\text{grid}}_{k}}:=\sum_{j}K_{\sigma }\left(x_{{\text{grid}}_{k}},x_{j}\right)\cdot \Delta x_{j}.$$

The smoothing parameter ϱ (equation 38) can be chosen freely (La Manno *et al.*, 2018). We take care to find a balance between smoothing enough to get a reasonable idea of the general motion of the system but at the same time taking care not to eliminate too much noise so that there is room for interpretation of the strength of cyclic motion at different time points during the cell cycle. In Fig 2A, we have chosen ϱ=0.6 (equation 38).

### RNA velocity in the side of the cylinder

In order to show that we isolated cell cycle into just two dimensions, we investigate also the motion of the cells parallel to the cylinder axis. A cylinder can be described by the angle φ and radius r of its base and the height corresponding to the direction of DC3 in our representation of the data.

State changes in φ‐direction are calculated by calculating polar coordinates for our data and the extrapolated data in the DC1‐DC2 plot. The changes in height direction are given by the changes in DC3 between the data and the extrapolated state. Due to high noise levels, we again apply a grid smoothing as outlined before (La Manno *et al.*, 2018; Fig 2E). The only difference is that we apply a scaling factor to φ in order to make the Gaussian kernel approximately symmetrical. The scaling factor we choose is the mean value of the radius of all data points in the DC1‐DC2 plot. The displacement value for grid points can then be scaled back by the same factor so that we can display the results in the φ‐DC3 plane. Here, we choose ϱ=2 (equation 38).

### State transition index of the attractor

If we compare the attractor (a stable manifold) to a water slide, then a person going down the water slide corresponds to a cell going through the cell cycle. The cell runs along the main path at the bottom of the channel, but it also veers toward the sides. The steepness of the walls, or the strength with which a cell is pushed back toward the middle, is called the attractor stability. The steeper the walls, the faster perturbations decay and the faster cells return to the attractor, hence the more stable it is.

The index for critical state transitions $I_{C}$ introduced in Mojtahedi *et al* (2016) is defined via

(40) $$I_{C}:=\frac{〈\left|R\left(g_{i},g_{j}\right)\right|〉}{〈R\left(S^{k},S^{l}\right)〉},$$

where $g_{i}$ are gene vectors, $S^{k}$ are cell states and $〈R(\ldots ,\ldots 〉〉$ is the average of all pairwise correlations (we utilize Spearman correlation in our analysis) (Mojtahedi *et al.*, 2016). We only include pairwise correlation values with a p‐value smaller than 0.05.

In polar coordinates, we divide the two‐dimensional cell cycle plot with respect to angle into 10 bins which contain the same number of cells each. For each of these bins, we calculate the state transition index $I_{C}$ as introduced by Mojtahedi *et al*. (2016).

In general, the state transition index increases before a critical state transition due to the fact that gene–gene correlation occurs coordinately and therefore increases on average, while the average cell–cell correlation decreases because cells are more variable during transitions than in steady state. Both of these effects would cause an increase in $I_{C}$ making the state transition index an appropriate measure for state transitions.

Our analysis of the behavior of $I_{C}$ throughout the cell cycle implies that there is no critical state transition detected as the progression observed in Appendix Fig S7A is homogeneous. Small changes are attributed to noise rather than orchestrated behavior.

### GO term analysis

Since the additional rotation R (equation 26), which we apply after PCA, is a linear algorithm, the weights $W^{T}$ (equation 15) generated by PCA can be transformed linearly as well by considering the rotated weights $U=R\cdot W^{T}$. We analyze the rotated weights from U and the difference to original weights from $W^{T}$ in order to gain an insight into the cyclic object we found in two dimensions.

A GO term analysis (Eden *et al.*, 2007; Eden *et al.*, 2009) of the first three columns of $W^{T}$, which are the weights generating the first three PCs, shows that all three PCs are highly dominated by cell cycle processes (see Appendix Table S2). We see highly significant p‐values suggesting that all three PCs are vital for the description of the cell cycle.

On the other hand, for the weights in U we observe that while DC1 and DC2 are still dominated by cell cycle effects with highly significant p‐values, DC3 is completely free of cell cycle GO terms (see Appendix Table S2; Eden *et al.*, 2007; Eden *et al.*, 2009).

Furthermore, we have investigated additional rotated PCs of lower order and have not found any GO terms (Eden *et al.*, 2007; Eden *et al.*, 2009) related to the cell cycle with a *P*‐value < 10^−5^. This is another strong indication that we have succeeded in isolating cell cycle effects into only two dimensions.

### Independent component analysis

Statistical independence between the components cannot be sufficiently concluded from our analysis of rotated principal components. Independent component analysis (ICA) represents the data by a linear combination of k factors where the k factors are chosen such that they are statistically independent (their joint distribution is equal to the product of their marginal distributions). The number of factors k has to be chosen by the user. A drawback of ICA is that there is no ordering of components with respect to "explained variance" as is the case with principal component analysis (PCA). Therefore, all resulting components have to be considered, adding importance to the choice of k. We have performed an ICA on the HeLa data that was mainly presented in the manuscript for multiple choices of $k\in \left\{3,\ldots ,10\right\}$.

We find that for any of these choices there are always two components such that their 2‐dimensional projection creates a circular data cloud. We illustrate this for the choice of k=6 in (Fig EV4A) as pairwise plots can still be visualized here and we find the first 6 principal components (PCs) to be of main importance (section “Explained variation and significance of principal components”). The ICA was done with the "fastICA" function from the "fastICA" package in R (Hyvarinen & Oja, 2000). This algorithm uses a stochastic optimizer for finding the k components, meaning each run of "fastICA" gives slightly different results. For example, the two components creating a circular data cloud as seen in Fig EV4A might not be IC2 and IC4 on the next run but another pair out of $\left\{1,\ldots ,6\right\}$. However, we always find exactly two ICs whose pairwise projection creates a circular data cloud.

Even though ICA does not produce an orthogonal transformation as PCA does, the algorithm still provides a weight matrix providing us information of how the original components in the data (individual species of mRNA) are weighted to create the projections. In Fig EV4B, we correlated the weight vectors corresponding to the 6 ICs to the weight vectors corresponding to the first 6 dynamical components (DCs) from our analysis (right‐hand plot). We observe that the two dynamical components DC1 and DC2 responsible for the cell cycle are highly correlated to exactly one independent component—IC4 and IC2, respectively. This suggests that there is a close correspondence between the two statistically independent components that create a cyclic data cloud which was identified by ICA and the two dynamical components we find to be responsible for cell cycle.

Analogously, the plot correlating the weights corresponding to the first principal components to the independent components can be viewed in Fig EV4B (left‐hand plot). We see that the correlation of PCs to IC2 and IC4 is much more broadly distributed and values > 0.9 are absent. This suggests that the additional step of rotation we introduced is in fact beneficial for identifying statistically independent sources of variation related to the cell cycle.

### Downsampling analysis

Since there are apparent differences in the quality of the cell cycle pattern in different data sets, we want to investigate the associated parameters and conditions for obtaining the pattern. We believe that there are two main parameters responsible for the pattern to reveal itself: the sequencing depth and the number of cells. The more deeply a cell population is sequenced, the fewer cells are required and vice versa. Due to the idiosyncrasies of single‐cell data sets, we believe there is no gold‐standard threshold for either of these parameters, but we attempt to estimate a range for them.

Firstly, we have performed a downsampling analysis on the HeLa data to investigate the emergence of a two‐dimensional cyclic structure with dependence on the sequencing depth. We downsample with the function "downsampleMatrix" from the R package "DropletUtils" (Griffiths *et al*, 2018; Lun *et al*, 2019). We set the parameter bycol = TRUE and provide different coefficients k for downsampling. The coefficient k determines what fraction of reads of an original cell will be preserved on average. We choose $k\in \left\{0.05,0.1,0.15,\ldots ,0.95\right\}$ and rerun our algorithm for each new data set. The resulting cell cycle for $k\in \left\{0.1,0.2,\ldots ,0.9\right\}$ illustrates the slow collapse of the cell cycle to the center with decreasing sequencing depth (decreasing k) (Fig EV2A). We kept the plot boundaries constant to emphasize this collapse.

In order to characterize the cyclic data cloud, we consider the mean, standard deviation, and confidence interval of the radii for each cell cycle corresponding to its downsampling coefficient k (Fig EV2B). The black lines represent linear regressions and their slope, p‐value and $R^{2}$ are displayed in the lower right‐hand corner. We observe surprisingly clear linear relationships between the downsampling coefficent k and the mean of the radius, the standard deviation, and the lower bound of the confidence interval of the radius, respectively.

The HeLa data presented have been sequenced exceptionally deep. At 20% sequencing depth and after usual filtering, we maintain on average 3,000 UMIs per cell. While we did not perform a data mining analysis to determine the average sequencing depth of published single‐cell sequencing data, we feel that 3.000 UMIs per cell is still on the upper end of the distribution. We observe though that at 20% sequencing depth, the lower confidence interval bound has a radius of 0.32 which visually makes it appear rather as a disk than an annulus. For 10% sequencing depth, the cell cycle signal appears to mostly break down.

It is unfortunately difficult to determine a single measure by which one can judge whether the cyclic transcriptome pattern appears or not. It is rather a combination of characteristics which is why we emphasized the verification of the two‐dimensional cell cycle in the manuscript. The most important characteristics are the DC1‐DC2‐plot, the time course of total UMI counts, the cell cycle cluster and marker score and the variation decomposition. If all five of these characteristics imply that the cell cycle signal has been isolated into two dimensions, we conclude that the data set is suitable for our approach.

The second aspect influencing the emergence of the cyclic transcriptome pattern is the number of cells. We randomly sampled different amounts of cells from our HeLa data and reran the algorithm similar to the previous analysis. In Fig EV2C, we, interestingly, do not observe a collapsing cell cycle in the origin, but rather a thinning of the cell cycle due to fewer cells present. Again, investigating the characteristics of the radii, we do not observe a similar linear relationship as before but rather a breaking point (Fig EV2D).

This suggests that the characteristics of the data cloud shift noticeably after fewer than 25% of the cells are used for the analysis. Considering our additional measures (time course of total UMI counts, marker and cluster score and variation decomposition) suggests that at around 40% of the sampled cells we get a clear separation of the cell cycle into two dimensions which corresponds to approximately 600 cells.

We repeated the downsampling analysis for the data set containing only 40% of the cells and observed that the required sequencing depth is slightly higher at around 4,500 UMIs per cell (roughly 30% of the original sequencing depth).

We conclude that for the two‐dimensional transcriptome pattern to emerge, a certain number of cells are required. This threshold itself most likely depends on the sequencing depth. On the other hand, the sequencing depth and characteristics (mean of the radius, standard deviation of the radius, and the resulting confidence interval) of the two‐dimensional data cloud appear to have a linear dependency. We suggest a data set to contain around 600 cells to obtain reasonable coverage of the entire cell cycle in transcriptome space. Such a data set should then contain a mean UMI count of roughly 4,500 UMIs. If a data set contains significantly more cells, the mean UMI count can be lower but should for most cases still exceed around 3,000 UMIs per cell.

### Single‐cell sequencing for data set 1: Drop‐seq procedure, single‐cell library generation, and sequencing

The Drop‐seq runs and library preparation were performed as described in Alles *et al* (2017) on a self‐built Drop‐seq setup (Macosko *et al.*, 2015).

HeLaS3 cells and HeLaS3 AGO2KO cells were grown to the logarithmic phase, pelleted by centrifugation (300 *g*, 5 min), fixed with 80% cold methanol while mildly vortexing and kept on ice until the run. Fixed cells were prepared for the Drop‐seq run by centrifugation (1,000 *g*, 5 min) and resuspension in 1 ml of PBS‐BSA 0.01% + RiboLock (Thermo Fisher) (0.8 U/µl), followed by another centrifugation (1,000 *g*, 5 min) and resuspension in 0.5 ml of PBS‐BSA 0.01% + RiboLock (Thermo Fisher) (0.8 U/µl). Then, cells were passed through a cell strainer (35 µm), counted, diluted with PBS‐BSA 0.01% to a concentration of 100 cells/µl, and transferred into a syringe to be loaded on the Drop‐seq apparatus. After mixing with lysis buffer, this corresponds to a final concentration of 50 cells/µl in the droplets.

The two single‐cell libraries from the HeLaS3 and HeLaS3 AGO2KO cells (1.8 pM, final insert sizes 700 bp) were sequenced in paired end mode on Illumina Nextseq 500, together with two other libraries, yielding ~ 49 × 10^6^ read pairs for the HeLaS3 library and ~ 45 × 10^6^ read pairs for the HeLa AGO2KO library.

A second sequencing run was performed with two multiplexed libraries prepared from a subpool of cells (50%) in order to obtain deeper sequencing data from less cells (1.8 pM, final insert sizes 645 and 628 bp). This yielded ~ 168 × 10^6^ read pairs for the HeLaS3 library and ~ 186 × 10^6^ read pairs for the HeLa AGO2KO library.

Read 1: 20 bp (bases 1–12 cell barcode, bases 13–20 UMI; Drop‐seq custom primer 1 “Read1CustSeqB”), index read: 8 bp, read 2 (paired end): 64 bp).

### Single‐cell sequencing for data set 2: Drop‐seq procedure, single‐cell library generation, and sequencing

The Drop‐seq runs and library preparation were performed as described in Alles *et al.* (2017) on a self‐built Drop‐seq set‐up (Macosko *et al.*, 2015).

HeLaS3 cells were grown to the logarithmic phase, pelleted by centrifugation (300 *g*, 5 min), fixed with 80% cold methanol while mildly vortexing and kept on ice until the run. Fixed cells were prepared for the Drop‐seq run by centrifugation (1,000 *g*, 5 min) and resuspension in 1 ml of PBS‐BSA 0.01% + RiboLock (Thermo Fisher) (0.8 U/µl), followed by another centrifugation (1,000 g, 5 min) and resuspension in 0.5 ml of PBS‐BSA 0.01% + RiboLock (Thermo Fisher) (0.8 U/µl). Then, cells were passed through a cell strainer (35 µm), counted, diluted with PBS‐BSA 0.01% to a concentration of 100 cells/µl, and transferred into a syringe to be loaded on the Drop‐seq apparatus. After mixing with lysis buffer, this corresponds to a final concentration of 50 cells/µl in the droplets.

The single‐cell final library (1.8 pM, final insert sizes 532 bp) was sequenced in paired end mode on Illumina Nextseq 500 75 cycles high output, together with three other libraries, yielding ~ 100 × 10^6^ read pairs for the HeLaS3 library.

Read 1: 20 bp (bases 1–12 cell barcode, bases 13–20 UMI; Drop‐seq custom primer 1 “Read1CustSeqB”), index read: 8 bp, read 2 (paired end): 64 bp).

### Processing of raw sequencing data sets

The sequencing quality was assessed by FastQC v.0.11.2 (Andrews, 2010). We used the Drop‐seq tools v.2.0.0 (Macosko *et al.*, 2015) to tag the sequences with their corresponding cell and molecular barcodes, to trim poly(A) stretches and potential SMART adapter contaminants and to filter out barcodes with low‐quality bases. The reads were then aligned to a GRCh38 reference genome (Schneider *et al*, 2017), using STAR v.2.6.0c (Dobin *et al*, 2013) with default parameters and sorted using samtools v.1.9 (Li *et al*, 2009).

The Drop‐seq tool was further exploited to add gene annotation tags to the aligned reads and to identify and correct some of the bead synthesis errors. The number of cells was determined by extracting the number of reads per cell, then plotting the cumulative distribution of reads against the cell barcodes ordered by descending number of reads and selecting the inflection knee point of the distribution using dropbead v.0.25 (Alles *et al.*, 2017). Finally, the DigitalExpression tool (Macosko *et al.*, 2015) was used to obtain the digital gene expression (DGE) matrix for each sample. DGE matrix with only intronic reads was created by specifying the list of functional annotations.

## Author contributions

Research conception, experiment design, supervision: NR; Experiments, data analysis: SF; Theoretical method design, data analysis: DS; Theoretical method design, supervision: MF; Manuscript writing: DS, SF, JPJ, MF, NR.

## Conflict of interest

The authors declare that they have no conflict of interest.

## Supporting information



Appendix

Expanded View Figures PDF

Dataset EV1

Review Process File

## Data Availability

The datasets and computer code produced in this study are available in the following databases:
RNA‐Seq data: Gene Expression Omnibus GSE142277 (https://www.ncbi.nlm.nih.gov/geo/query/acc.cgi?acc=GSE142277). RNA‐Seq data: Gene Expression Omnibus GSE142356 (https://www.ncbi.nlm.nih.gov/geo/query/acc.cgi?acc=GSE142356). Computational analysis scripts: GitHub (https://github.com/danielschw188/Revelio).

## References

[msb20209946-bib-0001] Alberts B , Johnson A , Lewis J , Raff M , Roberts K , Walter P (2015) Molecular biology of the cell, New York, NY: Garland Science

[msb20209946-bib-0002] Alles J , Karaiskos N , Praktiknjo SD , Grosswendt S , Wahle P , Ruffault PL , Ayoub S , Schreyer L , Boltengagen A , Birchmeier C *et al* (2017) Cell fixation and preservation for droplet‐based single‐cell transcriptomics. BMC Biol 15: 44 28526029 10.1186/s12915-017-0383-5 PMC5438562

[msb20209946-bib-0003] Alter O , Brown PO , Botstein D (2000) Singular value decomposition for genome‐wide expression data processing and modeling. Proc Natl Acad Sci USA 97: 10101–10106 10963673 10.1073/pnas.97.18.10101 PMC27718

[msb20209946-bib-0004] Andrews S (2010) FastQC: a quality control tool for high throughput sequence data. Available at: http://www.bioinformatics.babraham.ac.uk/projects/fastqc

[msb20209946-bib-0005] Arnold VI (1992) Ordinary Differential Equations, Berlin: Springer

[msb20209946-bib-0006] Barron M , Li J (2016) Identifying and removing the cell‐cycle effect from single‐cell RNA‐Sequencing data. Sci Rep 6: 33892 27670849 10.1038/srep33892 PMC5037372

[msb20209946-bib-0007] Bendall SC , Davis KL , Amir ED , Tadmor MD , Simonds EF , Chen TJ , Shenfeld DK , Nolan GP , Pe'er D (2014) Single‐cell trajectory detection uncovers progression and regulatory coordination in human B cell development. Cell 157: 714–725 24766814 10.1016/j.cell.2014.04.005 PMC4045247

[msb20209946-bib-0008] Brandeis M , Rosewell I , Carrington M , Crompton T , Jacobs MA , Kirk J , Gannon J , Hunt T (1998) Cyclin B2‐null mice develop normally and are fertile whereas cyclin B1‐null mice die in utero. Proc Natl Acad Sci USA 95: 4344–4349 9539739 10.1073/pnas.95.8.4344 PMC22491

[msb20209946-bib-0009] Buettner F , Natarajan KN , Casale FP , Proserpio V , Scialdone A , Theis FJ , Teichmann SA , Marioni JC , Stegle O (2015) Computational analysis of cell‐to‐cell heterogeneity in single‐cell RNA‐sequencing data reveals hidden subpopulations of cells. Nat Biotechnol 33: 155–160 25599176 10.1038/nbt.3102

[msb20209946-bib-0010] Butler A , Hoffman P , Smibert P , Papalexi E , Satija R (2018) Integrating single‐cell transcriptomic data across different conditions, technologies, and species. Nat Biotechnol 36: 411–420 29608179 10.1038/nbt.4096 PMC6700744

[msb20209946-bib-0011] Cheng A , Solomon MJ (2008) Speedy/Ringo C regulates S and G2 phase progression in human cells. Cell Cycle 7: 3037–3047 18802405 10.4161/cc.7.19.6736 PMC2592538

[msb20209946-bib-0012] Csikász‐Nagy A , Battogtokh D , Chen KC , Novák B , Tyson JJ (2006) Analysis of a generic model of eukaryotic cell‐cycle regulation. Biophys J 90: 4361–4379 16581849 10.1529/biophysj.106.081240 PMC1471857

[msb20209946-bib-0013] Dobin A , Davis CA , Schlesinger F , Drenkow J , Zaleski C , Jha S , Batut P , Chaisson M , Gingeras TR (2013) STAR: ultrafast universal RNA‐seq aligner. Bioinformatics 29: 15–21 23104886 10.1093/bioinformatics/bts635 PMC3530905

[msb20209946-bib-0014] Dominguez D , Tsai YH , Gomez N , Jha DK , Davis I , Wang Z (2016) A high‐resolution transcriptome map of cell cycle reveals novel connections between periodic genes and cancer. Cell Res 26: 946–962 27364684 10.1038/cr.2016.84 PMC4973334

[msb20209946-bib-0015] Eden E , Lipson D , Yogev S , Yakhini Z (2007) Discovering motifs in ranked lists of DNA sequences. PLoS Comput Biol 3: e39 17381235 10.1371/journal.pcbi.0030039 PMC1829477

[msb20209946-bib-0016] Eden E , Navon R , Steinfeld I , Lipson D , Yakhini Z (2009) GOrilla: a tool for discovery and visualization of enriched GO terms in ranked gene lists. BMC Bioinformatics 10: 48 19192299 10.1186/1471-2105-10-48 PMC2644678

[msb20209946-bib-0017] Gérard C , Goldbeter A (2009) Temporal self‐organization of the cyclin/Cdk network driving the mammalian cell cycle. Proc Natl Acad Sci USA 106: 21643–21648 20007375 10.1073/pnas.0903827106 PMC2799800

[msb20209946-bib-0018] Gérard C , Goldbeter A (2011) A skeleton model for the network of cyclin‐dependent kinases driving the mammalian cell cycle. Interface Focus 1: 24–35 22419972 10.1098/rsfs.2010.0008 PMC3262247

[msb20209946-bib-0019] Giotti B , Joshi A , Freeman TC (2017) Meta‐analysis reveals conserved cell cycle transcriptional network across multiple human cell types. BMC Genom 18: 30 10.1186/s12864-016-3435-2 PMC5217208 28056781

[msb20209946-bib-0020] Griffiths JA , Richard AC , Bach K , Lun ATL , Marioni JC (2018) Detection and removal of barcode swapping in single‐cell RNA‐seq data. Nat Commun 9: 2667 29991676 10.1038/s41467-018-05083-x PMC6039488

[msb20209946-bib-0021] Hahn AT , Jones JT , Meyer T (2009) Quantitative analysis of cell cycle phase durations and PC12 differentiation using fluorescent biosensors. Cell Cycle 8: 1044–1052 19270522 10.4161/cc.8.7.8042 PMC2668240

[msb20209946-bib-0022] Haken H (1983) Synergetics: An Introduction, Berlin: Springer

[msb20209946-bib-0023] Hyvärinen A , Oja E (2000) Independent component analysis: algorithms and applications. Neural Netw 13: 411–430 10946390 10.1016/s0893-6080(00)00026-5

[msb20209946-bib-0024] Kester L , van Oudenaarden A (2018) Single‐cell transcriptomics meets lineage tracing. Cell Stem Cell 23: 166–179 29754780 10.1016/j.stem.2018.04.014

[msb20209946-bib-0025] Kuznetsov Y (1998) Elements of applied bifurcation theory, New York, NY: Springer

[msb20209946-bib-0026] La Manno G , Soldatov R , Zeisel A , Braun E , Hochgerner H , Petukhov V , Lidschreiber K , Kastriti ME , Lönnerberg P , Furlan A *et al* (2018) RNA velocity of single cells. Nature 560: 494–498 30089906 10.1038/s41586-018-0414-6 PMC6130801

[msb20209946-bib-0027] Laudadio I , Orso F , Azzalin G , Calabrò C , Berardinelli F , Coluzzi E , Gioiosa S , Taverna D , Sgura A , Carissimi C *et al* (2019) AGO2 promotes telomerase activity and interaction between the telomerase components TERT and TERC. EMBO Rep 20: e45969 30591524 10.15252/embr.201845969 PMC6362350

[msb20209946-bib-0028] Leng N , Chu LF , Barry C , Li Y , Choi J , Li X , Jiang P , Stewart RM , Thomson JA , Kendziorski C (2015) Oscope identifies oscillatory genes in unsynchronized single‐cell RNA‐seq experiments. Nat Methods 12: 947–950 26301841 10.1038/nmeth.3549 PMC4589503

[msb20209946-bib-0029] Li H , Handsaker B , Wysoker A , Fennell T , Ruan J , Homer N , Marth G , Abecasis G , Durbin R , 1000 Genome Project Data Processing Sub Group (2009) The Sequence Alignment/Map format and SAMtools. Bioinformatics 25: 2078–2079 19505943 10.1093/bioinformatics/btp352 PMC2723002

[msb20209946-bib-0030] Liu Y , Chen S , Wang S , Soares F , Fischer M , Meng F , Du Z , Lin C , Meyer C , DeCaprio JA *et al* (2017a) Transcriptional landscape of the human cell cycle. Proc Natl Acad Sci USA 114: 3473–3478 28289232 10.1073/pnas.1617636114 PMC5380023

[msb20209946-bib-0031] Liu Z , Lou H , Xie K , Wang H , Chen N , Aparicio OM , Zhang MQ , Jiang R , Chen T (2017b) Reconstructing cell cycle pseudo time‐series via single‐cell transcriptome data. Nat Commun 8: 22 28630425 10.1038/s41467-017-00039-z PMC5476636

[msb20209946-bib-0032] Lun ATL , Riesenfeld S , Andrews T , Dao TP , Gomes T , Participants in the 1st Human Cell Atlas Jamboree , Marioni JC (2019) EmptyDrops: distinguishing cells from empty droplets in droplet‐based single‐cell RNA sequencing data. Genome Biol 20: 63 30902100 10.1186/s13059-019-1662-y PMC6431044

[msb20209946-bib-0033] Macosko EZ , Basu A , Satija R , Nemesh J , Shekhar K , Goldman M , Tirosh I , Bialas AR , Kamitaki N , Martersteck EM *et al* (2015) Highly Parallel genome‐wide expression profiling of individual cells using nanoliter droplets. Cell 161: 1202–1214 26000488 10.1016/j.cell.2015.05.002 PMC4481139

[msb20209946-bib-0034] Mojtahedi M , Skupin A , Zhou J , Castaño IG , Leong‐Quong RYY , Chang H , Trachana K , Giuliani A , Huang S (2016) Cell Fate Decision as High‐Dimensional Critical State Transition. PLoS Biol 14: e2000640 28027308 10.1371/journal.pbio.2000640 PMC5189937

[msb20209946-bib-0035] Morgan DO (2006) The cell cycle: principles of control, London: Oxford Univ. Press

[msb20209946-bib-0036] Santos A , Wernersson R , Jensen LJ (2015) Cyclebase 3.0: a multi‐organism database on cell‐cycle regulation and phenotypes. Nucleic Acids Res 43: D1140–D1144 25378319 10.1093/nar/gku1092 PMC4383920

[msb20209946-bib-0037] Schneider VA , Graves‐Lindsay T , Howe K , Bouk N , Chen HC , Kitts PA , Murphy TD , Pruitt KD , Thibaud‐Nissen F , Albracht D *et al* (2017) Evaluation of GRCh38 and de novo haploid genome assemblies demonstrates the enduring quality of the reference assembly. Genome Res 27: 849–864 28396521 10.1101/gr.213611.116 PMC5411779

[msb20209946-bib-0038] Setty M , Tadmor MD , Reich‐Zeliger S , Angel O , Salame TM , Kathail P , Choi K , Bendall S , Friedman N , Pe'er D (2016) Wishbone identifies bifurcating developmental trajectories from single‐cell data. Nat Biotechnol 34: 637–645 27136076 10.1038/nbt.3569 PMC4900897

[msb20209946-bib-0039] Soni DV , Sramkoski RM , Lam M , Stefan T , Jacobberger JW (2008) Cyclin B1 is rate limiting but not essential for mitotic entry and progression in mammalian somatic cells. Cell Cycle 7: 1285–1300 18414058 10.4161/cc.7.9.5711

[msb20209946-bib-0040] Strauss B , Harrison A , Coelho PA , Yata K , Zernicka‐Goetz M , Pines J (2018) Cyclin B1 is essential for mitosis in mouse embryos, and its nuclear export sets the time for mitosis. J Cell Biol 217: 179–193 29074707 10.1083/jcb.201612147 PMC5748970

[msb20209946-bib-0041] Swinbank RJ , Purser R (2006) Fibonacci grids: A novel approach to global modelling. Quarterly Journal of the Royal Meteorological Society 132: 1769–1793

[msb20209946-bib-0042] Tanay A , Regev A (2017) Scaling single‐cell genomics from phenomenology to mechanism. Nature 541: 331–338 28102262 10.1038/nature21350 PMC5438464

[msb20209946-bib-0043] Trapnell C , Cacchiarelli D , Grimsby J , Pokharel P , Li S , Morse M , Lennon NJ , Livak KJ , Mikkelsen TS , Rinn JL (2014) The dynamics and regulators of cell fate decisions are revealed by pseudotemporal ordering of single cells. Nat Biotechnol 32: 381–386 24658644 10.1038/nbt.2859 PMC4122333

[msb20209946-bib-0044] Vogel H (1979) A better way to construct the sunflower head. Math Biosci 44: 179–189

[msb20209946-bib-0045] Voss TC , Hager GL (2013) Dynamic regulation of transcriptional states by chromatin and transcription factors. Nat Rev Genet 15: 69–81 24342920 10.1038/nrg3623 PMC6322398

[msb20209946-bib-0046] Whitfield ML , Sherlock G , Saldanha AJ , Murray JI , Ball CA , Alexander KE , Matese JC , Perou CM , Hurt MM , Brown PO *et al* (2002) Identification of genes periodically expressed in the human cell cycle and their expression in tumors. Mol Biol Cell 13: 1977–2000 12058064 10.1091/mbc.02-02-0030. PMC117619

[msb20209946-bib-0047] Wolf FA , Hamey FK , Plass M , Solana J , Dahlin JS , Göttgens B , Rajewsky N , Simon L , Theis FJ (2019) PAGA: graph abstraction reconciles clustering with trajectory inference through a topology preserving map of single cells. Genome Biol 20: 59 30890159 10.1186/s13059-019-1663-x PMC6425583

[msb20209946-bib-0048] Xia C , Fan J , Emanuel G , Hao J , Zhuang X (2019) Spatial transcriptome profiling by MERFISH reveals subcellular RNA compartmentalization and cell cycle‐dependent gene expression. Proc Natl Acad Sci USA 116: 19490–19499 31501331 10.1073/pnas.1912459116 PMC6765259

