# Supplementary material for: The transcriptome dynamics of single cells during the cell cycle
Source: Mol Syst Biol. 2020 Nov 18;16(11):e9946. doi: 10.15252/msb.20209946 (PMC7672610; doi:10.15252/msb.20209946)
Supplement: Supplementary file 1 — Appendix [file MSB-16-e9946-s001.pdf]

Appendix for "The transcriptome dynamics of single cells during the cell cycle"

Authors: Daniel Schwabe[1], Sara Formichetti[2,3], Jan Philipp Junker[4], Martin Falcke[1,5,*] and Nikolaus Rajewsky[2,*]

Affiliations:
[1]Mathematical Cell Physiology, Max Delbrück Center for Molecular Medicine in the Helmholtz Association, Robert-Rössle-Str. 10, 13125 Berlin, Germany.
[2]Systems Biology of Gene Regulatory Elements, Berlin Institute for Medical Systems Biology, Max Delbrück Center for Molecular Medicine in the Helmholtz Association, Hannoversche Str. 28, 10115 Berlin, Germany.
[3]Epigenetics and Neurobiology Unit, European Molecular Biology Laboratory, Via Ramarini 32, 00015, Monterotondo, Italy.
[4]Quantitative Developmental Biology, Berlin Institute for Medical Systems Biology, Max Delbrück Center for Molecular Medicine in the Helmholtz Association, Hannoversche Str. 28, 10115 Berlin, Germany.
[5]Department of Physics, Humboldt University Berlin, Newtonstr. 15, 12489 Berlin, Germany.

*Correspondence to: martin.falcke@mdc-berlin.de and rajewsky@mdc-berlin.de

Table of Contents

Appendix Table S1.Summary of sequencing data analyzed.

Appendix Table S2.Excel file containing GO term analysis (Eden *et al*, 2007; Eden *et al*, 2009).

Appendix Figure S1.Data set 2 (HeLa cells) confirms that a two-dimensional annulus represents the cell cycle in HeLa cell populations.

Appendix Figure S2.Data set 3 (HEK cells) confirms that our observations and the algorithm are transferable to a different cell type.

Appendix Figure S3.The cell cycle properties appear to be preserved across species as the cell cycle in mouse 3T3 cells is also shown to form a cyclic object in two dimensions.

Appendix Figure S4.HeLa data set 1.2 utilizing more than 10.000 genes yields two-dimensional cycle after rotation of PCA.

Appendix Figure S5.Placement of cells near the origin is not caused by apoptosis.

Appendix Figure S6.Statistically, the radius of the points of the data cloud fit a normal distribution, implying an annular shape.

Appendix Figure S7.State transition index (Mojtahedi *et al*, 2016) along the cell cycle does not indicate a critical state transition.

Appendix Figure S8.Average trajectories for different height ranges of the cylinder in four data sets.

Appendix Figure S9.Other methods are less successful than Revelio at removing only cell cycle effects from data.

Appendix Figure S10.Variation decomposition with respect to a random gene set exhibits similar distributions across all components.

Appendix Figure S11.The cumulative phase space density of cells (blue) is reasonably well approximated by a uniform distribution (black).

Appendix Figure S12.Variance explained per component (see section "Explained Variation and Significance of Principal Components" in Materials and Methods).

Appendix Figure S13.When processed with the Revelio algorithm, a negative control of non-cycling peripheral blood mononuclear cells (PBMC) exhibits no periodic data structure.

ID	cell type	availability	#cells	variable genes utilized?	#genes used during PCA	mean UMI count	displayed in figure or table
data set 1.1	HeLaS3	https://www.ncbi.nlm.nih.gov/geo/query/acc.cgi?acc=GSE142277	1.477	Yes	1.031	11.206	Fig 1,2,4<br>Fig EV2,EV3,EV4,EV5<br>Appendix Table S2<br>Appendix Figure S5-S12
data set 2	HeLaS3	https://www.ncbi.nlm.nih.gov/geo/query/acc.cgi?acc=GSE142356	2.537	Yes	543	4.547	Appendix Figure S1,S7,S8
data set 3	HEK293T	Alles et al., BMC Biology (Alles *et al*, 2017)	848	Yes	1.299	6.748	Appendix Figure S2,S7,S8
data set 4	3T3	Alles et al., BMC Biology (Alles *et al.*, 2017)	837	Yes	1.106	7.742	Appendix Figure S3,S7,S8
data set 1.2	HeLaS3	https://www.ncbi.nlm.nih.gov/geo/query/acc.cgi?acc=GSE142277	1.477	No	12.773	11.206	Appendix Figure S4,S7

Appendix Table S1.Summary of sequencing data analyzed.

Appendix Table S2.Excel file containing GO term analysis (Eden *et al.*, 2007; Eden *et al.*, 2009).

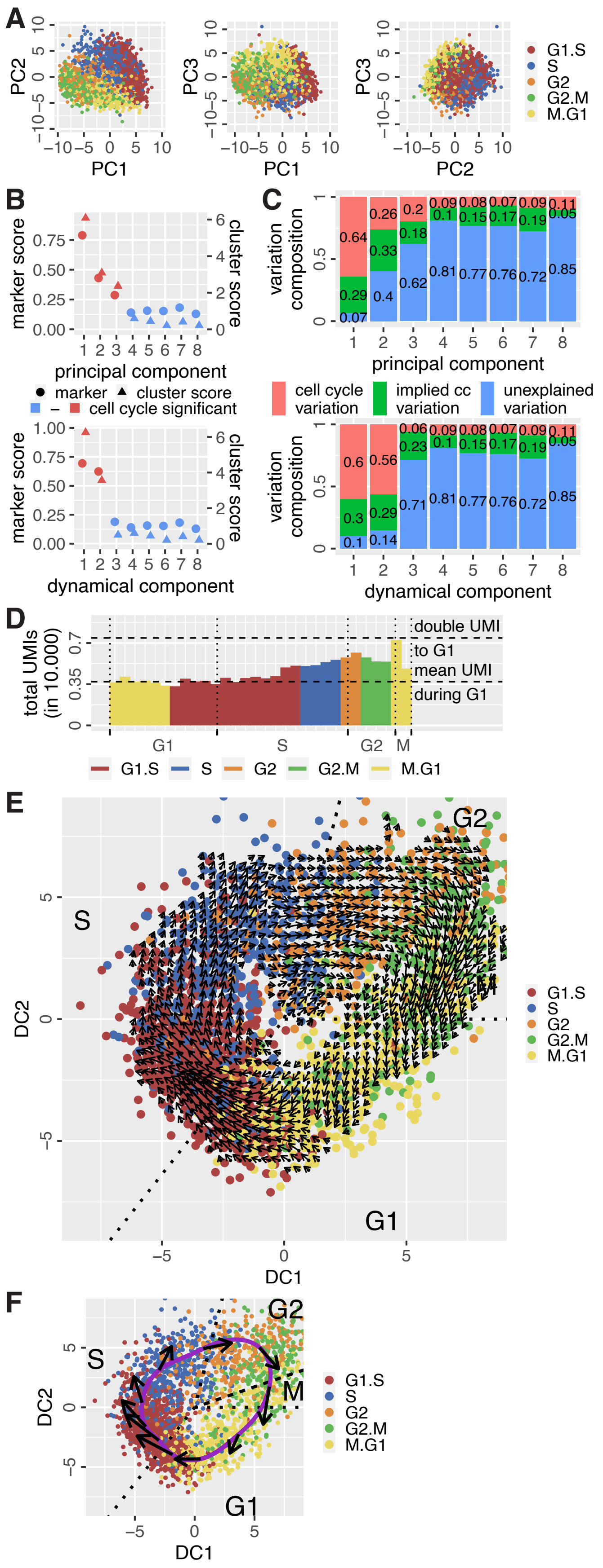

Appendix Figure S1.Data set 2 (HeLa cells) confirms that a two-dimensional annulus represents the cell cycle in HeLa cell populations.
This data set has almost twice as many cells as the previous data set 1 at less than half the sequencing depth.
A Pairwise PC plots again indicate the dynamical components to be slanted with respect to the original PCs. A clear periodic structure is completely absent.
B (top) Again the cell cycle cluster and marker scores concur with our observations from the PC plots. (bottom) The algorithm manages to place all cell cycle effects w.r.t. clustering and marker scores within the first two components.
C (top) The first three PCs contain noticeable cell cycle effects when variation in each component is divided into different sources (see Materials and Methods). (bottom) The variation decomposition again demonstrates that data variation in DC1 and DC2 is mainly caused by the cell cycle while other dimensions are clear of such effects.
D The increase of average total UMI count per interval and the drop between last and first bins are consistent with our previous observations.
E After rotation and the overlaying of RNA velocity, a clear cell cycle in the form of an annulus is observed. Motion of the cells appears strongest during G1-S and M phase similar to the data set 1.
F The average RNA velocity per interval is mostly tangential to the trajectory of an averaged cell.

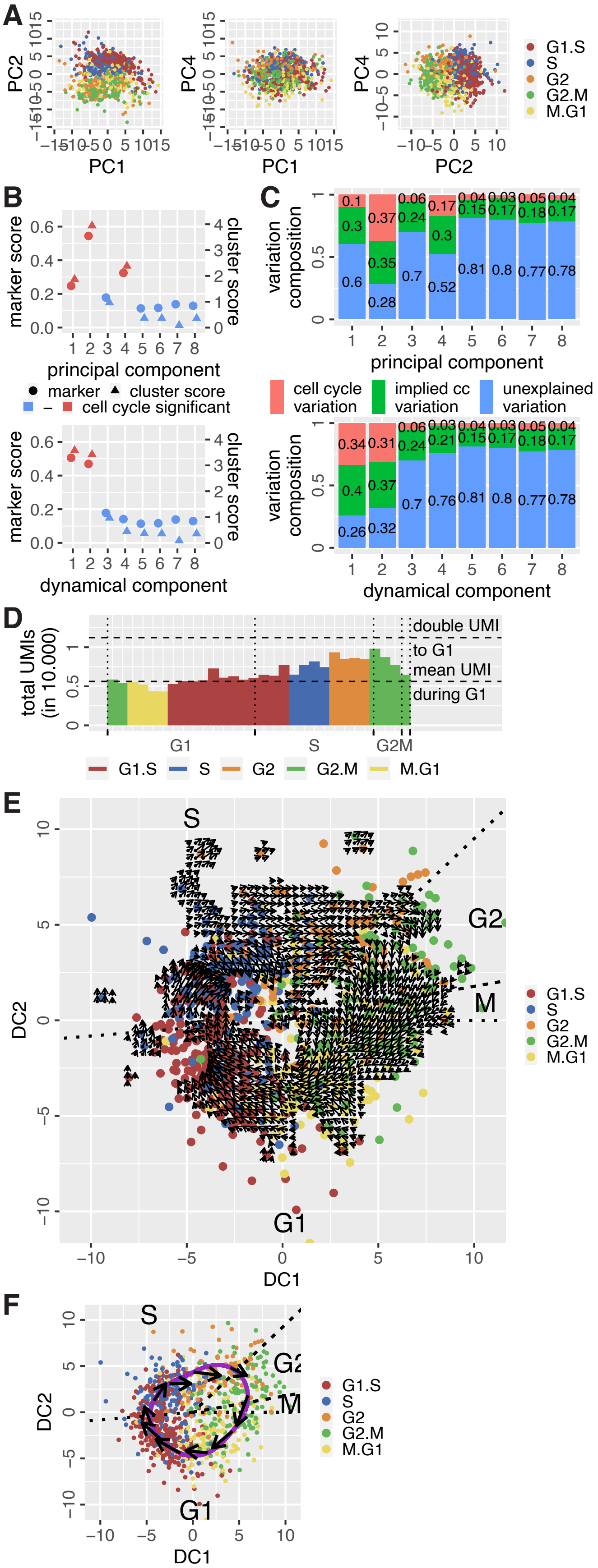

Appendix Figure S2.Data set 3 (HEK cells) confirms that our observations and the algorithm are transferable to a different cell type.

A Pairwise PC plots show clustering within the first three components but are very noisy and lack periodic structures.

B+C The cell cycle marker and cluster scores and the variation decomposition confirm our observations and is in line with previous data sets. We notice that the cell cycle is contained in PC1, PC2 and PC4 in this data set.

D The drop by factor 1/2 is less clear than what we saw in HeLa data but an increase in UMI counts towards M phase is nevertheless present. We suspect the data to be noisier since we operate with half as many cells as data set 1 and with 2/3 of the sequencing depth.

E Rotation of the space once again yields a clear periodic object with clustering according to inferred cell cycle phases. RNA velocity confirms the motion of the cells.

F The average velocity on the average cell is mostly tangential.

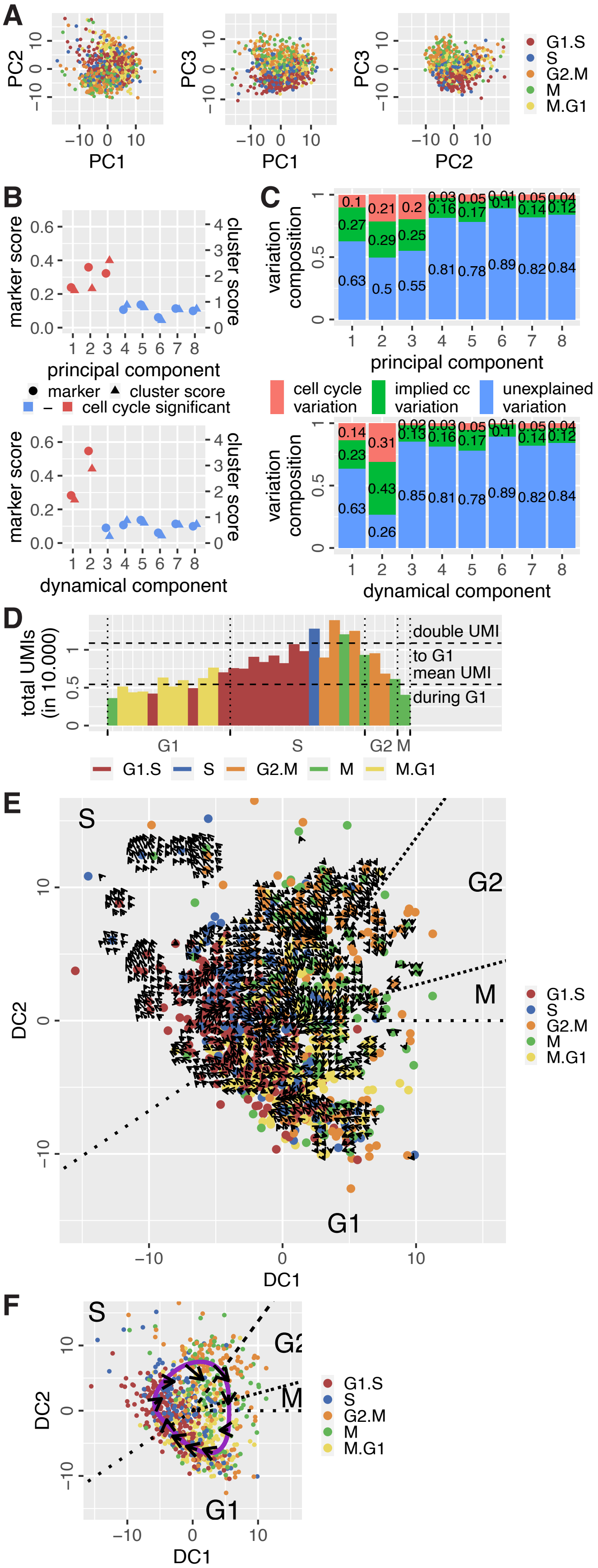

Appendix Figure S3.The cell cycle properties appear to be preserved across species as the cell cycle in mouse 3T3 cells is also shown to form a cyclic object in two dimensions.

A Similar to the human cell lines, pairwise PC plots show clustering w.r.t. cell cycle phases but are not periodic.

B The cell cycle marker and cluster score of PCs and DCs show similar patterns as before. We notice that the separation of cell cycle significant components (PC1, PC2, PC3) is less clear than for previous data sets. We similarly observe that the cell cycle signal in DC1 is noticeably smaller than in DC2. However, all cell cycle significant signals are still placed into the first two dimensions by the algorithm

C The variation decomposition for DC1 confirms that while there are significant cell cycle influences, the portion of unexplained variation is higher than previously seen.

D Similar to the HEK data set, the drop in average total UMI counts along the cell cycle is less clear and appears to happen slightly earlier than previous data sets. As before, we suspect additional noise incorporated into the data set due to the shallower sequencing depth to be the cause for this.

E While the rotation of PC space does improve the variation decomposition, the resulting two-dimensional periodic object is much noisier and clusters are less well separated than previously. The distinctions about variability in velocity magnitude are less clear than before due to high amounts of suspected noise.

F The average RNA velocity on an average individual cell is tangent in multiple along the majority of the cycle. However, there is a tendency of inward motion.

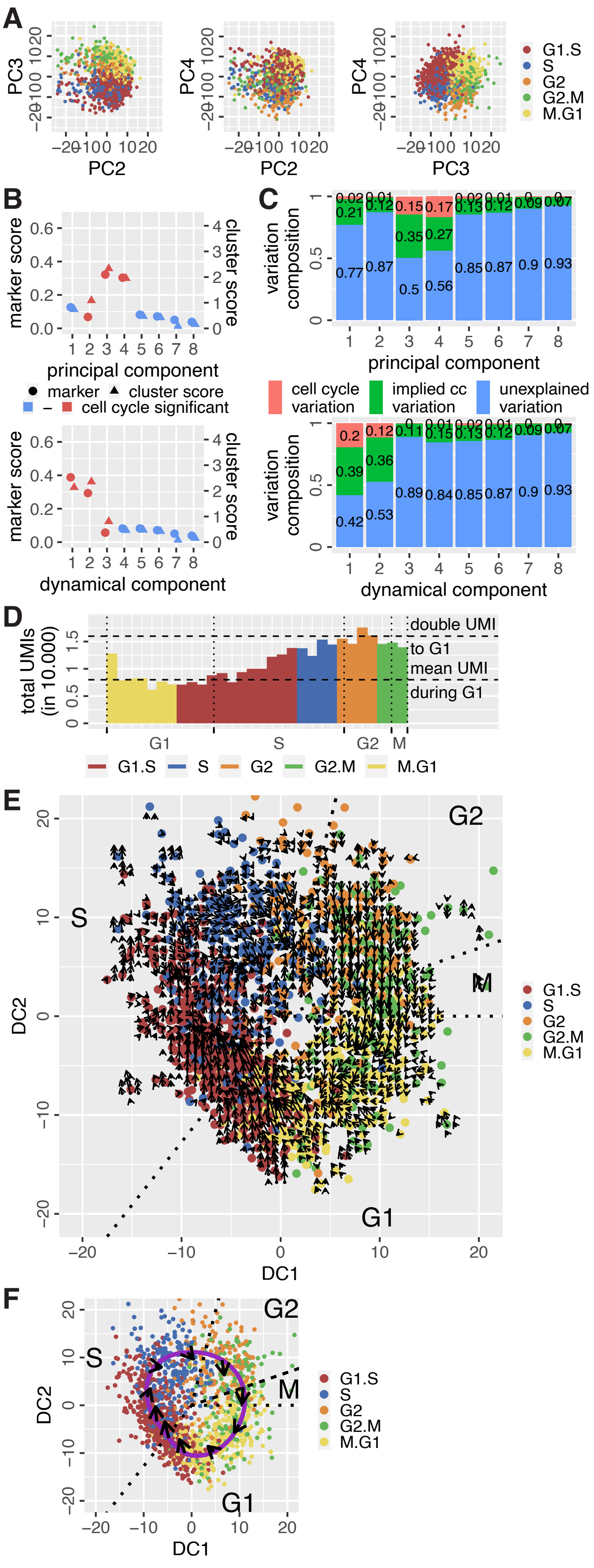

Appendix Figure S4.HeLa data set 1.2 utilizing more than 10.000 genes yields two-dimensional cycle after rotation of PCA.

The same data as in Fig 1,2 is analyzed again utilizing all genes for which exonic and intronic reads were measured.

A Pairwise PC plots of PC2, PC3, PC4. PC3 and PC4 on their own already form an acceptable cell cycle.

B (top) The cell cycle cluster and marker scores already point to PC3 and PC4 containing most cell cycle effects. (bottom) After rotation, all cell cycle effects are contained in DC1 and DC2. Additional dimensions exhibit only very small cluster scores.

C Due to the fact that the ratio of amount of cell cycle genes to total genes is much smaller in this data set, the biggest cell cycle contributors still have noticeable portions of unexplained variation even in DC1 and DC2.

D Progression of average total UMI count per interval along the cell cycle shows a sharp drop between the first and second bin, where cell division is suspected to take place. This points to the fact that synchronization of cell division to the positive part of the x-axis was not completely accurate (see Materials and Methods).This suggests that time courses derived from this data should be shifted slightly when correlating to pseudotime.

E By rotation and overlaying RNA velocity, the cell cycle becomes much clearer and forms an annulus in two dimensions. Our analysis from Fig 2 holds as the strongest average motion of cells takes place during G1-S and M phase.

F An average spline is added (magenta) which we interpret as an average cell trajectory. The average RNA velocity is approximately tangent to the cyclic trajectory, though there is an inward tendency. We suspect this is due to the fact that >10.000 genes are used for the velocity analysis. Since intronic read information is much sparser than exonic read information, a bias towards intronic dropouts could cause this effect.

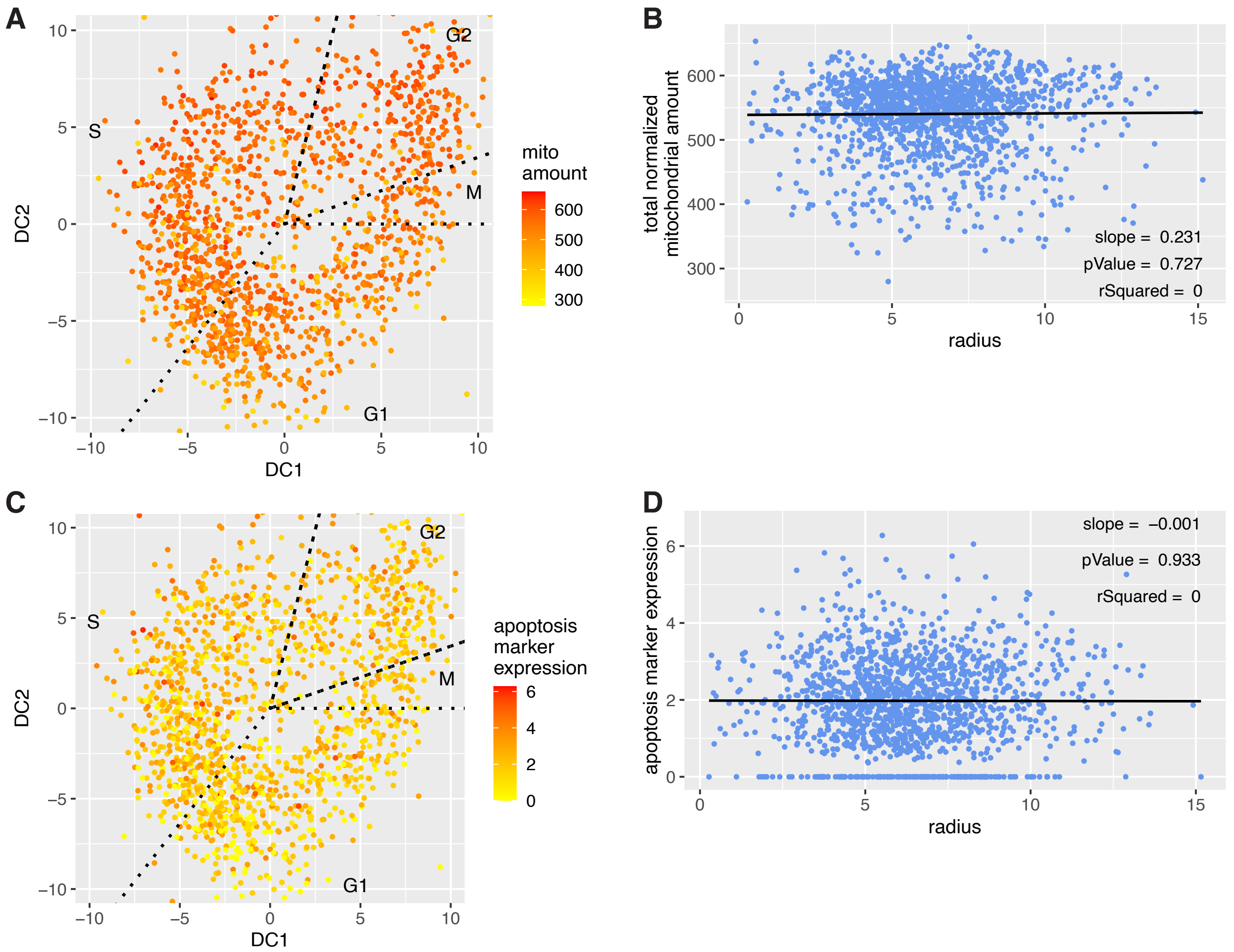

Appendix Figure S5.Placement of cells near the origin is not caused by apoptosis.
A The amount of mitochondrial gene expression can serve as an indicator for apoptotic behaviour (Mayer & Oberbauer, 2003).
B No significantly positive relationship between the radius and mitochondrial amount is contained in our data.
C+D The same conclusion is drawn from investigating marker genes that are known to be expressed during apoptosis.

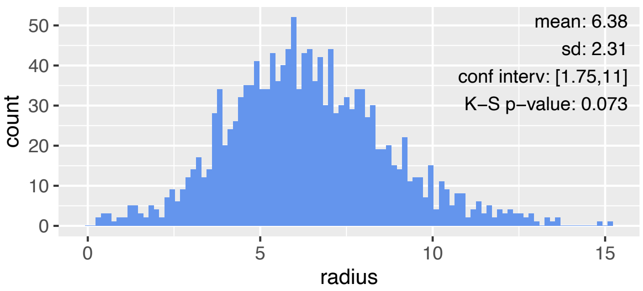

Appendix Figure S6.Statistically, the radius of the points of the data cloud fit a normal distribution, implying an annular shape.

In order to assess whether the 2-dimensional data cloud fits our proposed shapes, we consider the distribution of the radii. In case the cell cycle dimensions DC1 and DC2 were independent centered normal distributions, we would expect the data to cluster around the origin. The distribution of radii would then clearly peak at 0. In case of coordinated cyclic motion, we would expect the radii to have a broader distribution and a peak away from the origin. As to the distinction between a disk and an annulus, we should be able to distinguish between the two by constructing confidence intervals for the data. If the lower bound for the radius of that confidence interval is $\gg 0$, we consider the data to take the shape of an annulus. A disk would have a lower confidence bound close to or below 0. If the radii are normally distributed, construction of confidence intervals is straightforward by taking the interval $[\mu - 2\sigma, \mu + 2\sigma]$. Therefore, we introduce the Kolmogorov-Smirnov (K-S) test. A data distribution is considered normal by a K-S test if its p-value is > 0.05. We observe that for the Hela Data set 1.1 the distribution can statistically be categorized by a normal distribution. Therefore, the conclusion of annular shape is justified.

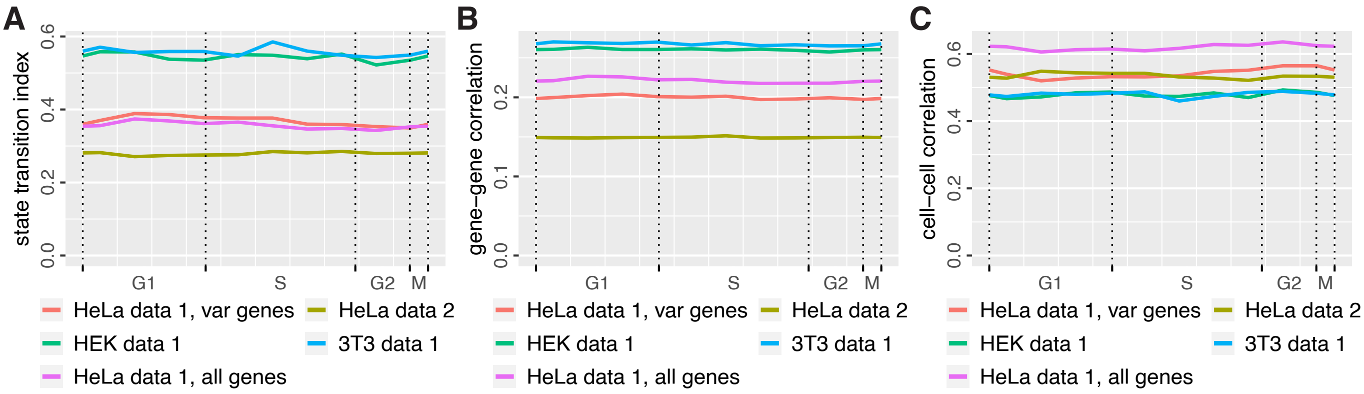

Appendix Figure S7.State transition index (Mojtahedi *et al.*, 2016) along the cell cycle does not indicate a critical state transition.

A The state transition index (ratio of gene-gene to cell-cell correlations) (Mojtahedi *et al.*, 2016) indicates a homogeneous stability. As a critical state transition is accompanied by a rise of the state transition index, we suspect that such a transition is not taking place along the cell cycle of immortalized cell lines.

B+C The two contributing factors (gene-gene and cell-cell correlation) to the state transition index are likewise stable along the cell cycle.

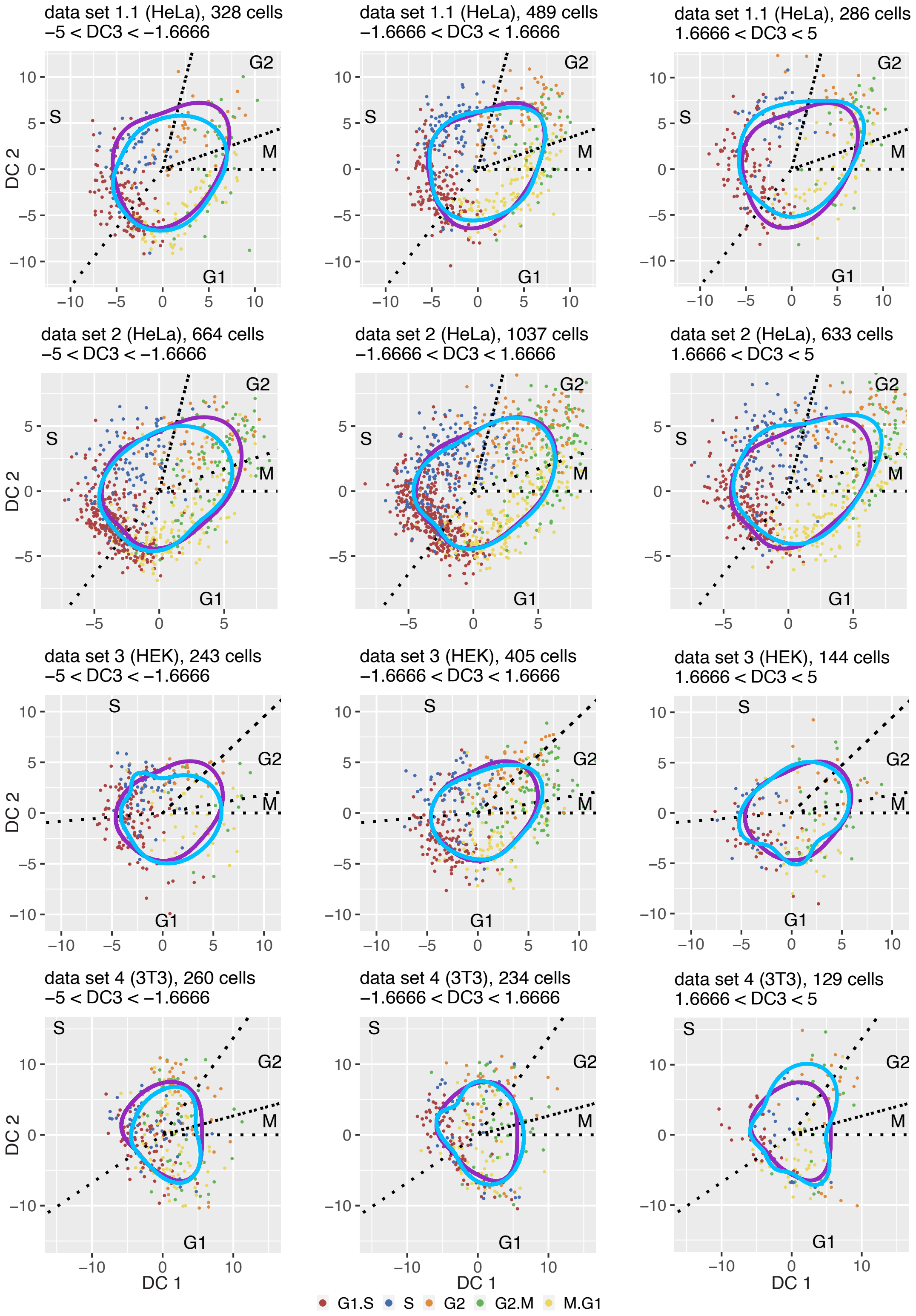

Appendix Figure S8.Average trajectories for different height ranges of the cylinder in four data sets.
The cylinder formed by the data cloud in DC1-DC2-DC3 is divided into three subsections along the cylinder axis (DC3). Each row corresponds to a different data set and each column signifies a different cylinder slice. In purple the average cell cycle of the respective population is displayed, whereas in light blue the average cell cycle trajectory of all cells displayed in a particular plot is shown. We observe that the average trajectories do not significantly differ from the population

average. This implies that a cell's placement along the cylinder axis does not significantly influence gene expression of the cell cycle.

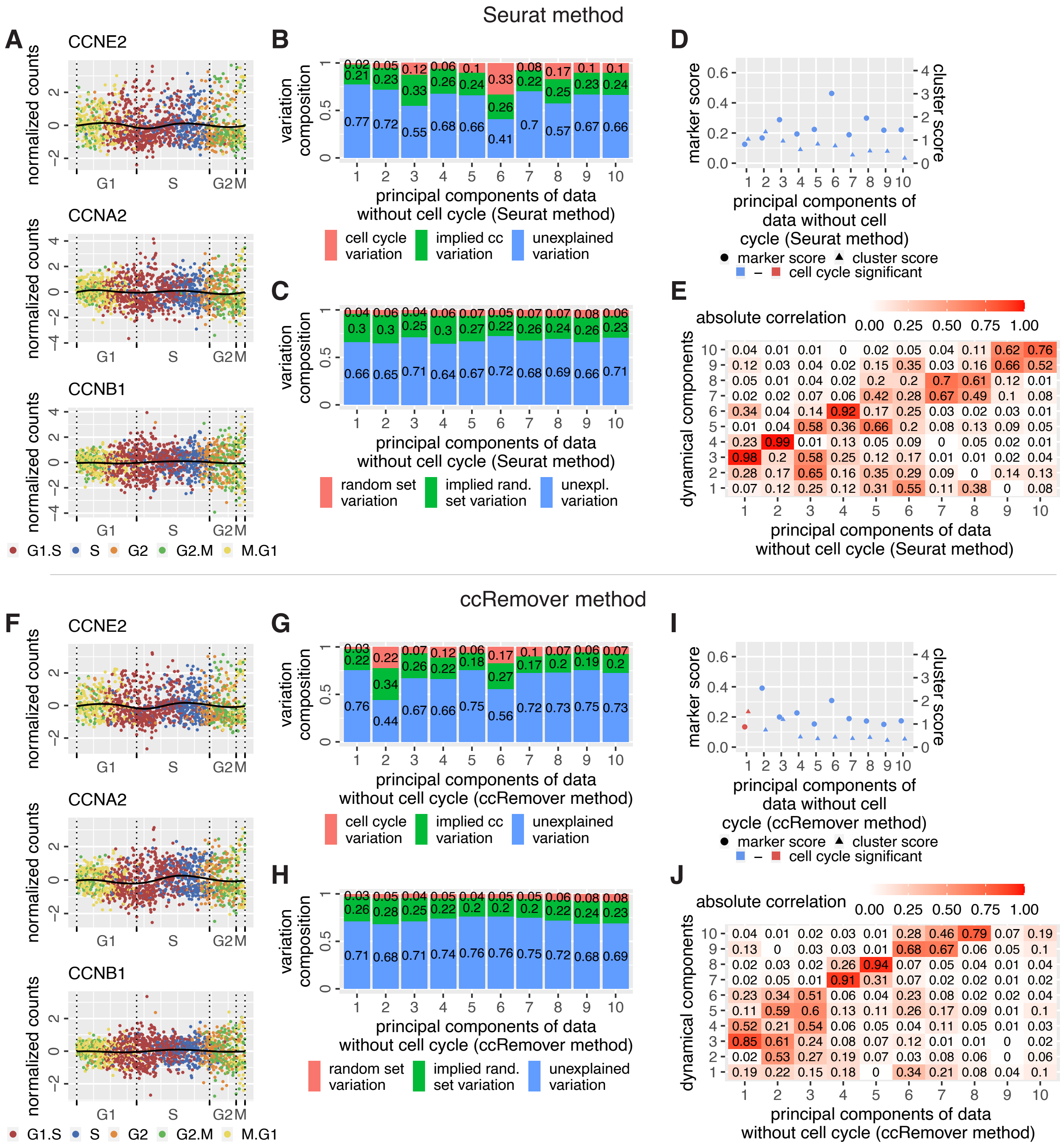

Appendix Figure S9.Other methods are less successful than Revelio at removing only cell cycle effects from data. The same characteristics shown in Fig 4, this time processed with the Seurat (Butler *et al*, 2018) package (panel A-E) and the ccRemover (Barron & Li, 2016) method (panel F-J). Due to technical incompatibilities we were not able to process the same data with scLVM (Buettner *et al*, 2015).

A The three cyclins do not exhibit obvious peaks after being processed by Seurat.

B-D We notice that there is still cell cycle contained in the data after processing with Seurat which is evident by the marker score (panel **B)** and the variation decomposition of cell cycle genes (panel **C**) which does not resemble a random gene set (panel **D**).

E The new principal components from the processed data by the Seurat method do not appear to have a clear counterpart in the dynamical components from the original data. This suggests that the applied method by Seurat has a noticeable effect on dimensions not influenced by the cell cycle which is not desired.
F The time courses after processing with ccRemover are almost indistinguishable from the previous ones by Seurat.
G-I The marker score (panel **G**) and variation decomposition of a cyclic gene set (panel **H**) again suggest some leftover cell cycle effects.
J The absence of strong correlations between the new principal components of the data after processing with ccRemover and the original dynamic components again suggests that gene expression beyond cell cycle effects is also influenced by ccRemover.

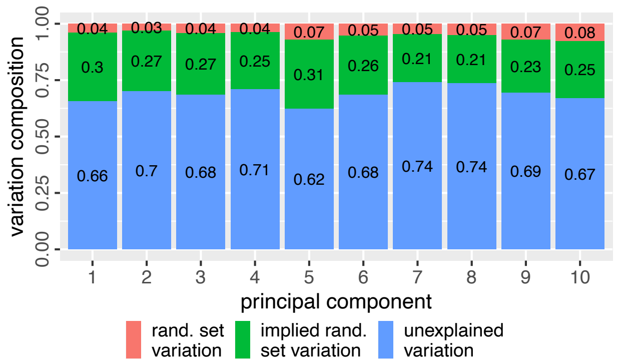

Appendix Figure S10.Variation decomposition with respect to a random gene set exhibits similar distributions across all components.

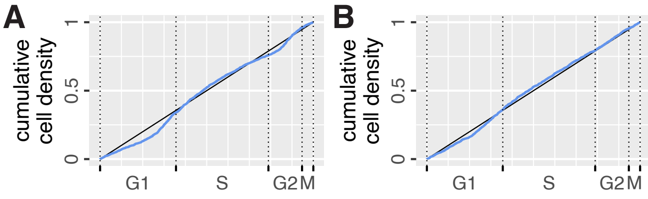

Appendix Figure S11.The cumulative phase space density of cells (blue) is reasonably well approximated by a uniform distribution (black).
A Hela data set 1.1 (only variable genes).
B Hela data set 1.2 (all genes detected during sequencing).

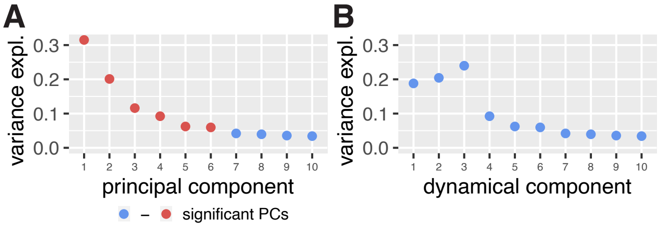

Appendix Figure S12.Variance explained per component (see section "Explained Variation and Significance of Principal Components" in Materials and Methods)..

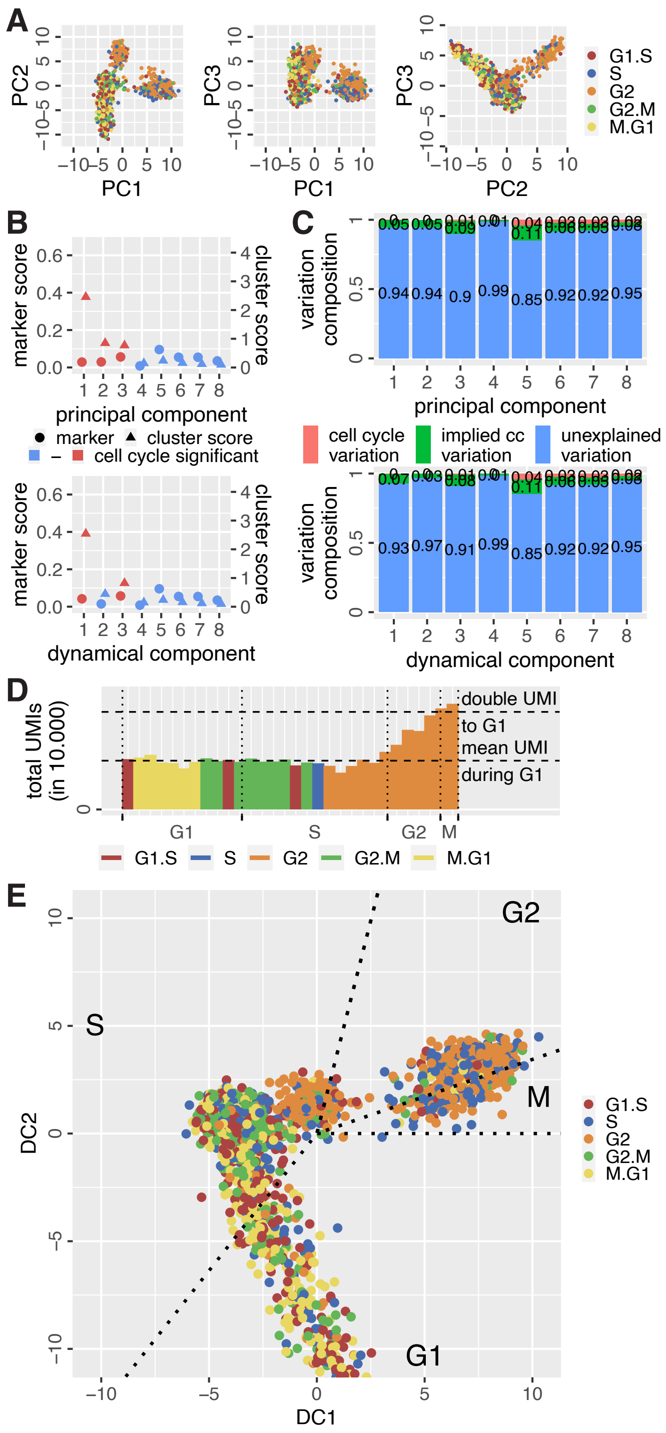

Appendix Figure S13. When processed with the Revelio algorithm, a negative control of non-cycling peripheral blood mononuclear cells (PBMC) exhibits no periodic data structure.
A Pairwise combinations of the first three PCs.
B The cluster and marker score for PC1 exhibit contradicting behaviour, suggesting that the detected signal is not caused by cell cycle. Similarly, the scores of the DCs do not suggest clear cell cycle structures.
C The variation decomposition for PCs and DCs are completely dominated by unexplained variation.
D The time course of total UMI counts inferred by angular position of the cells does suggest a doubling of UMI counts from beginning to end. This plot illustrates that it is important to consider all characteristics simultaneously. While this particular total UMI time course could in principle stem from cell cycle effects, the two-dimensional plot in DC space, the marker and cluster score and the variation decomposition all strongly contradict that assumption.
E The previously described rotation of PC space does not reveal any periodic structure in DC space.

- Alles J, Karaiskos N, Praktiknjo SD, Grosswendt S, Wahle P, Ruffault PL, Ayoub S, Schreyer L, Boltengagen A, Birchmeier C *et al* (2017) Cell fixation and preservation for droplet-based single-cell transcriptomics. *BMC Biol* 15: 44
- Barron M, Li J (2016) Identifying and removing the cell-cycle effect from single-cell RNA-Sequencing data. *Sci Rep* 6: 33892
- Buettner F, Natarajan KN, Casale FP, Proserpio V, Scialdone A, Theis FJ, Teichmann SA, Marioni JC, Stegle O (2015) Computational analysis of cell-to-cell heterogeneity in single-cell RNA-sequencing data reveals hidden subpopulations of cells. *Nat Biotechnol* 33: 155-160
- Butler A, Hoffman P, Smibert P, Papalexi E, Satija R (2018) Integrating single-cell transcriptomic data across different conditions, technologies, and species. *Nat Biotechnol* 36: 411-420
- Eden E, Lipson D, Yogev S, Yakhini Z (2007) Discovering motifs in ranked lists of DNA sequences. *PLoS Comput Biol* 3: e39
- Eden E, Navon R, Steinfeld I, Lipson D, Yakhini Z (2009) GOrilla: a tool for discovery and visualization of enriched GO terms in ranked gene lists. *BMC Bioinformatics* 10: 48
- Mayer B, Oberbauer R (2003) Mitochondrial regulation of apoptosis. *News Physiol Sci* 18: 89-94
- Mojtahedi M, Skupin A, Zhou J, Castano IG, Leong-Quong RY, Chang H, Trachana K, Giuliani A, Huang S (2016) Cell Fate Decision as High-Dimensional Critical State Transition. *PLoS Biol* 14: e2000640
