# Supplementary material for: The transcriptome dynamics of single cells during the cell cycle
Source: Mol Syst Biol. 2020 Nov 18;16(11):e9946. doi: 10.15252/msb.20209946 (PMC7672610; doi:10.15252/msb.20209946)
Supplement: Supplementary file 2 — Expanded View Figures PDF [file MSB-16-e9946-s002.pdf]

Expanded View Figures

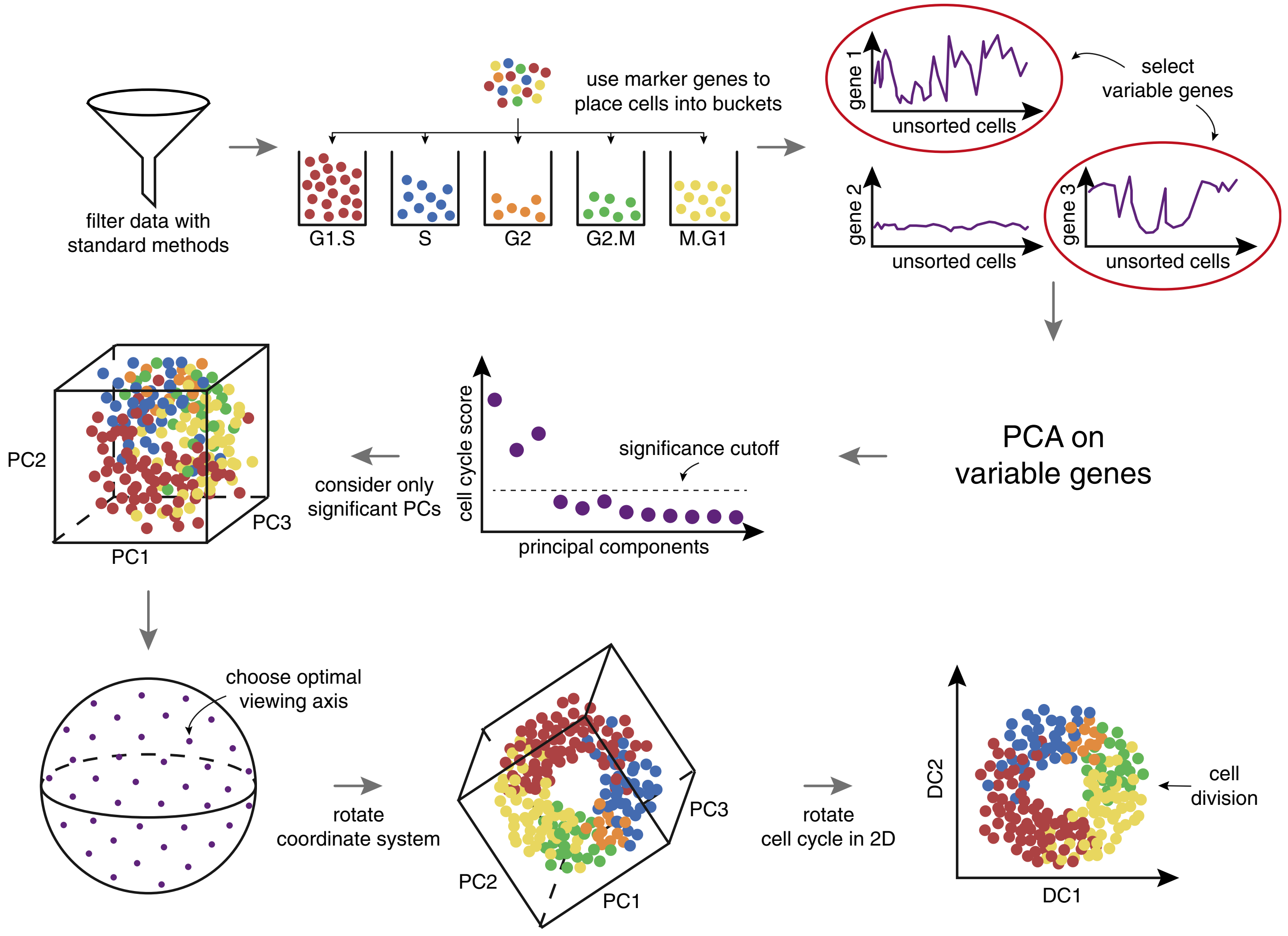

Figure EV1. The principle steps of our algorithm Revelio for extracting the cell cycle from the data.

After the data are filtered by standard methods, we divide the cells into buckets with the help of marker genes (Whitfield *et al*, 2002; Macosko *et al*, 2015). Next, we select variable genes (Butler *et al*, 2018) and apply PCA on the reduced data set. Afterward, we utilize a cell cycle marker and cluster score (Materials and Methods) to judge which PCs are influenced by the cell cycle. The significant PCs are used to construct three-dimensional subspaces. We then choose an optimal viewing axis by minimizing the cell cycle score along the viewing axis (Materials and Methods). The coordinate system is rotated linearly and the cell cycle is obtained only within the plane spanned by the first two new axes (DC1, DC2). Within the DC1-DC2 plane, we estimate the time point of cell division and rotate the cell cycle plane accordingly (Materials and Methods).

© 2020 The Authors

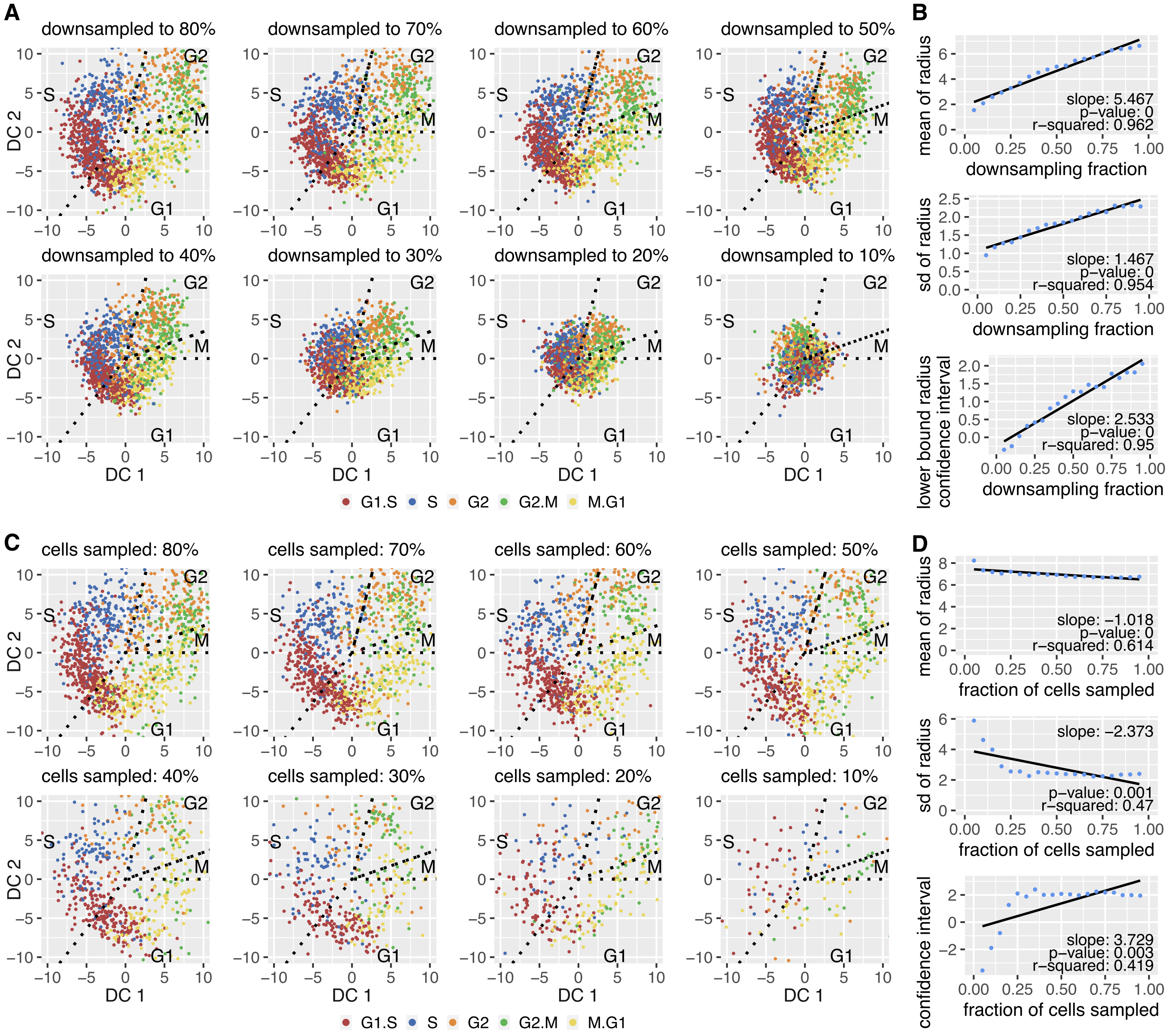

Figure EV2. Downsampling the UMIs leads to a continuously collapsing cell cycle while sampling cells maintains the cycle until a threshold is reached.

A We downsample (Griffiths *et al*, 2018; Lun *et al*, 2019) the data set to different percentages of the total UMI for each cell and run Revelio on the new data. The cell cycle is slowly collapsing toward the origin with decreasing information.

B Statistics on the radius of the data points seen in panel (A) confirm the previous conclusion. (top) Mean of the radius. (middle) Standard deviation of the radius. (bottom) The lower bound of the confidence interval of the radius. For all of these characteristics, a clear linear relationship to the downsampling percentage is apparent.

C We now sample a certain percentage of the cells while keeping the information within each cell untouched. The cell cycle now does not appear to collapse anymore but becomes less clear with fewer cells available.

D The same characteristics as in panel B now suggest that the cell cycle stays intact and in principle unchanged as long as at least 25% of the initial amount of cells are maintained. Afterward, the circular cell cycle signal breaks down rapidly with the decreasing amount of cells.

 © 2020 The Authors

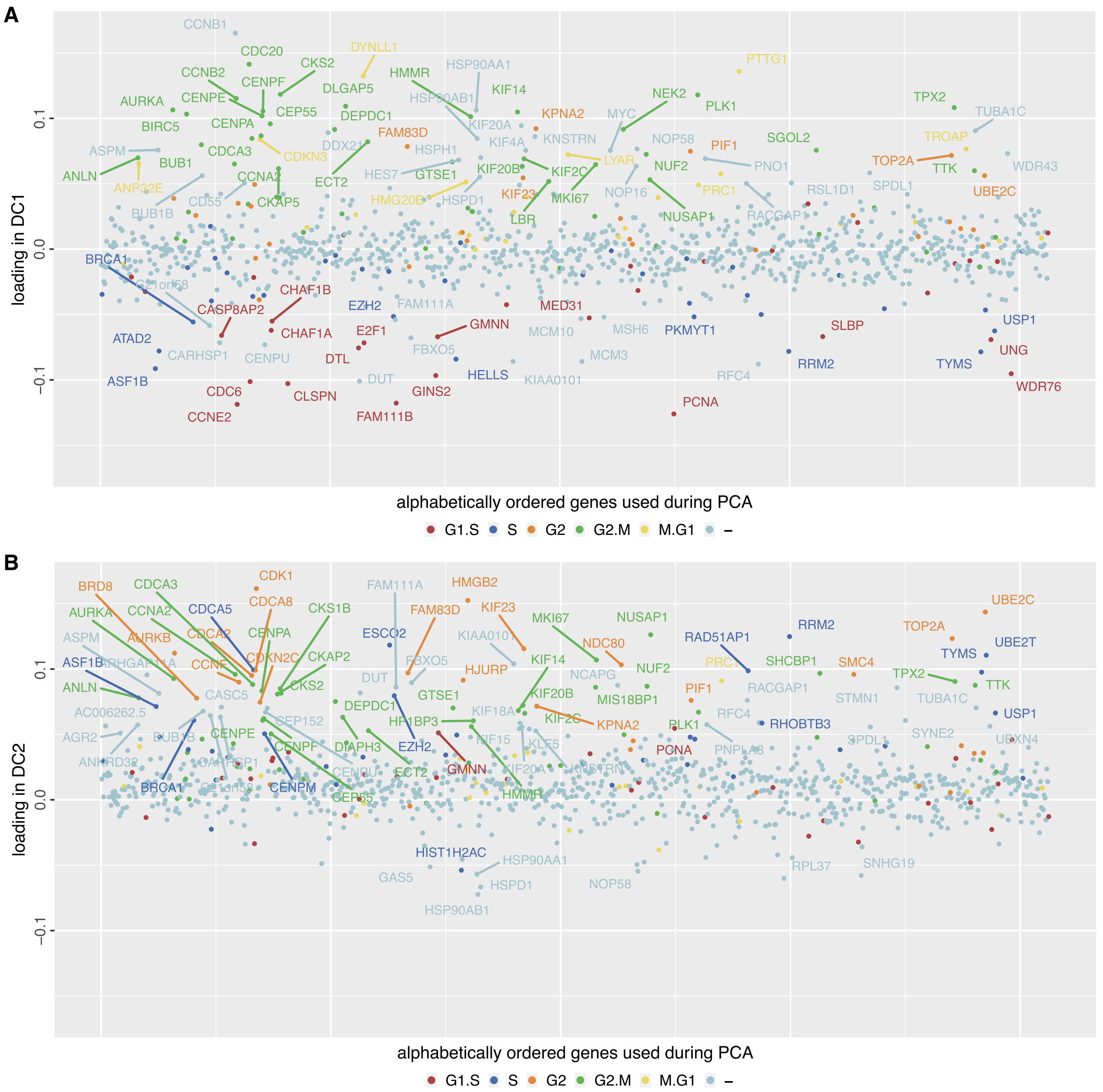

Figure EV3. Genes associated with dynamical components mostly correspond to known oscillating genes.

A Weights of genes that span DC1. Colors indicate if a gene is known to be oscillating from Whitfield *et al* (2002). Negative values (corresponding to the left part of the x-axis of Fig 1B) are mostly associated with G1-S and S while positive values (right part of x-axis in Fig 1B) correspond to M phase.

B Weights of genes that span DC2. Positive values are associated with the transition S-G2 and M phase. Very few genes have significant negative weights for DC2. Within our cell cycle from Fig 1B, the lower part of the y-axis corresponds to G1 phase. Thus, this plot confirms that almost no variable genes are active during G1 phase making it difficult to classify cycling cells into G1 because of the lack of marker genes.

© 2020 The Authors

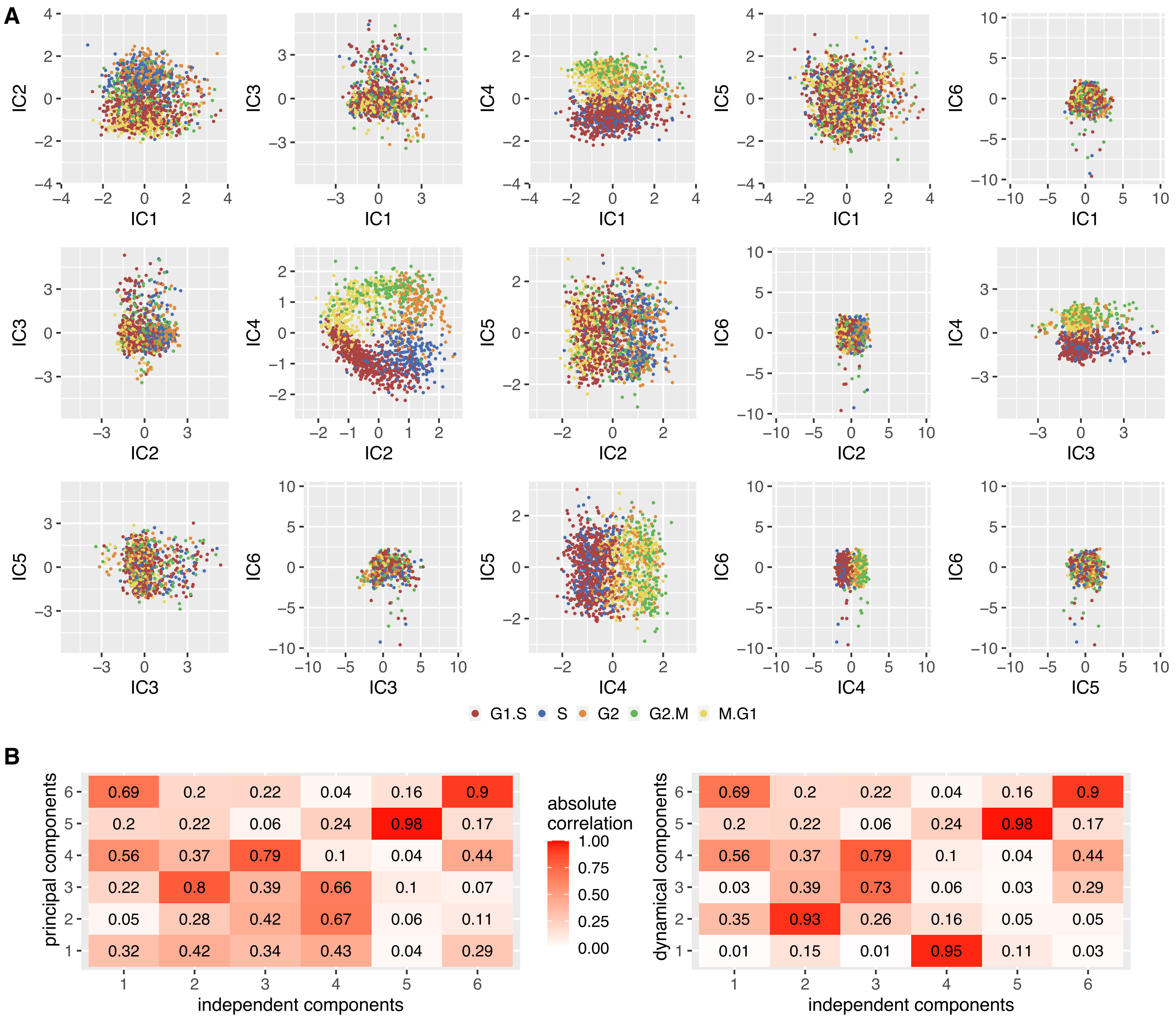

Figure EV4. Independent component analysis suggests two independent sources of the cell cycle which are highly correlated to DC1 and DC2.

A Pairwise projections of independent components IC1 to IC6. In this run of ICA, the two components representing the cell cycle appear to be IC2 and IC4.

B Correlation of independent components with principal components (left) and dynamical components (right). We observe strong correlation of IC2 and IC4 to DC2 and DC1, respectively. DC1 and DC2 show no strong correlation to other than these two components. Before rotation, PC1 to PC3 exhibit multiple strong correlations to ICs. The correlations of PC4-PC6 to the ICs are the same as DC4-DC6 to the ICs since only PC1-PC3 are involved in the rotation performed by Revelio and thus PC4 and all higher order PCs are equal to their DC counterpart.

 © 2020 The Authors

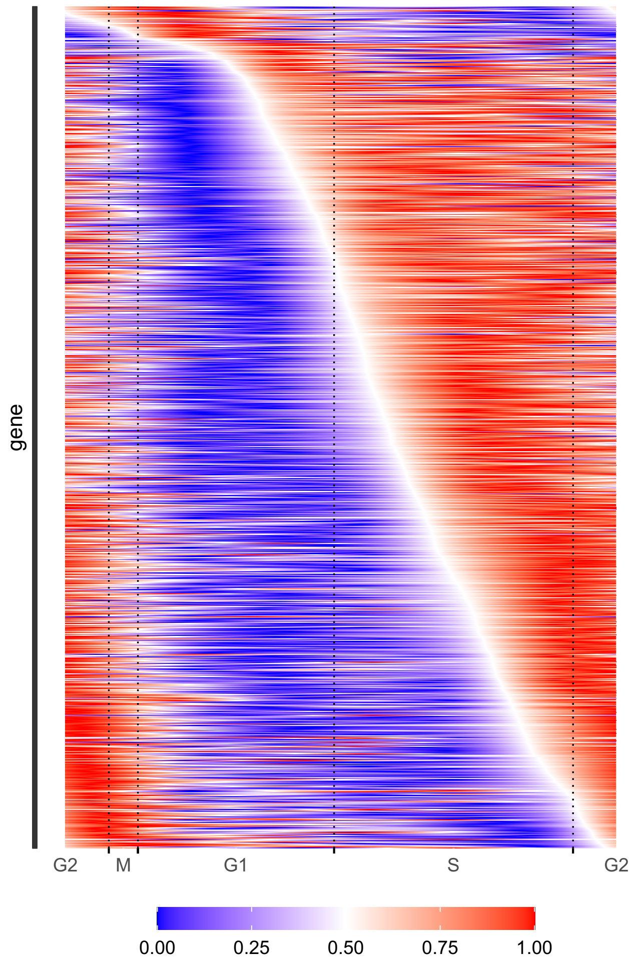

Figure EV5. The distribution of transcription initiation of variable genes is constant from mid G1 to mid G2 phase.

We normalize the time courses of the variable genes to the interval [0,1] $\left(\frac{\text{signal}-\text{minimum}}{\text{maximum}-\text{minimum}}\right)$ and order by the time when 0.5 is crossed from below (white line). The slope of the white line reports the rate of transcription onsets per unit time. The steeper the slope, the higher is the rate. We see that this rate is almost constant from the middle of G1 to the middle of G2. It decreases by about a factor 5 between the middle of G2 to the middle of G1 including M phase and cell division. Hence, about 93% of the variable genes have their transcription onset almost equally distribute over 70% of the cycle and 7% start with a lower rate within 30% of the cycle. The rate of onsets is almost constant within both sections. This supports our optimality criterion of avoidance of sudden changes, since we see a decrease from mid G2 to mid G1 phase.

© 2020 The Authors
