# Supplementary material for: The transcriptome dynamics of single cells during the cell cycle
Source: Mol Syst Biol. 2020 Nov 18;16(11):e9946. doi: 10.15252/msb.20209946 (PMC7672610; doi:10.15252/msb.20209946)
Supplement: Supplementary file 4 — Review Process File [file MSB-16-e9946-s004.pdf]

molecular systems biology

The transcriptome dynamics of single cells during the cell cycle

Daniel Schwabe, Sara Formichetti, Jan Philipp Junker, Martin Falcke, and Nikolaus Rajewsky
DOI: 10.15252/msb.20209946

Corresponding author(s): Nikolaus Rajewsky (rajewsky@mdc-berlin.de) , Martin Falcke (martin.falcke@mdc-berlin.de)

Review Timeline:

Submission Date:	24th Aug 20
Editorial Decision:	4th Sep 20
Revision Received:	12th Sep 20
Accepted:	22nd Sep 20

Editor: Thomas Lemberger (MSB)

Transaction Report:

(Note: With the exception of the correction of typographical or spelling errors that could be a source of ambiguity, letters and reports are not edited. Depending on transfer agreements, referee reports obtained elsewhere may or may not be included in this compilation. Referee reports are anonymous unless the Referee chooses to sign their reports.)

The initial reviewers' comments and authors' responses are not available with this article, as the initial review process took place with another journal.

1st Editorial Decision 4th Sep 2020

Thank you again for submitting your work to Molecular Systems Biology. We have now heard back from the reviewer who also served as referee #2 at the previous journal and whom we asked to comment on your reply to the points raised by all reviewers. As you will see, this reviewer is globally positive but still raises a few remaining issues that need further clarification with suitable amendments to the text.

REFEREE REPORTS

Reviewer #2:

Schwabe et al present a new method to analyze the dynamics of the transcriptome of proliferating single cells, using deep single-cell RNAseq, and claim to uncover a general design principle of the cell cycle. As I mentioned in my previous review for Nature, I appreciate the analysis and personally find this new approach compelling. I am mostly satisfied with the answers to my previous comments. The revision has clearly improved the paper. In particular, the new Figure 4, added in response to the comments of reviewer 1, is a valuable addition to the manuscript. I only have the following three remaining issues:

1. In response to comment 7 of my previous review (and to the analogous comment 6 of reviewer 1), the authors have retracted their statement that cells near the origin are dead cells. In fact, their new Extended Data Fig. 7 shows the opposite, namely that cells near the origin are equally likely to be alive than cells far away from the origin. However, the authors do not extract any conclusion from this result, neither in their response to the reviewers nor in the revised manuscript (where they don't even cite the new figure, if I'm not wrong). The way in which these cells fit within the paper's message should be addressed explicitly in the main text (and the figure should be cited and commented upon).

2. I must insist on my comment 14 (which is very minor, but nevertheless). In line 462-463 of the revised paper the authors maintain that "the trajectory of a dynamical system cannot intersect with itself". This sentence would be clear enough in an applied mathematics journal dealing with ODE models, but given the large amount of randomness exhibited in all data figures shown in this paper (understandably, since it's real data), one should not assume that readers would automatically interpret a "dynamical system" as a "deterministic dynamical system". I would ask the authors to explicitly include the adjective "deterministic" in that sentence, to avoid confusion.

3. Finally, in the response to comment 1 of reviewer 1 (page 3 of their response to reviewers document), the correlation coefficients between all ICs and PCs 4-6 are identical to the correlation coefficients between the ICs and DCs 4-6 (including two very high correlations between PC5-IC1 and PC6-IC3, which seem to counter the authors' claims, although of course we're talking about high-order PCs). It seems hard to believe that these 18 coefficients are identical by chance. Can the authors clarify this point?

Molecular System Biology Review:

Reviewer #2:

Schwabe et al present a new method to analyze the dynamics of the transcriptome of proliferating single cells, using deep single-cell RNAseq, and claim to uncover a general design principle of the cell cycle. As I mentioned in my previous review for Nature, I appreciate the analysis and personally find this new approach compelling. I am mostly satisfied with the answers to my previous comments. The revision has clearly improved the paper. In particular, the new Figure 4, added in response to the comments of reviewer 1, is a valuable addition to the manuscript. I only have the following three remaining issues:

1. In response to comment 7 of my previous review (and to the analogous comment 6 of reviewer 1), the authors have retracted their statement that cells near the origin are dead cells. In fact, their new Extended Data Fig. 7 shows the opposite, namely that cells near the origin are equally likely to be alive than cells far away from the origin. However, the authors do not extract any conclusion from this result, neither in their response to the reviewers nor in the revised manuscript (where they don't even cite the new figure, if I'm not wrong). The way in which these cells fit within the paper's message should be addressed explicitly in the main text (and the figure should be cited and commented upon).

Reply:

We believe that cells near the origin occur due to multiple sources of technical noise. The amount of cells near the origin is very small (as the distribution of radii in Appendix Figure S6 shows). Within the setting of real experimental data. such occurrence is reasonable. We have added corresponding comments classifying these cells to lines 120-123, page 4 of the manuscript. In our view, the only biological explanation would have been that they are dead cells, which we excluded in Appendix Figure S5 (formerly Extended Data Fig. 7).

We would like to point out that we did not claim cells in the center of the cycle to be dead cells in the original manuscript. We originally stated: “However, cells nearby the origin of the trajectory are biologically implausible as these cells would reside simultaneously in all cell cycle phases (we ruled out that these cells are simply dead cells by using standard apoptosis markers).” Only the evidence for this statement was missing (but was rectified in our previous response following the reviewer’s comment). We now reference the corresponding figures also in lines 120-123, page 4 of the manuscript

2. I must insist on my comment 14 (which is very minor, but nevertheless). In line 462-463 of the revised paper the authors maintain that "the trajectory of a dynamical system cannot intersect with itself". This sentence would be clear enough in an applied mathematics journal dealing with ODE models, but given the large amount of randomness exhibited in all data figures shown in this paper (understandably, since it's real data), one should not assume that readers would automatically interpret a "dynamical system" as a "deterministic dynamical system". I would ask the authors to explicitly include the adjective "deterministic" in that sentence, to avoid confusion.

Reply:

We agree with the reviewer’s comment and have added the clarification as requested. Due to moving this section to Box 1, the comment is now found in line 1171 on page 31.

3. Finally, in the response to comment 1 of reviewer 1 (page 3 of their response to reviewers document), the correlation coefficients between all ICs and PCs 4-6 are identical to the correlation coefficients between the ICs and DCs 4-6 (including two very high correlations between PC5-IC1 and PC6-IC3, which seem to counter the authors' claims, although of course we're talking about high-order PCs). It seems hard to believe that these 18 coefficients are identical by chance. Can the authors clarify this point?

Reply:

This is indeed not a coincidence. In the considered data set, only PC1-PC3 are involved in the additional rotation after PCA performed by Revelio. Thus, PC4 and all higher order PCs are identical to DC4 and all higher order DCs. Hence, their correlation to all ICs is identical. We have added similar comments to the corresponding figure caption of Figure EV4 (formerly Extended Data Fig. 11) lines 1152-1154, page 29 of the manuscript to clarify this connection for future readers.

The existence of high correlation between any of the ICs and higher order PCs is in our view not countering our claims. The ICs represent the data separated into sources that are maximally statistically independent. However, the ICs are in no way ordered by importance. We observe that exactly two of these sources (IC2 and IC4) are highly correlated to DC1 and DC2, which are exactly those ICs that also form a circular data structure in Figure EV4.

The fact that we find other ICs (which are not IC2 and IC4) that also have high correlation with particular PCs/DCs only indicates that those PCs/DCs are very closely related to additional independent sources of variation, which are different from the cell cycle induced variation.

Accepted **22nd Sep 2020**

Thank you again for sending us your revised manuscript. We are now satisfied with the modifications made and I am pleased to inform you that your paper has been accepted for publication.

EMBO PRESS

YOU MUST COMPLETE ALL CELLS WITH A PINK BACKGROUND ↓

PLEASE NOTE THAT THIS CHECKLIST WILL BE PUBLISHED ALONGSIDE YOUR PAPER

Corresponding Author Name: Nikolaus Rajewsky, Martin Falcke
Journal Submitted to: Molecular Systems Biology
Manuscript Number: MSB-20-9946

Reporting Checklist For Life Sciences Articles (Rev. June 2017)

This checklist is used to ensure good reporting standards and to improve the reproducibility of published results. These guidelines are consistent with the Principles and Guidelines for Reporting Preclinical Research issued by the NIH in 2014. Please follow the journal's authorship guidelines in preparing your manuscript.

A- Figures
1. Data
The data shown in figures should satisfy the following conditions:

- the data were obtained and processed according to the field's best practice and are presented to reflect the results of the experiments in an accurate and unbiased manner.
- figure panels include only data points, measurements or observations that can be compared to each other in a scientifically meaningful way.
- graphs include clearly labeled error bars for independent experiments and sample sizes. Unless justified, error bars should not be shown for technical replicates.
- if n< 5, the individual data points from each experiment should be plotted and any statistical test employed should be justified
- Source Data should be included to report the data underlying graphs. Please follow the guidelines set out in the author ship guidelines on Data Presentation.

2. Captions
Each figure caption should contain the following information, for each panel where they are relevant:

- a specification of the experimental system investigated (eg cell line, species name).
- the assay(s) and method(s) used to carry out the reported observations and measurements
- an explicit mention of the biological and chemical entity(ies) that are being measured.
- an explicit mention of the biological and chemical entity(ies) that are altered/varied/perturbed in a controlled manner.
- the exact sample size (n) for each experimental group/condition, given as a number, not a range;
- a description of the sample collection allowing the reader to understand whether the samples represent technical or biological replicates (including how many animals, litters, cultures, etc.).
- a statement of how many times the experiment shown was independently replicated in the laboratory.
- definitions of statistical methods and measures:
  - common tests, such as t-test (please specify whether paired vs. unpaired), simple $\chi 2$ tests, Wilcoxon and Mann-Whitney tests, can be unambiguously identified by name only, but more complex techniques should be described in the methods section;
  - are tests one-sided or two-sided?
  - are there adjustments for multiple comparisons?
  - exact statistical test results, e.g., P values = x but not P values < x;
  - definition of 'center values' as median or average;
  - definition of error bars as s.d. or s.e.m.

Any descriptions too long for the figure legend should be included in the methods section and/or with the source data.

In the pink boxes below, please ensure that the answers to the following questions are reported in the manuscript itself. Every question should be answered. If the question is not relevant to your research, please write NA (non applicable). We encourage you to include a specific subsection in the methods section for statistics, reagents, animal models and human subjects.

USEFUL LINKS FOR COMPLETING THIS FORM

http://www.antibodypedia.com
http://1degreebio.org
http://www.equator-network.org/reporting-guidelines/improving-bioscience-research-repor
http://grants.nih.gov/grants/olaw/olaw.htm
http://www.mrc.ac.uk/Ourresearch/Ethicsresearchguidance/Useofanimals/index.htm
http://ClinicalTrials.gov
http://www.consort-statement.org
http://www.consort-statement.org/checklists/view/32-consort/66-title
http://www.equator-network.org/reporting-guidelines/reporting-recommendations-for-tumo
http://datadryad.org
http://figshare.com
http://www.ncbi.nlm.nih.gov/gap
http://www.ebi.ac.uk/ega
http://biomodels.net/
http://biomodels.net/miriam/
http://jjj.biochem.sun.ac.za
http://oba.od.nih.gov/biosecurity/biosecurity_documents.html
http://www.selectagents.gov/

B- Statistics and general methods

Please fill out these boxes ↓ (Do not worry if you cannot see all your text once you press return)

1.a. How was the sample size chosen to ensure adequate power to detect a pre-specified effect size?	Existing scRNA-Seq data generated in our lab and published by other labs were used to understand the expected variance of read counts mapped to a single gene (given a certain sequencing depth). Based on this known parameter, we predicted the minimum number of single-cells that was necessary to make observations on gene expression statistically reliable.
1.b. For animal studies, include a statement about sample size estimate even if no statistical methods were used.	Not applicable.
2. Describe inclusion/exclusion criteria if samples or animals were excluded from the analysis. Were the criteria pre-established?	Only low quality cells were excluded as described in the Materials and Methods section.
3. Were any steps taken to minimize the effects of subjective bias when allocating animals/samples to treatment (e.g. randomization procedure)? If yes, please describe.	No steps taken as it is not relevant to this study.
For animal studies, include a statement about randomization even if no randomization was used.	No randomization was used.
4.a. Were any steps taken to minimize the effects of subjective bias during group allocation or/and when assessing results (e.g. blinding of the investigator)? If yes please describe.	No steps taken as it is not relevant to this study.
4.b. For animal studies, include a statement about blinding even if no blinding was done	No blinding was done.
5. For every figure, are statistical tests justified as appropriate?	Yes.
Do the data meet the assumptions of the tests (e.g., normal distribution)? Describe any methods used to assess it.	Yes.
Is there an estimate of variation within each group of data?	Yes.

Is the variance similar between the groups that are being statistically compared?	No groups are statistically compared.

C- Reagents

6. To show that antibodies were profiled for use in the system under study (assay and species), provide a citation, catalog number and/or clone number, supplementary information or reference to an antibody validation profile. e.g., Antibodypedia (see link list at top right), 1DegreeBio (see link list at top right).	Not applicable.
7. Identify the source of cell lines and report if they were recently authenticated (e.g., by STR profiling) and tested for mycoplasma contamination.	Data set 1: Laudadio, I. et al. 2019, "AGO2 promotes telomerase activity and interaction between the telomerase components TERT and TERC". EMBO Rep 20, doi:10.15252/embr.201845969. All cell lines tested negative for mycoplasma contamination.

* for all hyperlinks, please see the table at the top right of the document

D- Animal Models

8. Report species, strain, gender, age of animals and genetic modification status where applicable. Please detail housing and husbandry conditions and the source of animals.	Not applicable.
9. For experiments involving live vertebrates, include a statement of compliance with ethical regulations and identify the committee(s) approving the experiments.	Not applicable.
10. We recommend consulting the ARRIVE guidelines (see link list at top right) (PLoS Biol. 8(6), e1000412, 2010) to ensure that other relevant aspects of animal studies are adequately reported. See author guidelines, under 'Reporting Guidelines'. See also: NIH (see link list at top right) and MRC (see link list at top right) recommendations. Please confirm compliance.	Not applicable.

E- Human Subjects

11. Identify the committee(s) approving the study protocol.	Not applicable.
12. Include a statement confirming that informed consent was obtained from all subjects and that the experiments conformed to the principles set out in the WMA Declaration of Helsinki and the Department of Health and Human Services Belmont Report.	Not applicable.
13. For publication of patient photos, include a statement confirming that consent to publish was obtained.	Not applicable.
14. Report any restrictions on the availability (and/or on the use) of human data or samples.	Not applicable.
15. Report the clinical trial registration number (at ClinicalTrials.gov or equivalent), where applicable.	Not applicable.
16. For phase II and III randomized controlled trials, please refer to the CONSORT flow diagram (see link list at top right) and submit the CONSORT checklist (see link list at top right) with your submission. See author guidelines, under 'Reporting Guidelines'. Please confirm you have submitted this list.	Not applicable.
17. For tumor marker prognostic studies, we recommend that you follow the REMARK reporting guidelines (see link list at top right). See author guidelines, under 'Reporting Guidelines'. Please confirm you have followed these guidelines.	Not applicable.

F- Data Accessibility

18: Provide a "Data Availability" section at the end of the Materials & Methods, listing the accession codes for data generated in this study and deposited in a public database (e.g. RNA-Seq data: Gene Expression Omnibus GSE39462, Proteomics data: PRIDE PXD000208 etc.) Please refer to our author guidelines for 'Data Deposition'.<br><br>Data deposition in a public repository is mandatory for:<br>a. Protein, DNA and RNA sequences<br>b. Macromolecular structures<br>c. Crystallographic data for small molecules<br>d. Functional genomics data<br>e. Proteomics and molecular interactions	A data availability section is provided in the manuscript. RNA-Seq data: Gene Expression Omnibus GSE142277 (https://www.ncbi.nlm.nih.gov/geo/query/acc.cgi?acc=GSE142277); RNA-Seq data: Gene Expression Omnibus GSE142356 (https://www.ncbi.nlm.nih.gov/geo/query/acc.cgi?acc=GSE142356)
19. Deposition is strongly recommended for any datasets that are central and integral to the study; please consider the journal's data policy. If no structured public repository exists for a given data type, we encourage the provision of datasets in the manuscript as a Supplementary Document (see author guidelines under 'Expanded View' or in unstructured repositories such as Dryad (see link list at top right) or Figshare (see link list at top right).	All data is available in the manuscript.
20. Access to human clinical and genomic datasets should be provided with as few restrictions as possible while respecting ethical obligations to the patients and relevant medical and legal issues. If practically possible and compatible with the individual consent agreement used in the study, such data should be deposited in one of the major public access-controlled repositories such as dbGAP (see link list at top right) or EGA (see link list at top right).	All data is available in the manuscript.
21. Computational models that are central and integral to a study should be shared without restrictions and provided in a machine-readable form. The relevant accession numbers or links should be provided. When possible, standardized format (SBML, CellML) should be used instead of scripts (e.g. MATLAB). Authors are strongly encouraged to follow the MIRIAM guidelines (see link list at top right) and deposit their model in a public database such as Biomodels (see link list at top right) or JWS Online (see link list at top right). If computer source code is provided with the paper, it should be deposited in a public repository or included in supplementary information.	All computer code is available at https://github.com/danielschw188/Revelio

G- Dual use research of concern

22. Could your study fall under dual use research restrictions? Please check biosecurity documents (see link list at top right) and list of select agents and toxins (APHIS/CDC) (see link list at top right). According to our biosecurity guidelines, provide a statement only if it could.	Not applicable.
